# Simple and Rapid Microwave‐Assisted Suzuki–Miyaura Cross‐coupling in Betaine/Glycerol Natural Eutectic Solvent

**DOI:** 10.1002/open.202500138

**Published:** 2025-06-17

**Authors:** Chefikou Salami, Leslie Boudesocque‐Delaye, Pierre‐Olivier Delaye, Emilie Thiery

**Affiliations:** ^1^ Laboratoire Synthèse et Isolement de Molécules Bioactives (SIMBA, EA 7502) Faculté de Pharmacie Université de Tours Parc de Grandmont, 31 avenue Monge 37200 Tours France

**Keywords:** eutectic solvents, microwaves, Suzuki–Miyaura cross‐coupling

## Abstract

For some years now, eutectic solvents have been seen as an alternative to conventional organic solvents and ionic liquids. When the components are of natural origin, they meet the challenges of green chemistry. Although eutectic solvents are used in many synthetic reactions today, few references reported their use in microwave‐assisted reactions. To explore their potential and diversify their applications, Suzuki–Miyaura coupling was studied in eutectic solvents under microwave irradiation. After optimization using a design of experiment based on response surface methodology, the most efficient conditions were found to be betaine: glycerol (1:4, mol mol^−1^) as eutectic solvent, PdCl_2_dppf.CH_2_Cl_2_ as catalyst, Na_2_CO_3_ as base, and microwave heating at 129 °C for 15 min. The results showed an increase in reactivity with very rapid reaction kinetics. Furthermore, the catalyst in the eutectic solvent was recycled, and a gram‐scale reaction was performed as an example. Finally, this new methodology was evaluated using CHEM21 metrics.

## Graphical abstract

Suzuki–Miyaura coupling in a microwave‐assisted natural eutectic solvent was studied: optimization by design of experiments with response surface methodology, recycling, exemplification, scale‐up, and evaluation with CHEM21 toolkit for a sustainable methodology.

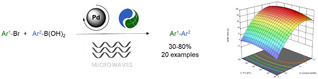

## 1 Introduction

**Figure 1:**
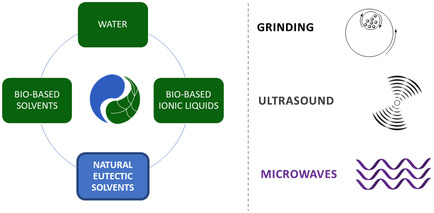
Examples of green solvents and eco‐friendly activation methods: this work combines natural eutectic solvents and microwave heating.

The 12 principles of green chemistry proposed by Anastas et al. aim to minimize the risks and environmental impact of chemical processes.^[^ ^1^ ^]^ They guide researchers in the development of more sustainable methodologies. Reaction solvents significantly influence the environmental impact, cost, health, and safety issues of chemical processes.^[^ ^2^ ^]^ In particular, principle five focuses on the replacement of conventional organic solvents with safer and more sustainable alternatives. In the absence of an universal solvent, several alternatives have been reported in the literature (**Figure** **1**). Water as a reaction medium in organic chemistry is an attractive solution, as it is inexpensive, nontoxic, and nonflammable.^[^ ^3^, ^4^, ^5^, ^6^ ^]^ The main difficulty lies in the insolubility of many organic compounds, which can lead to the use of phase transfer agents or ligands, or to carrying out reactions at high temperatures. Ethereal solvents such as 2‐methyltetrahydrofuran, produced from renewable resources, can advantageously replace tetrahydrofuran or diethyl ether in organic reactions.^[^ ^7^, ^8^ ^]^ Since the 2000s, the scientific community has shown a growing interest in ionic liquids, which have been shown to be effective in numerous organic reactions.^[^ ^9^, ^10^ ^]^ Their advantages include low vapor pressure, high thermal, chemical stability, and nonflammability. However, the toxicity of these solvents has been questioned in some studies,^[^ ^11^, ^12^ ^]^ prompting a move towards a new generation of bio‐based ionic liquids.^[^ ^13^, ^14^, ^15^ ^]^ Since the 2010s, deep eutectic solvents (DES) have been regarded as a promising alternative due to their nonvolatility, inflammability, and lower toxicity compared to conventional organic solvents.^[^ ^16^, ^17^, ^18^ ^]^ They are widely used in the extraction of biomass and, more recently, in organic synthesis and catalysis for multicomponent reactions, condensations, and organometallic couplings.^[^ ^19^ ^]^ Different classes of DES are reported (I– V) depending on their composition. However, they are originally described as a mixture of Lewis acids and Brønsted bases. The most common definition is based on a combination of several compounds, including at least one hydrogen bond donor and one hydrogen bond acceptor .^[^ ^20^ ^]^ When the constituents are of natural origin, they are referred to as natural deep eutectic solvents (NaDES).^[^ ^17^ ^]^ The latter generally have the advantage of being biosourced, biodegradable, and nontoxic. These characteristics make them highly desirable for the development of sustainable chemistry. The classification of these solvents has recently become the subject of critical discussion. The names “DES” and “NaDES” require thermodynamic characterization to prove the deep nature of an eutectic system.^[^ ^21^ ^]^ Furthermore, the use of mixtures of compounds only at a ratio corresponding to the eutectic point reduces the range of solvents that can be used. For instance, a solvent composed of betaine and glycerol is liquid and stable at room temperature in proportions ranging from 1:2 to 1:8 (mol mol^−1^).^[^ ^22^ ^]^ For the purposes of this study, we will use the term “eutectic solvent” to denote the mixtures of compounds we will be using as solvents, which have already been described in the literature.

Eutectic solvents have demonstrated their efficacy in a variety of organic and organometallic reactions, ensuring optimal yields and facilitating the recycling of catalysts without compromising their activity.^[^ ^23^, ^24^, ^25^ ^]^ To further enhance their performance, these solvents can be combined with sustainable processes, such as ultrasonics, mechanochemical process, and microwave irradiation (Figure 1).^[^ ^26^, ^27^, ^28^, ^29^, ^30^ ^]^ Our research group has previously investigated the application of an eutectic solvent as a liquid‐assisted grinding for the Suzuki–Miyaura mechanochemical reaction, highlighting a synergistic effect resulting from this combination.^[^ ^31^ ^]^ Although microwave heating is commonly used for cross‐coupling reactions in conventional solvents, it has not yet been reported for this type of reaction using eutectic solvents. However, given their high hydrogen bond content, eutectic solvents are promising candidates for use in microwave‐assisted reactions. To date, there are just a few examples of microwave‐assisted organic synthesis reactions in eutectic solvents. In 2013, Patil et al. presented a pioneering study on the use of an eutectic solvent and microwave heating to enhance an organic reaction, successfully synthesizing nitriles from aldehydes. It was proposed that the DES choline chloride/urea (1:2, mol mol^−1^) played both solvent and catalyst roles in this transformation.^[^ ^32^ ^]^ Subsequent studies of cyclization to form benzoxazoles,^[^ ^33^ ^]^ benzo‐fused *O*‐heterocycles,^[^ ^34^ ^]^ and pentacycles derived from quinazolines^[^ ^35^ ^]^ were carried out under microwave heating, leading to interesting results in terms of yield and reaction time. In 2022, 5‐hydroxymethylfurfural was successfully produced from fructose using a method that involved microwave irradiation and a choline chloride/citric acid (1:1 mol mol^−1^). The reaction time of just 4 min resulted in an 82% yield.^[^ ^36^ ^]^ Some multicomponent reactions have also been described.^[^ ^37^, ^38^, ^39^ ^]^ For example, in 2023, the multicomponent one‐pot synthesis of isoxazol‐5‐ones was carried out in NaDES betaine/citric acid/water (1:1.5:1, mol mol^−1^/mol). Compared with ultrasonic and classical heating conditions, microwave irradiation has been shown to reduce reaction times and improve yields.^[^ ^30^ ^]^ Recently, Molnar et al. compared the performance of using ultrasound, microwaves and mechanochemistry for quaternization reactions in NaDES. Microwaves have been shown to deliver the highest yields and shortest reaction times.^[^ ^26^, ^27^ ^]^

Although the Suzuki–Miyaura coupling reaction has been studied extensively under microwave heating^[^ ^40^, ^41^, ^42^ ^]^ and in various eutectic solvents,^[^ ^25^ ^]^ the combination of these two approaches has not yet been investigated. In this study, we propose to combine the microwave‐assisted Suzuki–Miyaura reaction with eutectic solvents for the synthesis of biphenyl compounds. Reaction conditions were optimized using the design of experiments based on response surface methodology (RSM). To evaluate the sustainability of this methodology, CHEM21 metrics were also performed.^[^ ^43^ ^]^

## 2 Results and Discussion

The optimization of reaction conditions was performed in two stages. First, the most appropriate solvent, catalyst and base were investigated, then other process parameters (amount of catalyst, amount of solvent, temperature, and reaction time) were optimized using a design of experiments.

Based on a method previously developed by our team,^[^ ^31^ ^]^ Suzuki–Miyaura cross‐coupling between bromobenzene **1a** and *p*‐methoxyphenylboronic acid **2a** was performed using palladium acetate in the presence of sodium carbonate as a base in different solvents under microwave irradiation (**Table** **1**). The reaction temperature and time were set at 120 °C and 30 min, respectively. First, the reaction was carried out in water and *N*,*N*‐dimethylformamide (entries 1 and 2), so that the use of eutectic solvents could be compared with solvents commonly used for Suzuki–Miyaura coupling. Several eutectic mixtures based on glycerol, ethylene glycol, choline chloride, urea or betaine were used (entries 3–9). The use of choline chloride resulted in very different NMR yields depending on the associated compound. A very good yield of 83% was obtained in choline chloride/ethylene glycol, whereas in choline chloride/urea, a yield of only 50% was observed (entries 3 and 5). On the other hand, urea combined with glycerol achieves a yield of 75% (entry 6). We were particularly interested in the betaine/glycerol eutectic mixture, which can be used in various proportions from 1:2 to 1:8 (entries 7–9). Note that the smaller the proportion of glycerol, the more viscous the mixture and the more difficult it is to handle. Similar NMR yields of around 72% were obtained for molar ratios 1:2 and 1:4, beyond which increasing the proportion of glycerol results in a slight drop in yield to 64% (entry 9). A similar yield is obtained in glycerol alone (entry 10). In view of these results, the rest of the study will be carried out with the betaine/glycerol mixture (1:4, mol mol^−1^), which seems to offer the best compromise in terms of yield, ease of use and solvent durability.^[^ ^20^ ^]^

**Table 1:** Study of the Suzuki reaction under microwave irradiation in different solvents.a)

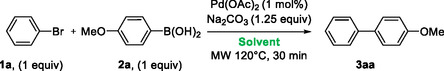		
Entry	Solvent	NMR yield [%]b)
1	Water	69
2	Dimethylformamide	62
3	Choline chloride/ethylene glycol (1:2, mol mol^−1^)	83
4	Choline chloride/glycerol (1:2, mol mol^−1^)	69
5	Choline chloride/urea (1:2, mol mol^−1^)	50
6	Glycerol/urea (4:1, mol mol^−1^)	75
7	Betaine/glycerol (1:2, mol mol^−1^)	72
8	Betaine/glycerol (1:4, mol mol^−1^)	72
9	Betaine/glycerol (1:8, mol mol^−1^)	64
10	Glycerol	64

a) Conditions: **1a** (1 mmol), **2a** (1 mmol), Pd(OAc)_2_ (1 mol%), Na_2_CO_3_ (1.25 mmol), solvent (2.5 g), MW 120 °C for 30 min.

b) Determined by NMR with trimethoxybenzene as internal reference.

Various palladium (II) and palladium (0) catalysts were then tested for the microwave‐assisted coupling of bromobenzene **1a** and *p*‐methoxyphenylboronic acid **2a** in the presence of sodium carbonate as a base, in betaine/glycerol solvent (1:4, mol mol^−1^), at 120 °C for 30 min (**Table** **2**). Compared to the previously obtained conditions (entry 1), the addition of triphenylphosphine as a ligand to palladium acetate resulted in a slight drop in NMR yield (entry 2). Palladium chloride combined with or without triphenylphosphine gave similar yields of around 72% (entries 3 and 4). The 1,1′‐bis(diphenylphosphino)ferrocene ligand improved yields to at least 80% (entries 6 and 7). On the other hand, the use of palladium (0) such as palladium tetrakistriphenylphophine or tris(dibenzylideneacetone)dipalladium led to compound **3aa** in more moderate yields of 49% and 60%, respectively (entries 7 and 8). We have therefore decided to carry out further optimization with [1,1′‐bis(diphenylphosphino)ferrocene]dichloropalladium(II) in complex with dichloromethane, as it achieved the best yield and is slightly less expensive than its dichloromethane‐uncomplexed equivalent.

**Table 2:** Optimization of the catalyst.a)

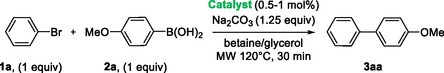		
Entry	Calatyst [mol%]	NMR yield [%]b)
1	Pd(OAc)_2_ (1 mol%)	72
2	Pd(OAc)_2_ (1 mol%)/PPh_3_ (2 mol%)	61
3	PdCl_2_ (1 mol%)	72
4	PdCl_2_(PPh_3_)_2_ (1 mol%)	75
5	PdCl_2_dppf (1 mol%)	80
6	PdCl_2_dppf.CH_2_Cl_2_ (1 mol%)	82
7	Pd(PPh_3_)_4_ (1 mol%)	49
8	Pd_2_dba_3_ (0.5 mol%)	60

a) Conditions: **1a** (1 mmol), **2a** (1 mmol), [Pd] (1 mol%), Na_2_CO_3_ (1.25 mmol), betaine/glycerol (1:4, mol mol^−1^) (2.5 g), MW 120 °C for 30 min.

b) Determined by NMR with trimethoxybenzene as internal reference.

The nature of the base was then considered (**Table** **3**). As demonstrated in entries 1 to 3, sodium, potassium, and cesium carbonates produced NMR yields of 82%, 77%, and 75%, respectively. The use of potassium acetate (entry 4) or triethylamine (entry 5) resulted in lower NMR yields of 68% and 66%, respectively. Sodium carbonate is therefore the most advantageous base, as it is inexpensive and produced the best yield under the conditions studied.

**Table 3:** Optimization of the base.a)

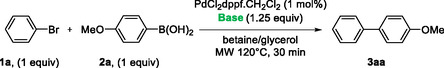		
Entry	Base	NMR yield [%]b)
1	Na_2_CO_3_	82
2	K_2_CO_3_	77
3	Cs_2_CO_3_	75
4	AcOK	68
5	NEt_3_	66

a) Conditions: **1a** (1 mmol), **2a** (1 mmol), PdCl_2_dppf.CH_2_Cl_2_ (1 mol%), Base (1.25 mmol), betaine/glycerol (1:4, mol mol^−1^) (2.5 g), MW 120 °C for 30 min.

b) Determined by NMR with trimethoxybenzene as internal reference.

**Figure 2:**
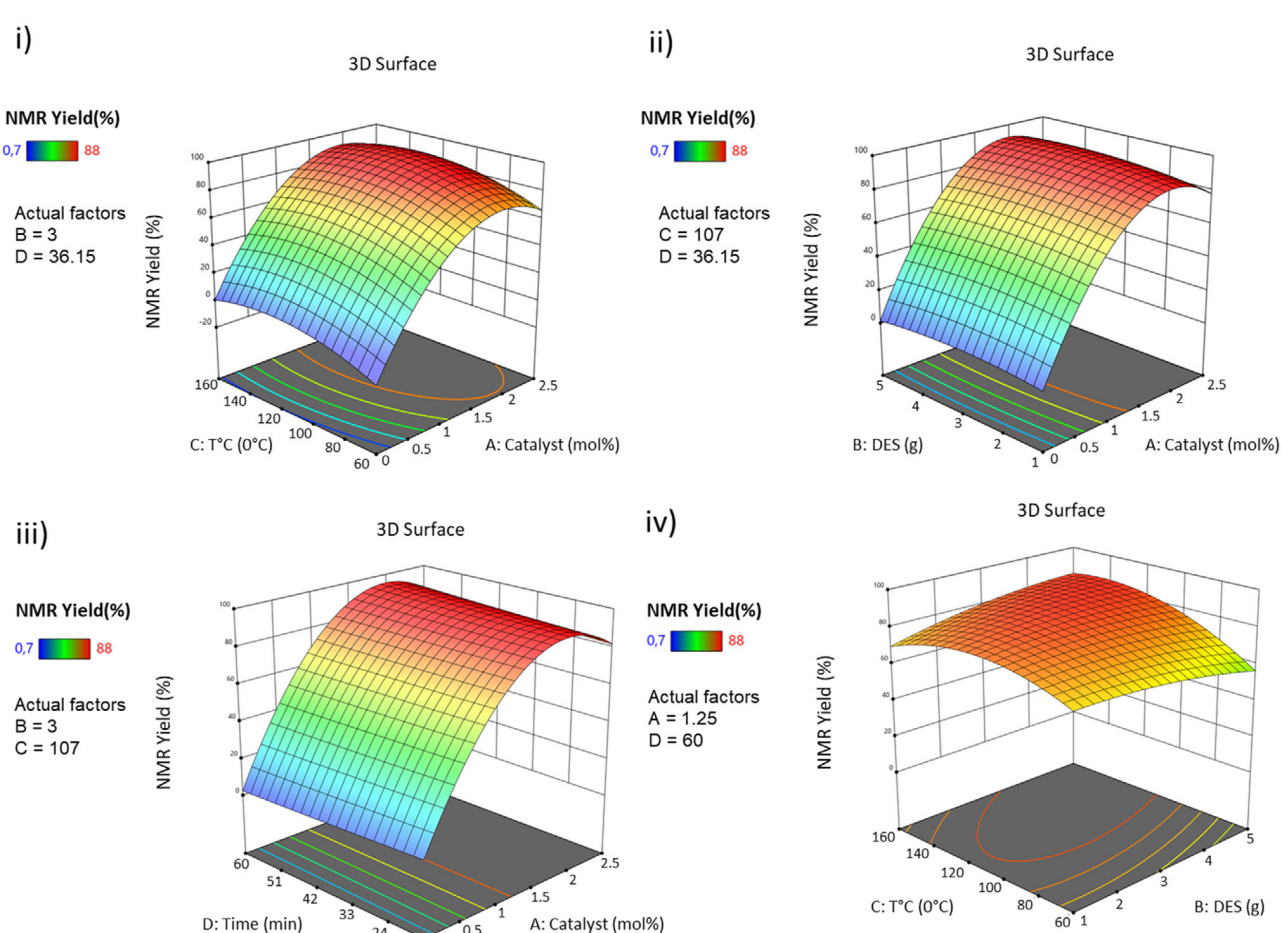
RSM of yield **3aa** NMR using **1a** (1 mmol), **2a** (1 mmol), Na_2_CO_3_ (1.25 mmol), and PdCl_2_dppf.CH_2_Cl_2_ as catalyst, betaine/glycerol (1:4, mol mol^−1^) as solvent, under microwave irradiations with the combined effects. i) Catalyst and temperature, ii) catalyst and solvent iii) catalyst and time; and iv) solvent and temperature.

To optimize catalyst and solvent amounts, temperature and reaction time, a Box–Behnken design of experiments with five center points was performed. All data are summarized in Table S2, Supporting Information and **Figure** **2**. The results obtained after 29 experiments (Table S2, Supporting Information) indicate that the most important parameter is the amount of catalyst, while the other parameters are not very significant. The model is validated by the calculated intercept, which presents a *p*‐value below 0.0001. The adjusted and predicted R^2^ values of the RSM are in good agreement: less than 0.2 difference (Table S3, Supporting Information). The NMR yield could be reliably predicted by the following equation (Equation 1):

(1) $$\text{NMR}\;\text{Yield}=-44.350021265771+87.37073992674\;\text{Catalyst}-1.4744047619048\;\text{Solvent}+\;0.8696054945055\;T\;{}^{\circ}C-0.086115316781979\;\text{Time}+0.58000000000001\;\text{Catalyst}\times \text{Solvent}+\;0.011578021978022\;\text{Catalyst}\times T\;{}^{\circ}C+0.090666666666667\;\text{Catalyst}\times \text{Time}+0.0675\;\text{Solvent}\times T\;{}^{\circ}C-\;0.022222222222222\;\text{Solvent}\times \text{Time}+0.00018852258852257\;T\;{}^{\circ}C\times \text{Time}-24.716249084249{\text{ Catalyst}}^{2}-\;0.82648809523809{\text{ Solvent}}^{2}-0.0043252380952381\;T\;{}^{\circ}C^{2}+0.0003952426174648{\text{ Time}}^{2}$$

Experimental conditions are optimized to maximize NMR yield while minimizing the amount of catalyst, which is a critical parameter in ecological and economic terms. Parameter optimization results in the use of 1.5 mol% catalyst and 2.8 g solvent, with a reaction time of 15 min at 129 °C. These conditions were experimentally applied, and resulted to an NMR yield of 85%, thus confirming the conducted optimization process (±5% deviation from the predicted value).

**Figure 3:**
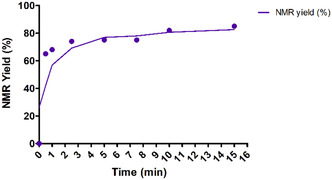
Kinetic study under microwave irradiation.

We then investigated the reaction kinetics (**Figure** **3**). Suzuki–Miyaura coupling was carried out under optimized conditions, with reaction times ranging from 30 s to 15 min. After extraction, NMR yields were determined. The reaction kinetics were very rapid, with an NMR yield of 65% already achieved in 30 s. This was followed by a slight increase in yield to 85% within 15 min. It should be noted that with a conventional heating mode, an NMR yield of only 38% was obtained after 15 min of reaction. As previously described in the literature, these results show that microwave heating in hydrogen bond‐rich, like eutectic solvents media, promotes reactivity and enables short reaction times.^[^ ^26^, ^30^, ^36^ ^]^

**Scheme 1:**
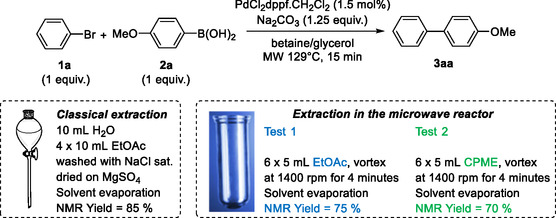
Comparison of extraction processes.

In view of recycling the catalyst into the reaction medium, we investigated a nondestructive method for extracting the eutectic solvent directly into the microwave reactor tube (**Scheme** **1**). After the reaction, 5 mL of ethyl acetate were added to the reaction medium. At this point, the medium became very viscous and stirring was very difficult. The tube was therefore vortexed at 1400 rpm for 4 min. Ethyl acetate, which is immiscible with the reaction medium, could be easily removed. The extraction was performed 6 times, giving an NMR yield of 75%. Following the same protocol, cyclopropylmethylether (CPME), also considered a green solvent,^[^ ^44^ ^]^ was tested. Although it allows better stirring during extraction, an NMR yield of only 70% was obtained. Although lower than the yield obtained by conventional extraction, extraction with ethyl acetate in the reactor will be used for recycling tests.

**Figure 4:**
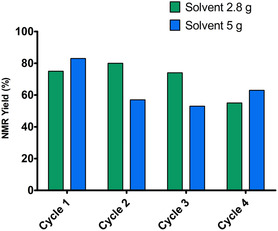
Recycling tests. Cycle 1: **1a** (1 mmol), **2a** (1 mmol), PdCl_2_dppf.CH_2_Cl_2_ (1.5 mol%), Na_2_CO_3_ (1.25 mmol), betaine/glycerol (1:4, mol mol^−1^) (2.8 or 5 g), MW 129 °C for 15 min. Cycles 2, 3, and 4: **1a** (1 mmol), **2a** (1 mmol), Na_2_CO_3_ (1 mmol), MW 129 °C for 15 min.

The recycling capacity of the betaine/glycerol phase containing the catalytic system was then challenged (**Figure** **4**). The first recycling test was carried out under optimized conditions, with ethyl acetate direct extraction into the reactor. From cycle 2 onwards, only reagents **1a** and **2a** and sodium carbonate (1 equivalent) were reintroduced. The catalytic system allowed three cycles without loss of yield. From the fourth cycle onwards, the NMR yield dropped to 55%. After these four cycles, the medium became very viscous and gummy, making agitation and extraction very difficult. This may be due to the accumulation of sodium salts during the cycles. A second recycling trial was carried out with 5 g of solvent instead of 2.8 g. Increasing the solvent volume may allow better agitation during the process. Although the first cycle produced a higher NMR yield of 83%, cycles 2 – 4 produced compound **3aa** in more modest yields of 53% to 63%.

**Scheme 2:**
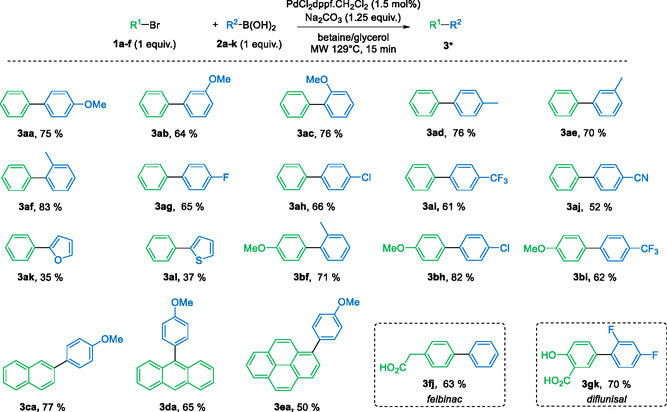
Synthesis of biaryls **3** by Suzuki–Miyaura cross‐coupling in eutectic solvent under microwave irradiations. Conditions: **1** (1 mmol), **2** (1 mmol), PdCl_2_dppf.CH_2_Cl_2_ (1.5 mol%), Na_2_CO_3_ (1.25 mmol), betaine/glycerol (1:4, mol mol^−1^) (2.8 g), MW 129 °C for 15 min. *Isolated yield.

We therefore decided to investigate the scope of our reaction with the optimized conditions (**Scheme** **2**). Regarding the boronic acid partner, the *ortho* and *para* positions were favored, while the *meta* position ended up with the lowest yield, as can be seen from the following series: **3aa**, *p*‐MeO—Ph = 75%, **3ab**, *m*‐MeO—Ph = 64%, **3ac**, *o*‐MeO—Ph = 76% and **3ad**, *p*‐Me—Ph = 76%, **3ae**, *m*‐Me—Ph = 70%, **3af**, *o*‐Me—Ph = 83%. Both electron donating (**3aa**, *p*‐MeO—Ph = 75%, and **3ad**, *p*‐Me—Ph = 76%) and electron withdrawing (**3ag**, *p*‐F—Ph = 65%, **3ah**, *p*‐Cl—Ph = 66%, **3ai**, *p*‐CF_3_—Ph = 61%, and **3aj**, *p*‐CN—Ph = 52%) groups were used with satisfactory results. Boronic acids with heterocyclic groups gave significantly lower yields (**3ak**, furan‐2‐yl = 30%, **3al**, thienyl‐2‐yl = 37%). Other brominated partners were also used instead of bromobenzene. 4‐Bromoanisole gave good yields with different boronic acids (**3bf**, *o*‐Me—Ph = 71%, **3bh**, *p*‐Cl‐Ph = 82%, **3bi**, *p*‐CF_3_‐Ph = 62%). Solid brominated partners were also used with similar yields (**3ca**, naphthalen‐2‐yl = 77%, **3da**, anthracen‐9‐yl = 65%, **3ea**, pyren‐1‐yl = 50%). These brominated compounds are particularly difficult substrates for the Suzuki–Miyaura cross‐coupling reaction. Finally, we decided to apply our conditions to molecules of interest. Felbinac, a nonsteroidal anti‐inflammatory drug, and diflunisal, an analgesic and anti‐inflammatory drug, were synthesized with yields of 63% (**3fj**) and 70% (**3gk**), respectively.

**Scheme 3:**
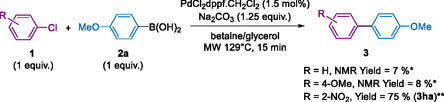
Suzuki–Miyaura cross‐coupling aryl chlorides and **2a** in eutectic solvent under microwave irradiations between. Conditions: **1** (1 mmol), **2** (1 mmol), PdCl_2_dppf.CH_2_Cl_2_ (1.5 mol%), Na_2_CO_3_ (1.25 mmol), betaine/glycerol (1:4, mol mol^−1^) (2.8 g), MW 129 °C for 15 min. *determined by NMR with trimethoxybenzene as internal reference, the product was not isolated. ** Isolated yield.

We then investigated the possibility of performing a Suzuki–Miyaura coupling reaction using aryl chlorides (**Scheme** **3**). The presence and nature of substituents on the aromatic ring strongly influence the reactivity of the aryl chloride. For example, chlorobenzene and 4‐chloroanisol exhibited low reactivity, resulting in the formation of the coupling product with NMR yields below 10%. Conversely, the presence of an electron‐withdrawing nitro group enabled the coupling product **3ha** to be obtained with an isolated yield of 75%. This result is promising for developing this methodology with more diverse substrates.

**Scheme 4:**
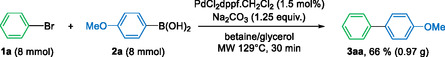
Suzuki–Miyaura cross‐coupling on the gram scale.

The reaction was then carried out under gram‐scale conditions with 8 mmol of bromobenzene **1a** and 8 mmol of *p*‐methoxyboronic acid **2a** in the presence of [1,1′bis(diphenylphosphino)ferrocene]‐dichloropalladium (II) in complex with dichloromethane (1.5 mol%) and sodium carbonate (1.25 equiv.) in betaine/glycerol eutectic solvent (1:4, mol mol^−1^, 20 g) at 129 °C (**Scheme** **4**). A longer time of 30 min was required to obtain the expected compound **3aa** in 66% isolated yield. The main limiting factor was the quality of agitation of the medium. Indeed betaine/glycerol (1:4, mol mol^−1^) is viscous, the increase of the volume media during scale‐up led to inhomogeneous stirring in the glass reaction tube even with paddle stirrers.

**Figure 5:**
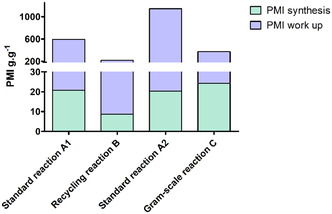
Comparison of PMI of the synthesis of compound **3aa** obtained with different microwave processes: **Microwave standard conditions** **A1**) **1a** (1 mmol), **2a** (1 mmol), PdCl_2_dppf.CH_2_Cl_2_ (1.5 mol%), Na_2_CO_3_ (1.25 mmol), betaine/glycerol (1:4, mol mol^−1^^−^) (2.8 g), MW 129 °C for 15 min/classical extraction/NMR yield (85 %); **Recycling reaction** **B**) Cycle 1: **1a** (1 mmol), **2a** (1 mmol), PdCl_2_dppf.CH_2_Cl_2_ (1.5 mol%), Na_2_CO_3_ (1.25 mmol), betaine/glycerol (1:4, mol mol^−1^) (2.8 or 5 g), MW 129 °C for 15 min. Cycles 2, 3, and 4: **1a** (1 mmol), **2a** (1 mmol), Na_2_CO_3_ (1 mmol), MW 129 °C for 15 min/extraction in the microwave reactor/NMR yield (75%; 80%, 74%, and 55%); **Microwave standard conditions** **A2**) **1a** (1 mmol), **2a** (1 mmol), PdCl_2_dppf.CH_2_Cl_2_ (1.5 mol%), Na_2_CO_3_ (1.25 mmol), betaine/glycerol (1:4, mol mol^−1^) (2.8 g), MW 129 °C for 15 min/classical extraction/isolated yield (75%); **Gram‐scale reaction** **C**) **1a** (8 mmol), **2a** (8 mmol), PdCl_2_dppf.CH_2_Cl_2_ (1.5 mol%), Na_2_CO_3_ (10 mmol), betaine/glycerol (1:4, mol mol^−1^) (20 g), MW 129 °C for 30 min/classical extraction/isolated yield (66%).

Finally, we evaluated our different procedures for the synthesis of compound **3aa** according to CHEM 21 criteria.^[^ ^43^ ^]^ The CHEM21 toolkit enables the sustainability of reactions and their ecological character to be assessed according to quantitative and qualitative criteria. Reaction mass efficiency, atom economy, optimum efficiency, process mass intensity (PMI) and E‐factor were calculated (Table S4, Supporting Information). As PMI is a high‐level measure that can be evaluated for more sustainable processes,^[^ ^45^ ^]^ it will be discussed here for four procedures of microwave‐assisted Suzuki–Miyaura coupling (**Figure** **5**). First, a cycle of microwave‐assisted Suzuki–Miyaura coupling with extraction in a separatory funnel (Figure 5, standard reaction **A1**) and the same reaction with four cycles and extraction in the microwave reactor (Figure 5, recycling reaction **B**) were compared. Although yields are slightly lower, recycling improves the PMI reaction with more than 50% reduction (**B**: 8.7 g g^−1^ vs. **A1**: 20.7 g g^−1^). One‐pot extraction procedure used in the recycling process improves PMI work‐up again allowing a decrease of 50% (**B**: 206.5 g g^−1^ vs. **A1**: 573.2 g g^−1^). In both cases, the work‐up process is the most resource‐intensive step. It is important to note that in this comparison only NMR yields were considered, which contributed to artificially underestimating the resource consumption during the work‐up. We then assessed the suitability for scale‐up. Microwave‐assisted Suzuki–Miyaura coupling procedures on 1 and 8 mmol starting substrate were compared (Figure 5, A2 and C, respectively). In this case, we took into account isolated yields and therefore included purification in the calculation for resource consumption, which explains the considerable increase in PMI especially for condition **A2** (**A2**: 1146.6 g g^−1^). The gram‐scale reaction (**C**) results in a slight increase in the PMI of the reaction (**C**: 24.3 g g^−1^ vs. **A2**: 20.3 g g^−1^), the yield of **3aa** being lower than that of the conventional reaction (66% for 75%). On the other hand, it enables better management of solvent consumption during elaboration (extraction and purification by flash chromatography), resulting in an overall improvement in process PMI. This was confirmed by the PMI work‐up of both processes (Table S4, Supporting Information and Figure 5). Indeed, even though the largest scale used in gram‐scale reaction the PMI process was found to be three times lower than the 1 mmol reaction (**C**: 348.7 g g^−1^ vs. **A2**: 1123 g g^−1^).

## 3 Conclusion

Since the first Suzuki–Miyaura coupling between a boronic acid and an aryl halide in a eutectic solvent described by Konig and co in 2006,^[^ ^46^ ^]^ several studies have been carried out to improve and diversify this methodology.^[^ ^25^ ^]^ The first microwave‐assisted Suzuki–Miyaura coupling in a natural eutectic solvent was described in this work. Compared to traditional heating methods,^[^ ^47^, ^48^, ^49^ ^]^ microwaves increase activity and reduce reaction time allowing a complete reaction within 15 min. A design of experiments revealed optimized synthesis conditions using 1.5 mol% catalyst and 2.8 g solvent, with a reaction time of 15 min at 129 °C. This methodology enabled the synthesis of 20 biphenyls, including two of therapeutic interest, in modest to good yields. Recycling of the betaine/glycerol phase containing the palladium catalyst was carried out three times before a significant decrease in yield was noticed. A scale‐up was also carried out, with a slight loss of activity probably due to nonhomogeneous stirring of the reaction medium due to high viscosity. Evaluation by CHEM21 metrics shows that microwave‐assisted Suzuki–Miyaura coupling in a eutectic solvent aligns with the green chemistry principle, with a dramatic impact on PMI in both synthesis and work‐up steps. The latter remains the more resource‐intensive, and a variety of approaches should be investigated to enhance solvent consumption, including the recycling of the mobile phases from the purification and extraction stage, with the objective of minimizing environmental impact. The use of natural eutectic solvents combined with an eco‐friendly activation method such as mechanochemistry^[^ ^31^ ^]^ and microwave heating has shown a positive impact on the reactivity of the Suzuki–Miyaura reaction. Ultrasonic activation of this reaction in natural eutectic solvents is envisaged to complete this study. This green solvent/eco‐compatible activation combination could be used to reduce the environmental impact of various organic synthesis reactions. This work completes the toolbox of eco‐compatible methodologies for more sustainable chemistry.

## Conflict of Interest

The authors declare no conflict of interest.

## Supporting information

Supplementary Material

## Data Availability

The data that support the findings of this study are available in the supplementary material of this article.

## References

[1] P. T. Anastas , M. M. Kirchhoff , T. C. Williamson , Appl. Catal. A Gen. 2001, 221, 3.

[2] C. Capello , U. Fischer , K. Hungerbühler , Green Chem. 2007, 9, 927.

[3] L. Cicco , G. Dilauro , F. M. Perna , P. Vitale , V. Capriati , Org. Biomol. Chem. 2021, 19, 2558. 33471017 10.1039/d0ob02491k

[4] M. Cortes‐Clerget , J. Yu , J. R. A. Kincaid , P. Walde , F. Gallou , B. H. Lipshutz , Chem. Sci. 2021, 12, 4237. 34163692 10.1039/d0sc06000c PMC8179471

[5] V. Polshettiwar , A. Decottignies , C. Len , A. Fihri , ChemSusChem 2010, 3, 502. 20191633 10.1002/cssc.200900221

[6] N. E. Leadbeater , Chem. Commun. 2005, 2881. 10.1039/b500952a 15957019

[7] D. F. Aycock , Org. Process Res. Dev. 2007, 11, 156.

[8] R. Bijoy , P. Agarwala , L. Roy , B. N. Thorat , Org. Process Res. Dev. 2022, 26, 480.

[9] J. Dupont , B. C. Leal , P. Lozano , A. L. Monteiro , P. Migowski , J. D. Scholten , Chem. Rev. 2024, 124, 5227. 38661578 10.1021/acs.chemrev.3c00379

[10] R. Tomar , P. kundra , J. Sharma , Y. Sangeeta , Catal. Surv. Asia 2024, 28, 311.

[11] M. Scott Padilla , S. Mecozzi , J. Mol. Liq. 2024, 395, 123886.

[12] X. Wang , X. Hu , D. Zhang , Y. Zhang , H. Xu , Y. Sun , X. Gu , J. Luo , B. Gao , J. Environ. Chem. Eng. 2024, 12, 114638.

[13] M. Amde , J. F. Liu , L. Pang , Environ. Sci. Technol. 2015, 49, 12611. 26445034 10.1021/acs.est.5b03123

[14] S. Gracia‐Barberán , A. Leal‐Duaso , E. Pires , Curr. Opin. Green Sustain Chem. 2022, 35, 100610.

[15] V. Zullo , A. Iuliano , L. Guazzelli , Molecules 2021, 26, 2052. 33916695 10.3390/molecules26072052 PMC8038380

[16] E. L. Smith , A. P. Abbott , K. S. Ryder , Chem. Rev. 2014, 114, 11060. 25300631 10.1021/cr300162p

[17] A. Paiva , R. Craveiro , I. Aroso , M. Martins , R. L. Reis , A. R. C. Duarte , ACS Sustain. Chem. Eng. 2014, 2, 1063.

[18] Y. Liu , J. B. Friesen , J. B. McAlpine , D. C. Lankin , S.‐N. Chen , G. F. Pauli , J. Nat. Prod. 2018, 81, 679. 29513526 10.1021/acs.jnatprod.7b00945 PMC5913660

[19] D. A. Alonso , A. Baeza , R. Chinchilla , G. Guillena , I. M. Pastor , D. J. Ramón , Eur. J. Org. Chem. 2016, 2016, 612.

[20] D. O. Abranches , J. A. P. Coutinho , Annu. Rev. Chem. Biomol. Eng. 2023, 14, 141. 36888992 10.1146/annurev-chembioeng-101121-085323

[21] B. B. Hansen , S. Spittle , B. Chen , D. Poe , Y. Zhang , J. M. Klein , A. Horton , L. Adhikari , T. Zelovich , B. W. Doherty , B. Gurkan , E. J. Maginn , A. Ragauskas , M. Dadmun , T. A. Zawodzinski , G. A. Baker , M. E. Tuckerman , R. F. Savinell , J. R. Sangoro , Chem. Rev. 2021, 121, 1232. 33315380 10.1021/acs.chemrev.0c00385

[22] L. Meredith , A. Elbourne , T. L. Greaves , G. Bryant , S. J. Bryant , J. Mol. Liq. 2024, 394, 123777.

[23] L. Peng , Z. Hu , Q. Lu , Z. Tang , Y. Jiao , X. Xu , Chinese Chem. Lett. 2019, 30, 2151.

[24] S. E. Hooshmand , R. Afshari , D. J. Ramón , R. S. Varma , Green Chem. 2020, 22, 3668.

[25] S. Pasricha , P. Gahlot , T. M. Rangarajan , Shikha , Deepak , H. Pahuja , D. Yadav , Pratham , K. Pilania , S. Anand , J. Mol. Liq. 2025, 426, 127287.

[26] V. Bušić , S. Roca , D. Vikić‐Topić , K. Vrandečić , J. Ćosić , M. Molnar , D. Gašo‐Sokač , Environ. Chem. Lett. 2020, 18, 889.

[27] V. Bušić , M. Molnar , V. Tomičić , D. Božanović , I. Jerković , D. Gašo‐Sokač , Molecules 2022, 27, 7429. 36364264 10.3390/molecules27217429 PMC9655353

[28] M. Komar , T. G. Kraljević , I. Jerković , M. Molnar , Molecules 2022, 27, 558. 35056873 10.3390/molecules27020558 PMC8780518

[29] M. Komar , V. Rastija , D. Bešlo , M. Molnar , J. Mol. Struct. 2024, 1304, 137725.

[30] A. Zadem , Z. Cheraiet , B.‐H. Chahra , Polycycl. Aromat. Compd. 2024, 44, 5188.

[31] E. Thiery , P. Delaye , J. Thibonnet , L. Boudesocque‐Delaye , Eur. J. Org. Chem. 2023, 26, e202300727.

[32] U. Patil , S. Shendage , J. Nagarkar , Synthesis 2013, 45, 3295.

[33] P. T. Pham , H. T. Nguyen , T. T. Nguyen , L. H. T. Nguyen , M.‐H. D. Dang , T. L. H. Doan , D. D. Pham , C. T. Nguyen , P. H. Tran , Catalysts 2022, 12, 1394.

[34] C. Teja , A. Garg , G. K. Rohith , H. Roshini , S. Jena , F. R. Nawaz Khan , Polycycl. Aromat. Compd. 2022, 42, 4769.

[35] C.‐T. Ma , P. Liu , W. Wu , Z.‐H. Zhang , J. Mol. Liq. 2017, 242, 606.

[36] E. S. Morais , M. G. Freire , C. S. R. Freire , A. J. D. Silvestre , Int. J. Mol. Sci. 2022, 23, 1959. 35216072 10.3390/ijms23041959 PMC8875992

[37] M. Zhang , P. Liu , Y.‐H. Liu , Z.‐R. Shang , H.‐C. Hu , Z.‐H. Zhang , RSC Adv. 2016, 6, 106160.

[38] A. Castro , I. M. G. Andrade , M. C. Coelho , D. P. da Costa , D. das N. Moreira , R. A. Maia , G. da S. Lima , G. F. dos Santos , B. G. Vaz , G. C. G. Militão , P. B. N. da Silva , M. L. A. de A. Vasconcellos , C. G. Lima‐Junior , J. Heterocycl. Chem. 2023, 60, 392.

[39] M. Shaikh , M. Shaikh , D. Wagare , A. Ahmed Sheikh , S. Sultan Kasim , Curr. Catal. 2022, 11, 65.

[40] K. Martina , M. Manzoli , E. C. Gaudino , G. Cravotto , Catalysts 2017, 7, 98.

[41] K. S. M. Salih , Y. Baqi , Catalysts 2020, 10, 4.

[42] M. Rahman , S. Ghosh , D. Bhattacherjee , G. V. Zyryanov , A. Kumar Bagdi , A. Hajra , Asian J. Org. Chem. 2022, 11, e202200179.

[43] C. R. McElroy , A. Constantinou , L. C. Jones , L. Summerton , J. H. Clark , Green Chem. 2015, 17, 3111.

[44] G. de Gonzalo , A. R. Alcántara , P. Domínguez de María , ChemSusChem 2019, 12, 2083. 30735610 10.1002/cssc.201900079

[45] C. Jimenez‐Gonzalez , C. S. Ponder , Q. B. Broxterman , J. B. Manley , Org. Process Res. Dev. 2011, 15, 912.

[46] G. Imperato , S. Höger , D. Lenoir , B. König , Green Chem. 2006, 8, 1051.

[47] X. Marset , A. Khoshnood , L. Sotorríos , E. Gómez‐Bengoa , D. A. Alonso , D. J. Ramón , ChemCatChem 2017, 9, 1269.

[48] M. Niakan , M. Masteri‐Farahani , S. Karimi , H. Shekaari , J. Mol. Liq. 2021, 324, 115078. 10.1016/j.carbpol.2020.117109 33142646

[49] A. Monteiro , Z. Hussain , T. Eichler , B. Leal , J. Braz. Chem. Soc. 2024, e‐20230197.

