# Supplementary material for: Simple and Rapid Microwave‐Assisted Suzuki–Miyaura Cross‐coupling in Betaine/Glycerol Natural Eutectic Solvent
Source: ChemistryOpen. 2025 Jun 17;14(11):e202500138. doi: 10.1002/open.202500138 (PMC12598815; doi:10.1002/open.202500138)
Supplement: Supplementary file 1 — Supplementary Material [file OPEN-14-e202500138-s001.pdf]

Microwaves Assisted Suzuki-Miyaura Cross-Coupling in Natural Eutectic Solvent

Chefikou Salami,[a] Leslie Boudesocque-Delaye[a] Pierre-Olivier Delaye,*[a] and Emilie Thiery*[a]

Table of contents

1. Materials and Methods 2

2. Eutectic solvents synthesis 2

3. Process optimization using the design of experiment approach 3

4. General procedure for the microwaves Suzuki-Miyaura coupling 4

5. Procedure for the microwaves Suzuki-Miyaura coupling on the gram scale 5

6. Typical metrics applied at First Pass according to the CHEM21 5

7. Analytical data for the compounds **3** 8

8. ^{1}H and ^{13}C spectrum of compounds **3** 12

9. References 37

1. Materials and Methods

All microwave-assisted reactions were carried out in borosilicate glass and silicon carbide reactors using an Anton Paar Monowaves 450.

Reactions were monitored by TLC with Merck® Silica gel 60 F254. Purifications by flash chromatography were carried out using Merck® Geduran® Si 60 silica gel (40-63 µm).

NMR spectra were recorded on a Bruker® Avance 300 (300 MHz) NMR spectrometer using $CDCl_3$ or DMSO-*d6* as solvent. ^1H NMR data, reported using $CHCl_3$ (δ_H = 7.26 ppm) or DMSO-*d5* (δ_H = 2.50 ppm) as internal reference, were as follows (in order): chemical shift (δ in ppm relative to $CHCl_3$), multiplicity (s, d, t, q, quint, m, br for singlet, doublet, triplet, quartet, quintuplet, multiplet, broad) and coupling constants (*J* in Hz). ^{13}C NMR was recorded at 75 MHz on the same instrument, using the $CDCl_3$ solvent peak at (δ_C = 77.16 ppm) or DMSO-d6 solvent peak at (δ_C = 39.52 ppm) as reference. ^{19}F NMR was recorded at 282 MHz on the same instrument. NMR yields were determined in DMSO-*d6* with trimethoxybenzene as internal reference.

The mass spectra of synthesized compounds were recorded on Masse spectrometer SQD ESI Waters in infusion mode at 20 µL (source temperature 150 °C, probe temperature 250°C; APCI+ mode (Corona 15 µA, Cone voltage 35 V or 20 V, extractor 3V), aquisition during 1 min (1s per scan, m/z 100 to 400).

2. Eutectic solvents synthesis

In a flask fitted with a magnetic stirrer, the solvent components are added in the correct molar ratio. The heterogeneous mixture is stirred at 80 °C for 1 hour. The eutectic solvent thus formed, is then brought back and stored at room temperature.

Table S1. Composition of eutectic solvents used in this study.

Component 1	Component 2	Molar ratio
Choline chloride	Ethylene glycol	1 :2
Choline chloride	Glycerol	1 :2
Choline chloride	Urea	1 :2
Glycerol	Urea	4 :1
Bétaïne	Glycerol	1 :2
Bétaïne	Glycerol	1 :4
Bétaïne	Glycerol	1 :6
Bétaïne	Glycerol	1 :8

3. Process optimization using the design of experiment approach

Box-behken design was adopted for parameter optimization of microwaves assisted Suzuki-Miyaura reaction. The parameter ranges were fixed based on conducted preliminary experiments: amount of catalyst (0-2.5 mol%), amount of solvent (1-5 g), temperature (60-160°C) and reaction time (15-60 min). A total of 29 experiments were conducted in this study to optimize reaction parameters. The analysis of variance (ANOVA) was conducted for the validation of theoretical accounts of the optimization process.

The analyses were conducted using Design-Expert 13 (Stat-Ease, Minneapolis, MN, USA). The model adequacy was assessed based on the obtained coefficient of multiple determination (R^2), coefficient of variance (CV) and *p*-values for the model and lack of fit testing.

Table S2. Experimental plan used for the optimization of microwaves assisted Suzuki-Miyaura coupling.

		Factor 1	Factor 2	Factor 3	Factor 4	Response 1
Std	Run	A:Catalyst	B:Solvent	C:Temp	D:Time	NMR Yield
		mol%	g	°C	min	%
29	1	1,25	3	110	37,5	79
21	2	1,25	1	110	15	74
11	3	0	3	110	60	0,8
2	4	2,5	1	110	37,5	80
25	5	1,25	3	110	37,5	83
22	6	1,25	5	110	15	82
15	7	1,25	1	160	37,5	70
4	8	2,5	5	110	37,5	86
28	9	1,25	3	110	37,5	80
20	10	2,5	3	160	37,5	81
1	11	0	1	110	37,5	0,7
14	12	1,25	5	60	37,5	56
12	13	2,5	3	110	60	85
7	14	1,25	3	60	60	68
27	15	1,25	3	110	37,5	88
8	16	1,25	3	160	60	81
6	17	1,25	3	160	15	78
3	18	0	5	110	37,5	0,9
18	19	2,5	3	60	37,5	65
26	20	1,25	3	110	37,5	88
9	21	0	3	110	15	11
10	22	2,5	3	110	15	85
16	23	1,25	5	160	37,5	85
19	24	0	3	160	37,5	0,9
17	*25*	*0*	*3*	*60*	*37,5*	~~*0,5*~~
5	*26*	*1,25*	*3*	*60*	*15*	~~*54*~~
13	27	1,25	1	60	37,5	68
23	28	1,25	1	110	60	80
24	29	1,25	5	110	60	84

These rows were ignored for statistical analysis: 26, 25.

Table S3. Statistics for microwaves assisted Suzuki-Miyaura reaction DoE

ANOVA for Quadratic model

**Source**	**Sum of Squares**	**df**	**Mean Square**	**F-value**	**p-value**	
**Model**	24665,37	14	1761,81	144,43	< 0.0001	significant
A-Catalyst	15734,34	1	15734,34	1289,88	< 0.0001	
B-DES	37,45	1	37,45	3,07	0,1052	
C-T°C	385,47	1	385,47	31,60	0,0001	
D-Time	0,5952	1	0,5952	0,0488	0,8289	
AB	8,41	1	8,41	0,6894	0,4226	
AC	1,26	1	1,26	0,1035	0,7532	
AD	26,01	1	26,01	2,13	0,1699	
BC	182,25	1	182,25	14,94	0,0022	
BD	4,00	1	4,00	0,3279	0,5775	
CD	0,1084	1	0,1084	0,0089	0,9264	
A^2	8693,09	1	8693,09	712,65	< 0.0001	
B^2	65,81	1	65,81	5,40	0,0386	
C^2	579,44	1	579,44	47,50	< 0.0001	
D^2	0,2334	1	0,2334	0,0191	0,8923	
**Residual**	146,38	12	12,20			
Lack of Fit	73,18	8	9,15	0,4999	0,8126	not significant
Pure Error	73,20	4	18,30			
**Cor Total**	24811,75	26				

**Std. Dev.**	3,49	**R^2**	0,9941
**Mean**	64,46	**Adjusted R^2**	0,9872
**C.V. %**	5,42	**Predicted R^2**	0,9729
		**Adeq Precision**	33,5783

4. General procedure for the microwaves-assisted Suzuki-Miyaura coupling

Betaïne/glycerol (1:4, mol/mol) (2.8 g), boronic acid (1 mmol), sodium carbonate (1.25 mmol, 132.5 mg), [1,1′-bis(diphenylphosphino)ferrocene]dichloropalladium(II) in complex with dichloromethane (0.015 mmol, 12.2 mg) and aryl halogenated (1 mmol) were introduced in a microwaves reactor (30 mL). The reaction mixture was irradiated in microwave oven at 129°C for 15 minutes, cooled at room temperature, diluted with EtOAc (10 mL) and water (10 mL). The aqueous phase was extracted with EtOAc (3 x 10 mL). The organic phases were washed with brine (40 mL), dried over anhydrous $MgSO_4$, filtered and solvents were evaporated under vacuum. The residue was purified by column chromatography on silica gel with cyclohexane / EtOAc or cyclohexane / CH_2Cl_2 or CH_2Cl_2 / EtOH as eluent to afford the expected compound.

5. Procedure for the microwaves Suzuki-Miyaura coupling on the gram scale

Betaïne/glycerol (1:4, mol/mol) (20 g), *p*-methoxyphénylboronic acid (8 mmol, 1.22 g), sodium carbonate (10 mmol, 1.06 mg), [1,1′-bis(diphenylphosphino)ferrocene]dichloropalladium(II) in complex with dichloromethane (0.12 mmol, 98.0 mg) and bromobenzene (8 mmol, 1.25 g) were introduced in a microwaves reactor (30 mL). The reaction mixture was irradiated in microwave oven at 129°C for 30 minutes, cooled at room temperature, diluted with EtOAc (15 mL) and water (15 mL). The aqueous phase was extracted with EtOAc (3 x 15 mL). The organic phases were washed with brine (40 mL), dried over anhydrous $MgSO_4$, filtered and solvents were evaporated under vacuum. The residue was purified by column chromatography on silica gel with cyclohexane / EtOAc as eluent to afford the expected compound.

6. Typical metrics applied at First Pass according to the CHEM21[43]

Quantitative Metrics and E-factor calculation for the synthesis of **4-Methoxy-1,1'-biphenyl** (**3aa**) according to our process assisted by microwaves.

*6.1. Microwaves standard conditions **A1***

Conditions: **1a** (1 mmol), **2a** (1 mmol), $PdCl_2dppf.CH_2Cl_2$ (1.5 mol%), Na_2CO_3 (1.25 mmol), Betaine/glycerol (1 :4, mol/mol) (2.8 g), MW 129°C for 15 minutes/classical extraction/NMR yield (85 %).

mass of product (**3aa**) = 0.157 g

mass reactants = 0.156 g (**1a**) + 0.152 g (**2a**) = 0.308 g

Reaction mass efficiency (RME) = mass of product/mass reagents × 100 = 0.157/ 0.308 g ×100 = **51.0**

MW product **3aa** = 184.24 g/mol

MW reagents = 155.96 (**1a**) + 151.96 (**2a**) = 307.92 g/mol

Atom economy (AE) = MW product/MW reagents × 100 = 184.24/307.92 ×100 = **59.7**

Optimum efficiency (OE) = RME/AE×100 = 51.0/59.7 ×100 = **85.3**

Process mass intensity (PMI) = [0.156 g (**1a**) + 0.152 g (**2a**) + 2.8 g (solvent) + 0.132 g (Na_2CO_3) + 0.012 g ([Pd]) + 36 g (EtOAc) + 10 g (H_2O) + 44 g (brine)]/0.157 g = **594.0 g g-1**

Total amount of reactants, reagents, catalyst, solvent: 0.156 g (**1a**) + 0.152 g (**2a**) + 2.8 g (solvent) + 0.132 g (Na_2CO_3) + 0.012 g ([Pd]) + 36 g (EtOAc) + 10 g (H_2O) + 44 g (brine) = 93.252 g

Amount of waste: 93.252 – 0.157 g = 93.095 g

E-factor = amount of waste/amount of product = 93.107/0.157 g = **593.0**

*6.2. Recycling reaction **B***

Conditions: Cycle 1: **1a** (1 mmol), **2a** (1 mmol), $PdCl_2dppf.CH_2Cl_2$ (1.5 mol%), Na_2CO_3 (1.25 mmol), Betaine/glycerol (1 :4, mol/mol) (2.8 or 5 g), MW 129°C for 15 minutes. Cycles 2, 3, 4: **1a** (1 mmol), **2a** (1 mmol), Na_2CO_3 (1 mmol), MW 129°C for 15 minutes/extraction in the microwaves reactor/NMR yield (75 %, 80%, 74%, 55%).

mass of product (**3aa**) = 184.24 x (0.75 + 0.8 + 0.74 + 0.55) = 0.523 g

mass reactants = 0.156 g x 4 (**1a**) + 0.152 g x 4 (**2a**) = 1.232 g

Reaction mass efficiency (RME) = mass of product/mass reagents × 100 = 0.523/ 1.232 g ×100 = **42.5**

MW product **3aa** = 184.24 g/mol

MW reagents = 155.96 (**1a**) + 151.96 (**2a**) = 307.92 g/mol

Atom economy (AE) = MW product/MW reagents × 100 = 184.24/307.92 ×100 = **59.7**

Optimum efficiency (OE) = RME/AE×100 = 42.5/59.7 ×100 = **71.1**

Process mass intensity (PMI) = [0.156 g x 4 (**1a**) + 0.152 g x 4 (**2a**) + 2.8 g (solvent) + 0.132 g x 4 (Na_2CO_3) + 0.012 g ([Pd]) + 108 g (EtOAc)]/0.523 g = **215.2 g g-1**

Total amount of reactants, reagents, catalyst, solvent: 0.156 g x 4 (**1a**) + 0.152 g x 4 (**2a**) + 2.8 g (solvent) + 0.132 g x 4 (Na_2CO_3) + 0.012 g ([Pd]) + 108 g (EtOAc) = 112.572 g
Amount of waste: 112.572 – 0.523 g = 112.049 g
E-factor = amount of waste/amount of product = 112.049/0.523 g = **214.2**

6.3. Microwaves standard conditions ***A2***
Conditions: **1a** (1 mmol), **2a** (1 mmol), $PdCl_2dppf.CH_2Cl_2$ (1.5 mol%), Na_2CO_3 (1.25 mmol), Betaine/glycerol (1 :4, mol/mol) (2.8 g), MW 129°C for 15 minutes/classical extraction/isolated yield (75 %).
mass of product (**3aa**) = 0.138 g
mass reactants = 0.156 g (**1a**) + 0.152 g (**2a**) = 0.308 g
Reaction mass efficiency (RME) = mass of product/mass reagents × 100 = 0.138/ 0.308 g ×100 = **44.8**
MW product **3aa** = 184.24 g/mol
MW reagents = 155.96 (**1a**) + 151.96 (**2a**) = 307.92 g/mol
Atom economy (AE) = MW product/MW reagents × 100 = 184.24/307.92 ×100 = **59.7**
Optimum efficiency (OE) = RME/AE×100 = 44.8/59.7 ×100 = **75.0**
Process mass intensity (PMI) = [0.156 g (**1a**) + 0.152 g (**2a**) + 2.8 g (solvent) + 0.132 g (Na_2CO_3) + 0.012 g ([Pd]) + 36 g (EtOAc) + 10 g (H_2O) + 44 g (brine) + 1.5 g (MgSO4) + 12 g (Silice) + 51.48 (Cyclohexane)]/0.138 g = **1146.6 g g-1**
Total amount of reactants, reagents, catalyst, solvent: 0.156 g (**1a**) + 0.152 g (**2a**) + 2.8 g (solvent) + 0.132 g (Na_2CO_3) + 0.012 g ([Pd]) + 36 g (EtOAc) + 10 g (H_2O) + 44 g (brine) + 1.5 g (MgSO4) + 12 g (Silice) + 51.48 (Cyclohexane) = 158.232 g
Amount of waste: 93.252 – 0.138 g = 158.094 g
E-factor = amount of waste/amount of product = 158.094 g/0.138 g = **1145.6**

6.4. Gram-scale reaction ***C***
Conditions: **1a** (8 mmol), **2a** (8 mmol), $PdCl_2dppf.CH_2Cl_2$ (1.5 mol%), Na_2CO_3 (10 mmol), Betaine/glycerol (1 :4, mol/mol) (20 g), MW 129°C for 30 minutes/classical extraction/isolated yield (66 %).
mass of product (**3aa**) = 0.972 g
mass reactants = 1.248 g (**1a**) + 1.216 g (**2a**) = 2.464 g
Reaction mass efficiency (RME) = mass of product/mass reagents × 100 = 0.138/ 0.308 g ×100 = **39.4**
MW product **3aa** = 184.24 g/mol
MW reagents = 155.96 (**1a**) + 151.96 (**2a**) = 307.92 g/mol
Atom economy (AE) = MW product/MW reagents × 100 = 184.24/307.92 ×100 = **59.7**
Optimum efficiency (OE) = RME/AE×100 = 39.4/59.7 ×100 = **66.0**
Process mass intensity (PMI) = [1.248 g (**1a**) + 1.216 g (**2a**) + 20 g (solvent) + 1.06 g (Na_2CO_3) + 0.096 g ([Pd]) + 54 g (EtOAc) + 15 g (H_2O) + 44 g (brine) + 2.0 g (MgSO4) + 25 g (Silice) + 198.9 (Cyclohexane)]/0.972 g = **373.0 g g-1**
Total amount of reactants, reagents, catalyst, solvent: 1.248 g (**1a**) + 1.216 g (**2a**) + 20 g (solvent) + 1.06 g (Na_2CO_3) + 0.096 g ([Pd]) + 54 g (EtOAc) + 15 g (H_2O) + 44 g (brine) + 2.0 g (MgSO4) + 25 g (Silice) + 198.9 (Cyclohexane) = 362.52 g
Amount of waste: 362.52 – 0.972 g = 361.548 g
E-factor = amount of waste/amount of product = 361.548 g/0.972 g = **371.96**

Table S4. Comparison of metric for different microwaves processes

Metrics	Standard reaction A1[a]	Recycling reaction B[a]	Standard reaction A2[b]	Gram-scale reaction C[b]
mass of product (**3aa**)	157 mg	523 mg	138 mg	0.972 g
mass reactants	308 mg	1.232 g	308 mg	2.464 g
RME	**51.0**	**42.5**	**44.8**	**39.4**
MW product **3aa**	284.36 g/mol	284.36 g/mol	284.36 g/mol	284.36 g/mol
MW reactants	307.92 g/mol	307.92 g/mol	307.92 g/mol	307.92 g/mol
AE	**59.7**	**59.7**	**59.7**	**59.8**
OE	**85.3**	**71.1**	**75.0**	**66.0**
PMI	**594.0**	**215.2**	**1146.6**	**373.0**
PMI reaction	**20.7**	**8.7**	**20.3**	**24.3**
PMI work-up	**573.2**	**206.5**	**1123.0**	**348.7**
Total amount of reagents	93.252 g	112.572 g	158.232 g	362.52
Amount of waste	93.095 g	112.049 g	158.094 g	361.548
E-factor	**593.0**	**214.2**	**1145.6**	**371.96**
Solvent	(EtOAC)		(EtOAC)<br>(Cyclohexane)	
Catalyst	(1.5 mol%)			
Stoichiometric reagent	(Stoichiometric)			
Critical elements	(Pd)			
Energy	(between 70 to 140°C)			
Work-up	(extraction)		(extraction)<br>(flash chromatogrphy)	
Catalyst recycling		(4 cycles)		

[a] MNR Yield. [b] Isolated Yield.

7. Analytical data for the compounds 3

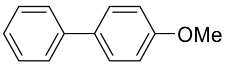

4-Methoxy-1,1'-biphenyl (3aa):[50]$C_{13}H_{12}O$, MW = 184.24 g/mol, yield = 79 %, white solid. **^{1}H NMR** (300 MHz, $CDCl_3$) δ = 7.57-7-51 (m, 4H), 7.45-7.39 (m, 2H), 7.33-7.28 (m, 1H), 7.01-6.96 (m, 1H), 3.86 (s, 3H). **^{13}C NMR** (75 MHz, $CDCl_3$) δ = 159.2 (C), 140.9 (C), 133.8 (C), 128.8 (2CH), 128.2 (2CH), 126.8 (2CH), 126.8 (CH), 114.3 (2CH), 55.4 (CH_3). **MS** (APCI+) m/z: $[M+H]^+$ calcd. for $C_{13}H_{13}O$ is 185.10, found 185.06.

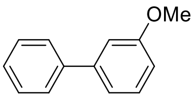

3-Methoxy-1,1'-biphenyl (3ab):[51] $C_{13}H_{12}O$, MW = 184.24 g/mol, yield = 63 %, white solid. **^{1}H NMR** (300 MHz, $CDCl_3$) δ = 7.61-7.57 (m, 2H), 7.47-7.42 (m, 2H), 7.39-7.33 (m, 2H), 7.19 (ddd, *J* = 7.6, 1.6, 1.0 Hz, 1H), 7.14-7.13 (m, 1H), 6.91 (ddd, *J* = 8.2, 2.6 Hz, 0.9 Hz, 1H), 3.87 (s, 3H). **^{13}C NMR** (75 MHz, $CDCl_3$) δ = 160.0 (C), 142.9 (C), 141.2 (C), 129.9 (CH), 128.9 (2CH), 127.5 (CH), 127.3 (2CH), 119.8 (CH), 113.0 (CH), 112,8 (CH), 55.4 (CH_3). **MS** (APCI+) m/z: $[M+H]^+$ calcd. for $C_{13}H_{13}O$ is 185.10, found 185.14.

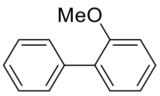

2-Methoxy-1,1'-biphenyl (3ac):[51] $C_{13}H_{12}O$, MW = 184.24 g/mol, yield = 76 %, yellow liquid. **^{1}H NMR** (300 MHz, $CDCl_3$) δ = 7.63-7.59 (m, 2H), 7.50-7.44 (m, 2H), 7.41-7.34 (m, 3H), 7.09 (td, *J* = 7.5 Hz, 1. Hz, 1H), 7.02 (d, *J* = 8.1 Hz, 1H), 3.82 (s, 3H), **^{13}C NMR** (75 MHz, $CDCl_3$) δ = 156.5 (C), 138.6 (C), 131.0 (CH), 130.9 (CH), 130.7 (C), 129.6 (2CH), 128.7 (CH), 128.0 (2CH), 127.0 (CH), 120.9 (CH), 111,2 (CH), 55.5 (CH_3). **MS** (APCI+) m/z: $[M^{+\cdot}]$ calcd. for $C_{13}H_{12}O$ is 184.09, found 184,87.

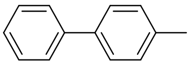

4-Methyl-1,1'-biphenyl (3ad):[51] $C_{13}H_{12}$, MW = 168.24 g/mol, yield = 76 %, white solid. **^{1}H NMR** (300 MHz, $CDCl_3$) δ = 7.62-7.59 (m, 2H), 7.53-7.50 (m, 2H), 7.48-7.42 (m, 2H), 7.38-7.32 (m, 1H), 7.29-7.26 (m, 2H), 2.42 (s, 3H). **^{13}C NMR** (75 MHz, $CDCl_3$) δ = 141.3 (C), 138.6 (C), 137.1 (C), 129.6 (2CH), 128.8 (2CH), 127.1 (5CH), 21.2 (CH_3). **MS** (APCI+) m/z: $[M^{+\cdot}]$ calcd. for $C_{13}H_{12}$ is 168.09, found 168.15.

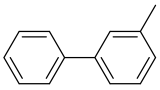

3-Methyl-1,1'-biphenyl (3ae):[52] $C_{13}H_{12}$, MW = 168.24 g/mol, yield = 70 %, colorless liquid. **^{1}H NMR** (300 MHz, $CDCl_3$) δ = 7.65-7.61 (m, 2H), 7.50-7.43 (m, 4H), 7.40-7.35 (m, 2H), 7.21 (d, *J* = 7.2 Hz, 1H), 2.46 (s, 3H). **^{13}C NMR** (75 MHz, $CDCl_3$) δ = 141.4 (C), 141.3 (C), 138.4 (C), 128.81 (2CH), 128.78 (CH), 128.11 (CH), 128.08 (CH), 127.3 (3CH), 124.4 (1CH), 21.7 (CH_3). **MS** (APCI+) m/z: $[M^{+\cdot}]$ calcd. for $C_{13}H_{12}$ is 168.09, found 168.14.

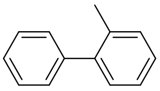

2-Methyl-1,1'-biphenyl (3af):[51] $C_{13}H_{12}$, MW = 168.24 g/mol, yield = 83 %, colorless liquid. **^{1}H NMR** (300 MHz, $CDCl_3$) δ = 7.46-7.40 (m, 2H), 7.38-7.32 (m, 3H), 7.30-7.23 (m, 4H), 2.88 (s, 3H). **^{13}C NMR** (75 MHz, $CDCl_3$) δ = 142.1 (C), 142.0 (C), 135.4 (C), 130.4 (CH), 129.9 (CH),

129.3 (2CH), 128.2 (2CH), 127.4 (CH), 126.9 (CH), 125.9 (CH), 20.6 (CH_3). **MS** (APCI+) m/z: $[M^{+\cdot}]$ calcd. for $C_{13}H_{12}$ is 168.09, found 168.11.

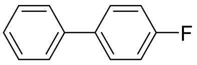

4-Fluoro-1,1'-biphenyl (3ag):[51] $C_{12}H_9F$, MW = 172.20 g/mol, yield = 66 %, white solid. **^{1}H NMR** (300 MHz, $CDCl_3$) δ = 7.58-7.51 (m, 4H), 7.47-7.41 (m, 2H), 7.37-7.32 (m, 1H), 7.17-7.09 (m, 2H). **^{19}F NMR** (282 MHz, $CDCl_3$) δ = -115.9. **^{13}C NMR** (75 MHz, $CDCl_3$) δ = 162.6 (d, *J* = 245 Hz, CF), 140.3 (C), 137.4 (d, *J* = 3 Hz, C), 128.9 (2CH), 128.9 (d, *J* = 8 Hz, 2CH), 127.4 (CH), 127.1 (2CH), 115.7 (d, *J* = 21 Hz, 2CH). **MS** (APCI+) m/z: $[M^{+\cdot}]$ calcd. for $C_{12}H_9F$ is 172.07, found 172.07.

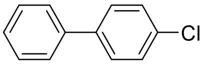

4-Chloro-1,1'-biphenyl (3ah):[51] $C_{12}H_9Cl$, MW = 188.65 g/mol, yield = 65 %, white solid. **^{1}H NMR** (300 MHz, $CDCl_3$) δ = 7.42-7.33 (m, 4H), 7.32-7.18 (m, 5H). **^{13}C NMR** (75 MHz, $CDCl_3$) δ = 140.0 (C), 139.7 (C), 133.4 (C), 128.99 (2CH), 128.98 (2CH), 128.5 (2CH), 127.7 (CH), 127.1 (2CH). **MS** (APCI+) m/z: $[M^{+\cdot}]$ calcd. for $C_{12}H_9Cl$ is 188.04, found 188.06.

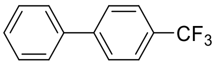

4-(Trifluoromethyl)-1,1'-biphenyl (3ai):[54] $C_{13}H_9F_3$, MW = 222.21 g/mol, yield = 61 %, white solid. **^{1}H NMR** (300 MHz, $CDCl_3$) δ = 7.70 (s, 4H), 7.62-7.58 (m, 2H), 7.51-7.46 (m, 2H), 7.44-7.38 (m, 1H). **^{19}F NMR** (282 MHz, $CDCl_3$) δ = -62.4. **^{13}C NMR** (75 MHz, $CDCl_3$) δ = 144.8 (C), 139.9 (C), 129.4 (q, *J* = 32 Hz, C), 129.2 (2CH), 128.3 (CH), 127.5 (2CH), 127.4 (2CH), 125.8 (q, *J* = 4 Hz, 2CH), 124.4 (q, *J* = 270 Hz, CF_3). **MS** (APCI+) m/z: $[M^{+\cdot}]$ calcd. for $C_{13}H_9F_3$ is 222.06, found 222.21.

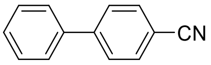

[1,1'-Biphenyl]-4-carbonitrile (3aj):[51] $C_{13}H_9N$, MW = 179.22 g/mol, yield = 56 %, white solid. **^{1}H NMR** (300 MHz, $CDCl_3$) δ = 7.73-7.66 (m, 4H), 7.61-7.58 (m, 2H), 7.52-7.43 (m, 3H). **^{13}C NMR** (75 MHz, $CDCl_3$) δ = 145.6 (C), 139.1 (C), 132.6 (2CH), 129.1 (2CH), 128.7 (CH), 127.7 (2CH), 127.2 (2CH), 119.0 (C), 110.9 (C). **MS** (APCI+) m/z: $[M+2H]^+$ calcd. for $C_{13}H_{11}N$ is 181.09, found 181.12.

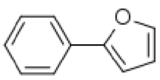

2-phenylfuran (3ak):[53] $C_{10}H_8O$, MW = 144.17 g/mol, yield = 30 %, white solid. **^{1}H NMR** (300 MHz, $CDCl_3$) δ = 7.70-7.66 (m, 2H), 7.47 (dd, *J* = 1.8 Hz, 0.6 Hz, 1H), 7.41-7.35 (m, 2H), 7.29-7.23 (m, 2H), 6.66 (dd, *J* = 3.4 Hz, 0.6 Hz, 1H), 6.48 (dd, *J* = 3.4 Hz, 1.8 Hz, 1H). **^{13}C NMR** (75 MHz, $CDCl_3$) δ = 154.1 (C), 142.2 (CH), 131.0 (C), 128.8 (2CH), 127.4 (CH),123.9 (2CH), 111.7 (CH), 105.1 (CH). **MS** (APCI+) m/z: $[M+H]^+$ calcd. for $C_{10}H_9O$ is 145.06, found 145.14.

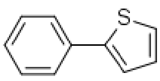

2-phenylthiophene (3al):[54] $C_{10}H_8S$, MW = 160.23 g/mol, yield = 37 %, white solid. **^{1}H NMR** (300 MHz, $CDCl_3$) δ = 7.52-7.48 (m, 2H), 7.29-7.24 (m, 2H), 7.21-7.14 (m, 3H), 6.96 (dd, *J* = 5.1 Hz, 3.6 Hz, 1H). **^{13}C NMR** (75 MHz, $CDCl_3$) δ = 144.5 (C), 134.5 (C), 129.0 (2CH), 128.1 (CH), 127.6 (CH), 126.1 (2CH), 124.9 (CH), 123.2 (CH). **MS** (APCI+) m/z: $[M+H]^+$ calcd. for $C_{10}H_9S$ is 161.04, found 161.12.

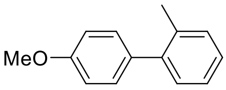

4'-Methoxy-2-methyl-1,1'-biphenyl (3bf):[55] $C_{14}H_{14}O$, MW = 198.27 g/mol, yield = 71 %, white solid. **^{1}H NMR** (300 MHz, $CDCl_3$) δ = 7.28-7.22 (m, 6H), 6.99-9.94 (m, 2H), 3.89 (s, 3H), 2.31 (s, 3H). **^{13}C NMR** (75 MHz, $CDCl_3$) δ = 158.6 (C), 141.7 (C), 135.6 (C), 134.5 (C), 130.4 (CH), 130.3 (2CH), 130.0 (CH), 127.1 (CH), 125.9 (CH), 113.6 (2CH), 55.4 (CH_3), 20.7 (CH_3). **MS** (APCI+) m/z: $[M+H]^+$ calcd. for $C_{14}H_{15}O$ is 199.11, found 199.08.

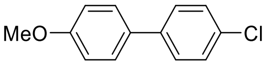

4-Chloro-4'-methoxy-1,1'-biphenyl (3bh):[53] $C_{13}H_{11}ClO$, MW = 218.68 g/mol, yield = 82 %, beige solid. **^{1}H NMR** (300 MHz, $CDCl_3$) δ = 7.33-7.26 (m, 4H), 7.23-7.18 (m, 2H), 6.80 (m, 2H). **^{13}C NMR** (75 MHz, $CDCl_3$) δ = 159.4 (C), 139.2 (C), 132.7 (C), 132.4 (C), 128.9 (2CH), 128.0 (2CH), 127.9 (2CH), 114.4 (2CH), 55.3 (CH_3). **MS** (APCI+) m/z: [$M^{+\cdot}$] calcd. for $C_{13}H_{11}ClO$ is 218.05, found 218.20.

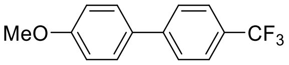

4-Methoxy-4'-(trifluoromethyl)-1,1'-biphenyl (3bi):[56] $C_{14}H_{11}F_3O$, MW = 252.24 g/mol, yield = 62 %, white solid. **^{1}H NMR** (300 MHz, $CDCl_3$) δ = 7.66 (s, 4H), 7.57-7.52 (m, 2H), 7.03-6.98 (m, 2H), 3.87 (1H). **^{19}F NMR** (282 MHz, $CDCl_3$) δ = -62.3. **^{13}C NMR** (75 MHz, $CDCl_3$) δ = 160.0 (C), 144.3 (C), 132.3 (C), 128.8 (q, *J* = 32 Hz, C), 128.5 (2CH), 127.0 (2CH), 125.8 (q, *J* = 4 Hz, 2CH), 124.5 (q, *J* = 270 Hz, CF_3), 114.5 (2CH), 55.5 (CH_3). **MS** (APCI+) m/z: [$M^{+\cdot}$] calcd. for $C_{14}H_{11}F_3O$ is 252.08, found 252.28.

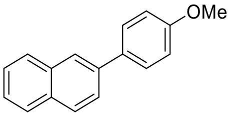

2-(4-Methoxyphenyl)naphthalene (3ca):[50] $C_{17}H_{14}O$, MW = 234.30 g/mol, yield = 77 %, white solid. **^{1}H NMR** (300 MHz, $CDCl_3$) δ = 8.00-7.99 (m, 1H), 7.93-7.83 (m, 3H), 7.72 (dd, *J* = 8.6, 1.8 Hz, 1H), 7.69-7.64 (m, 2H), 7.52-7.43 (m, 2H), 7.05-7.01 (m, 2H), 3.88 (s, 3H). **^{13}C NMR** (75 MHz, $CDCl_3$) δ = 159.4 (C), 138.3 (C), 133.9 (C), 133.7, (C), 132.4 (C), 128.6 (2CH), 128.5 (CH), 128.2 (CH), 127.7 (CH), 126.4 (CH), 125.8 (CH), 125.5 (CH), 125.1 (CH), 114.4 (2CH), 55.5 (CH_3). **MS** (APCI+) m/z: $[M+H]^+$ calcd. for $C_{17}H_{15}O$ is 235.11, found 235.13.

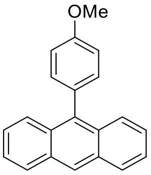

9-(4-Methoxyphenyl)anthracene (3da):[57] $C_{21}H_{16}O$, MW = 284.36 g/mol, yield = 65 %, white solid. **^{1}H NMR** (300 MHz, $CDCl_3$) δ =8.49 (s, 1H), 8.04 (d, *J* = 8.5 Hz, 2H), 7.72 (d, *J* = 8.8 Hz, 2H), 7.50-7.42 (m, 2H), 7.39-7.31 (m, 4H), 7.16-7.08 (m, 2H), 3.95 (s, 3H). **^{13}C NMR** (75 MHz, $CDCl_3$) δ = 159.1 (C), 136.9 (C), 132.4 (2CH), 131.5 (2C), 130.9 (C), 130.7 (2C), 128.4 (2CH), 127.0 (2CH), 126.5 (CH), 125.3 (2CH), 125.2 (2CH), 113.9 (2CH), 55.4 (CH_3). **MS** (APCI+) m/z: $[M+2H]^+$ calcd. for $C_{21}H_{18}O$ is 286.13, found 286.18.

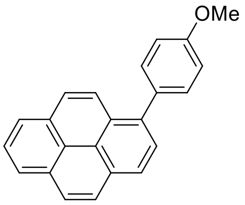

3-(4-Methoxyphenyl)-1,5*a*[1]-dihydropyrene (3ea):[55] $C_{23}H_{16}O$, MW = 308.38 g/mol, yield = 60 %, white solid. **^{1}H NMR** (300 MHz, $CDCl_3$) δ = 8.25-8.16 (m, 4H), 8.10 (s, 2H), 8.05-7.97 (m, 3H), 7.61-7.56 (m, 2H), 7.15-7.10 (m, 2H), 3.94 (3H). **^{13}C NMR** (75 MHz, $CDCl_3$) δ = 159.1 (C), 137.6 (C), 133.7 (C), 131.8 (2CH), 131.6 (C) 131.1 (C), 130.5 (C), 128.7 (C), 127.8 (CH), 127.6 (CH), 127.5 (CH), 127.4 (CH), 126.1 (CH), 125.5 (CH), 125.1 (CH), 124.8 (CH), 127.8 (CH), 114.0 (2CH), 55.5 (CH_3). **MS** (APCI+) m/z: $[M+2H]^+$ calcd. for $C_{23}H_{16}O$ is 310.12, found 310.16.

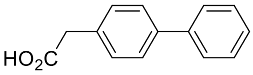

2-([1,1'-Biphenyl]-4-yl)acetic acid (*felbinac*, 3fj):[58] $C_{14}H_{12}O_2$, MW = 212.25 g/mol, yield = 63 %, white solid. **^{1}H NMR** (300 MHz, DMSO d_6) δ = 12.40 (bs, 1H), 7.68-7.64 (m, 2H), 7.62-7.60 (m, 2H), 7.49-7.44 (m, 2H), 7.39-7.34 (m, 3H), 3.62 (s, 2H). **^{13}C NMR** (75 MHz, DMSO d_6) δ = 172.8 (C), 140.0 (C), 138.5 (C), 134.3 (C), 130.0 (2CH), 129.0 (2CH), 127.4 (CH), 126.61 (2CH), 126.59 (2CH), 40.3 (C). **MS** (APCI+) m/z: $[M+H]^+$ calcd. for $C_{14}H_{13}O_2$ is 213.08, found 213.18.

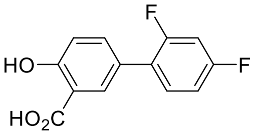

2',4'-Difluoro-4-hydroxy-[1,1'-biphenyl]-3-carboxylic acid (*diflunisal*, 3gk):[59] $C_{13}H_8F_2O_3$, MW = 250.20 g/mol, yield = 70 %, white solid. **^{1}H NMR** (300 MHz, DMSO d_6) δ = 7.92 (t, *J* = 1.9 Hz, 1H), 7.68 (dt, *J* = 8.6 Hz, 1.8 Hz, 1H), 7.58 (td, *J* = 8.9 Hz, 6.6 Hz, 1H), 7.36 (ddd, *J* = 11.6 Hz, 9.3 Hz, 2.6 Hz, 1H), 7.16 (td, *J* = 8.5 Hz, 3.5 Hz, 1H), 7.08 (d, *J* = 8.6 Hz, 1H). **^{19}F NMR** (282 MHz, $CDCl_3$) δ = -111.9 (d, *J* = 6.8 Hz, 1F), -114.1 (d, *J* = 6.9 Hz, 1F). **^{13}C NMR** (75 MHz, DMSO d_6) δ = 171.6 (C=O), 161.5 (dd, *J* = 245 Hz, 12 Hz, C-F), 160.8 (C), 159.0 (dd, *J* = 246 Hz, 12 Hz, C-F), 135.9 (d, *J* = 2 Hz, CH), 131.6 (dd, *J* = 10 Hz, 5 Hz, CH), 130.3 (d, *J* = 2 Hz, C), 125.2 (CH), 123.8 (dd, *J* = 13 Hz, 4 Hz, C), 117.7 (CH), 113.3 (C), 112.1 (dd, *J* = 21 Hz, 4 Hz, CH), 104.7 (t, *J* = 26 Hz, CH). **MS** (APCI+) m/z: $[M+H]^+$ calcd. for $C_{13}H_9F_2O_3$ is 251.04, found 251.15.

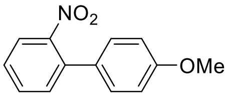

4'-methoxy-2-nitrobiphenyl (3ha):[60] $C_{13}H_{11}NO_3$, MW = 229.24 g/mol, yield = 75 %, yellow solid. **^{1}H NMR** (300 MHz, $CDCl_3$) δ = 7.84-7.81 (m, 1H), 7.64-7.58 (m, 1H), 7.48-7.44 (m, 2H), 7.30-7.27 (m, 2H), 7.00-6.97 (m, 2H), 3.86 (s, 3H). **^{13}C NMR** (75 MHz, $CDCl_3$) δ = 159.6 (C), 149.3 (C), 135.7 (C), 132.2 (CH), 131.9 (CH), 129.4 (C), 129.1 (2CH), 127.7 (CH), 123.9 (CH), 114.2 (2CH), 55.2 (CH_3). **MS** (APCI+) m/z: $[M+H]^+$calcd. for $C_{13}H_{13}NO_3$ is 231.09, found 231.04.

8. ^{1}H and ^{13}C spectrum of compounds 3

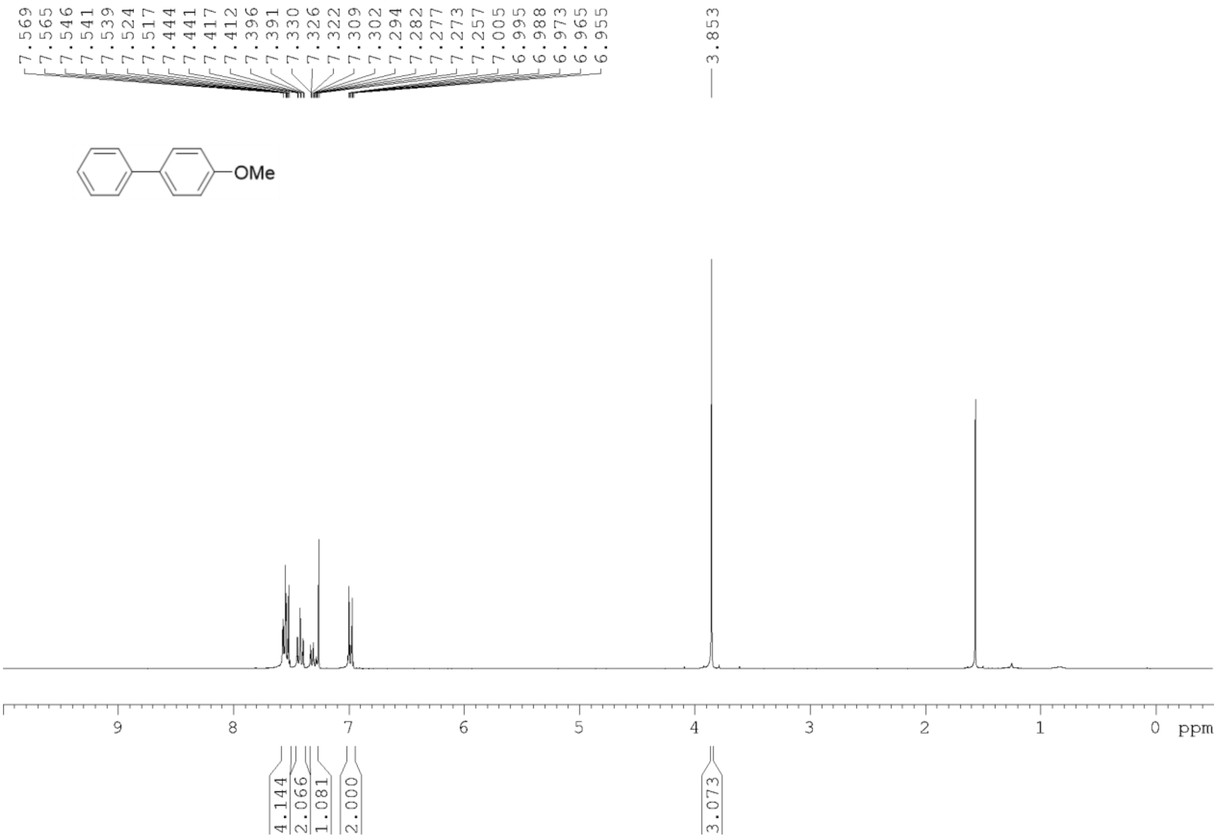

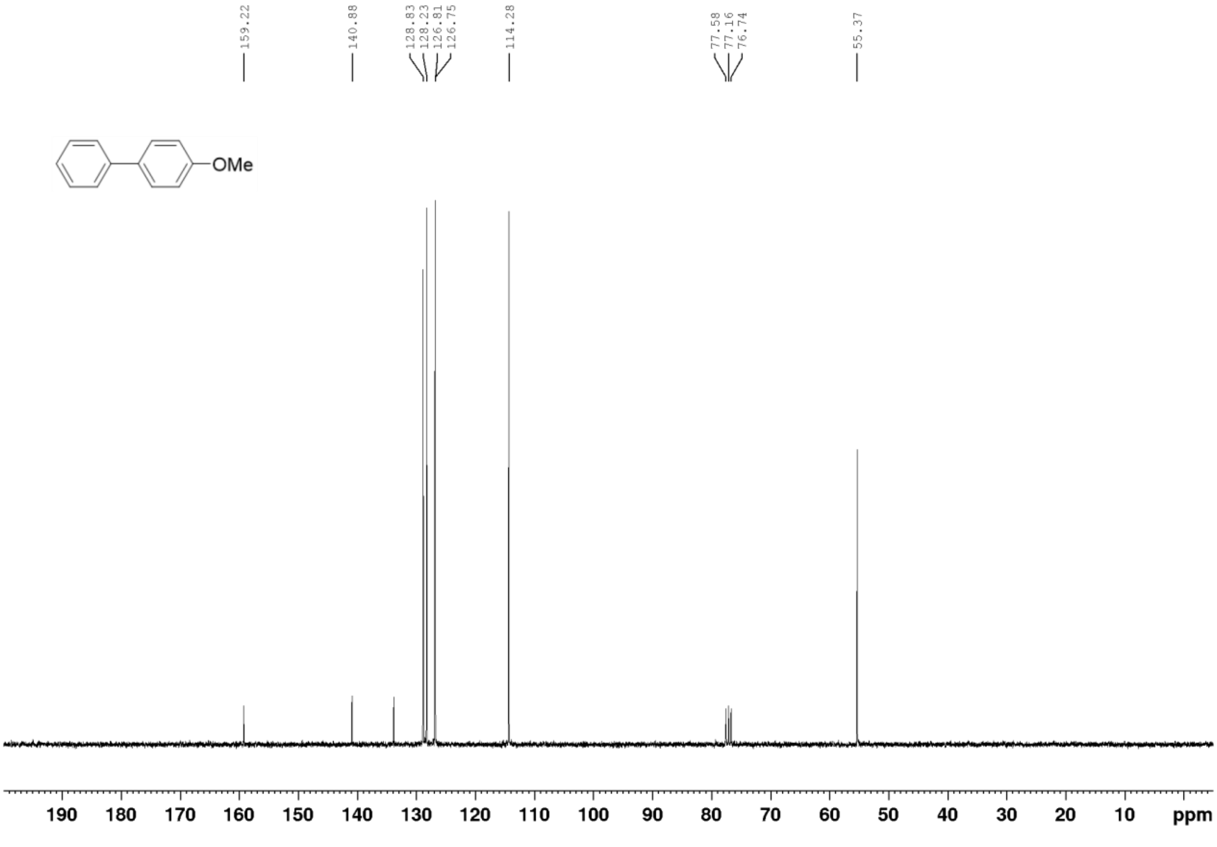

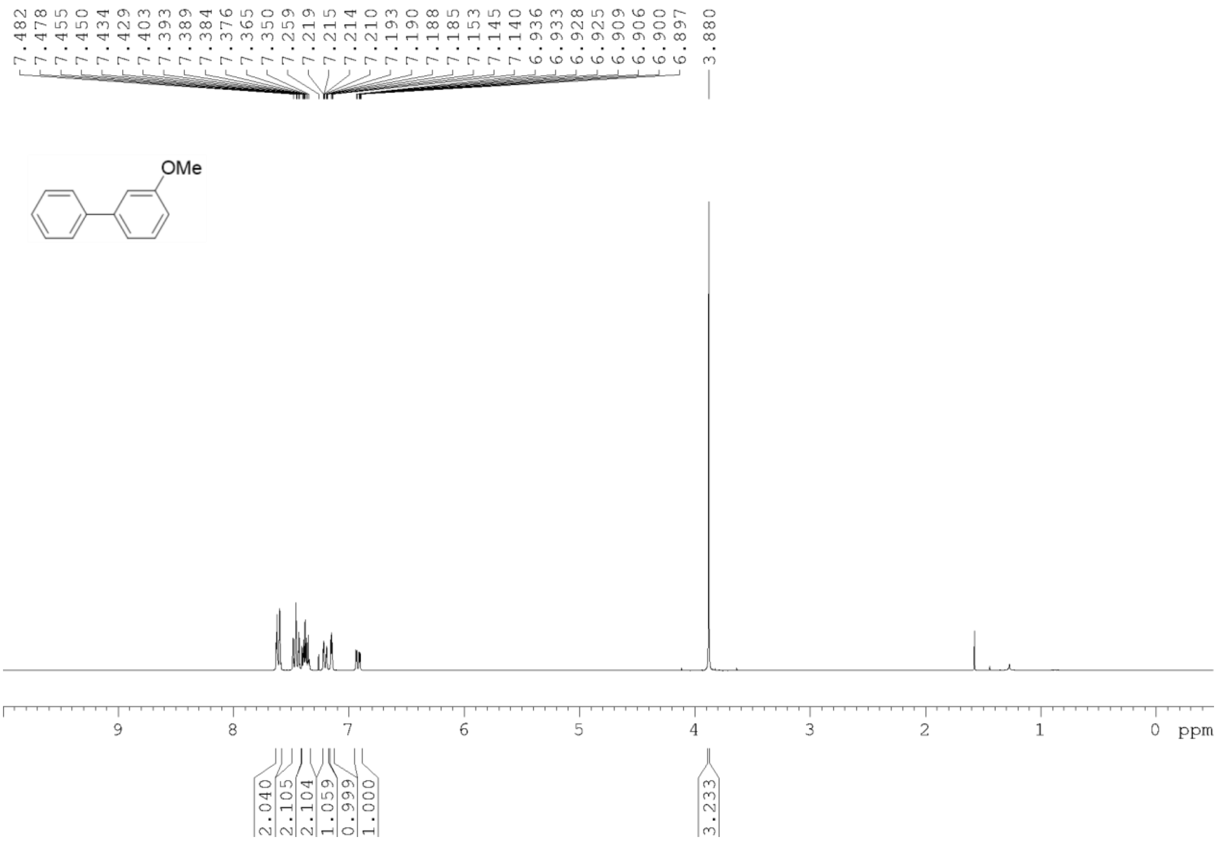
7.482
7.478
7.455
7.450
7.434
7.429
7.403
7.393
7.389
7.384
7.376
7.365
7.350
7.259
7.219
7.215
7.214
7.210
7.193
7.190
7.188
7.185
7.153
7.145
7.140
6.936
6.933
6.928
6.925
6.909
6.906
6.900
6.897
3.880
OMe
2.040
2.105
2.104
1.059
0.999
1.000
3.233
ppm

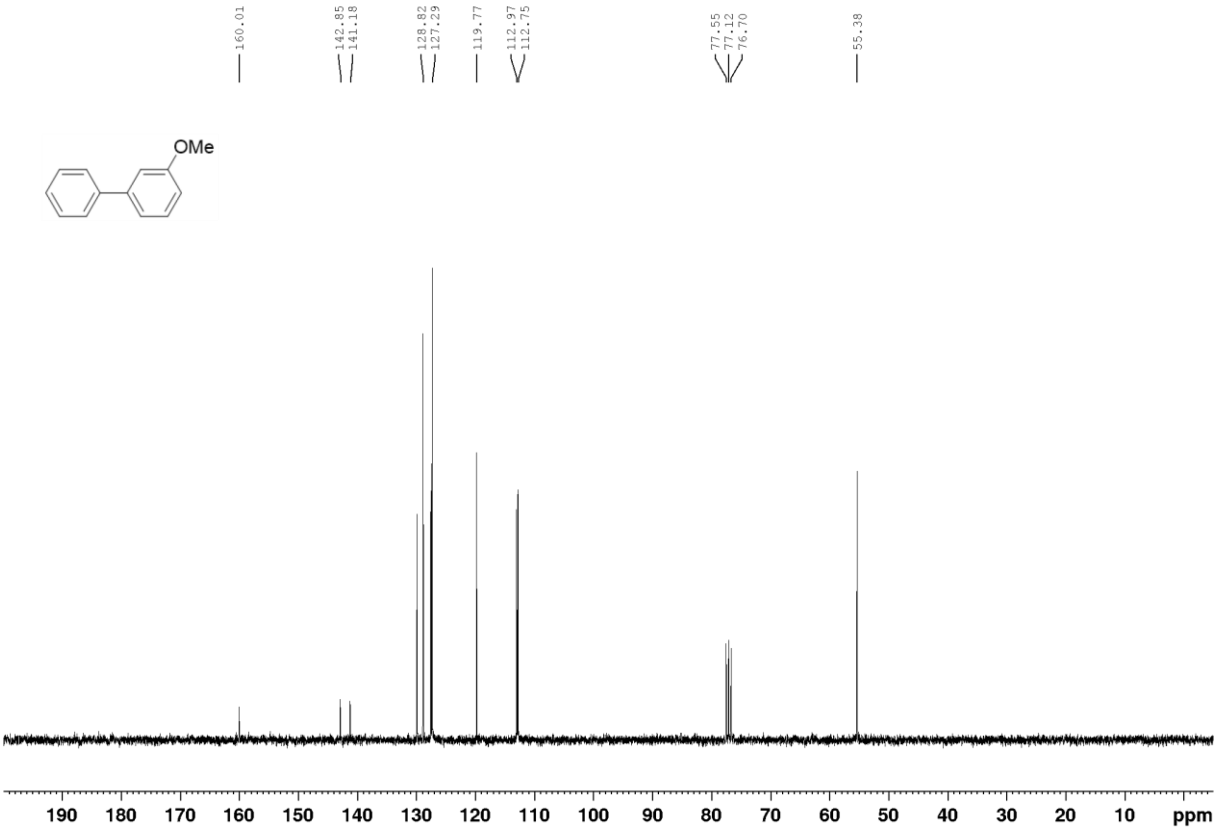
160.01
142.85
141.18
128.82
127.29
119.77
112.97
112.75
77.55
77.12
76.70
55.38
OMe
ppm

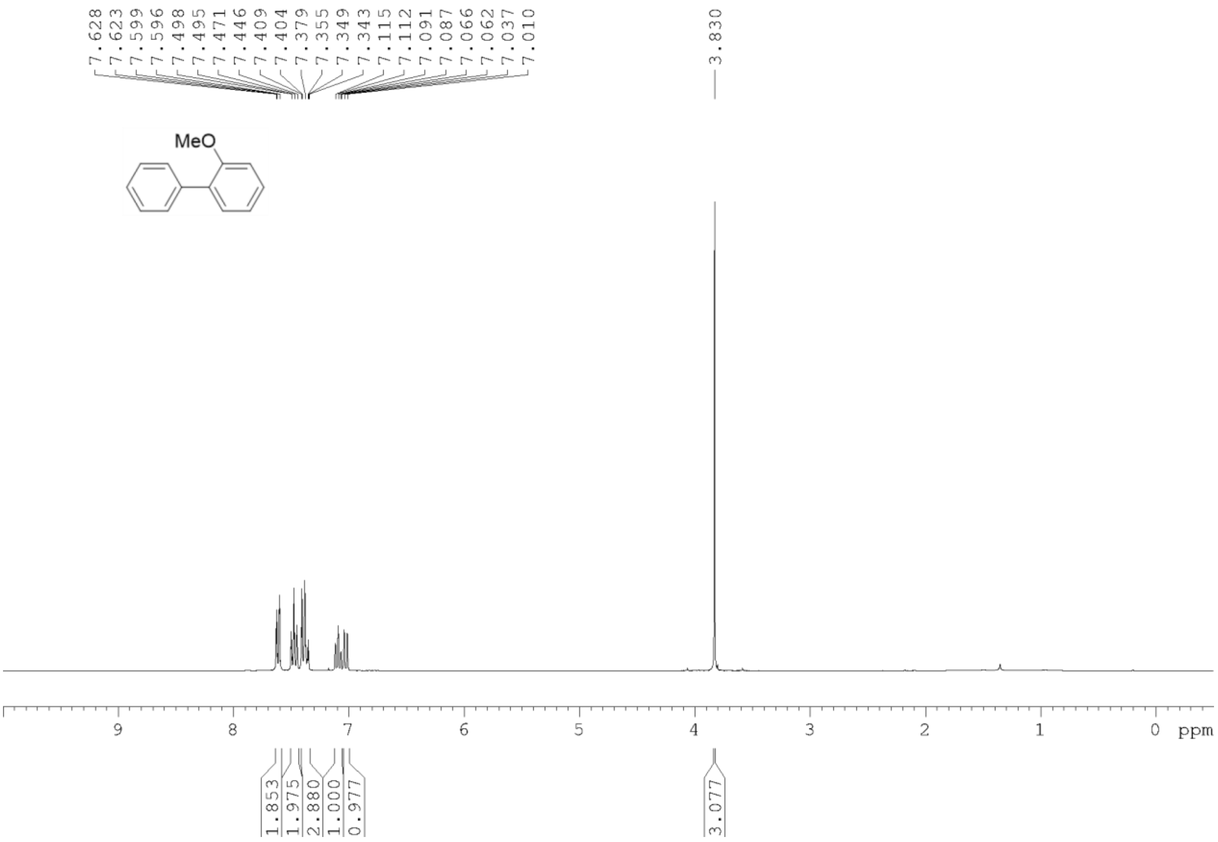
7.628
7.623
7.599
7.596
7.498
7.495
7.471
7.446
7.409
7.404
7.379
7.355
7.349
7.343
7.115
7.112
7.091
7.087
7.066
7.062
7.037
7.010
3.830
MeO
9
8
7
6
5
4
3
2
1
0 ppm
1.853
1.975
2.880
1.000
0.977
3.077

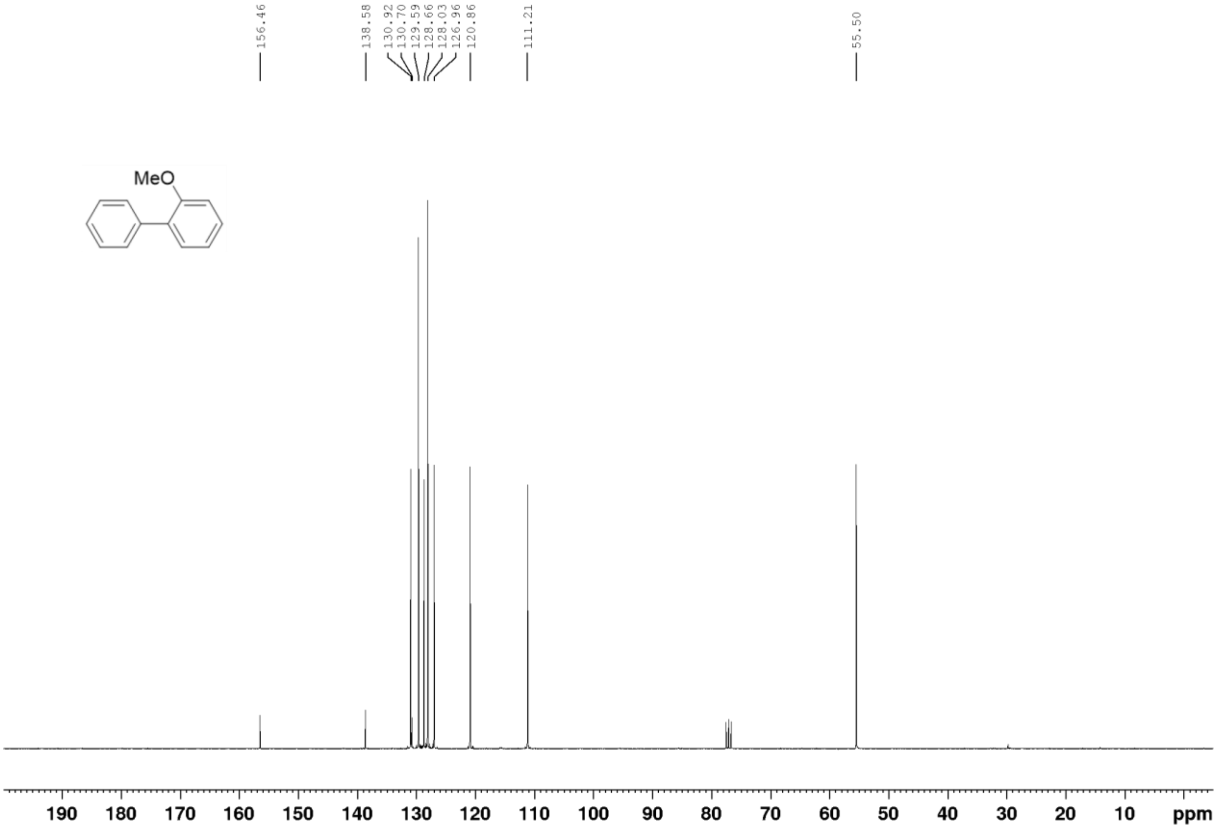
156.46
138.58
130.92
130.70
129.59
128.66
128.03
126.96
120.86
111.21
55.50
MeO
190
180
170
160
150
140
130
120
110
100
90
80
70
60
50
40
30
20
10
ppm

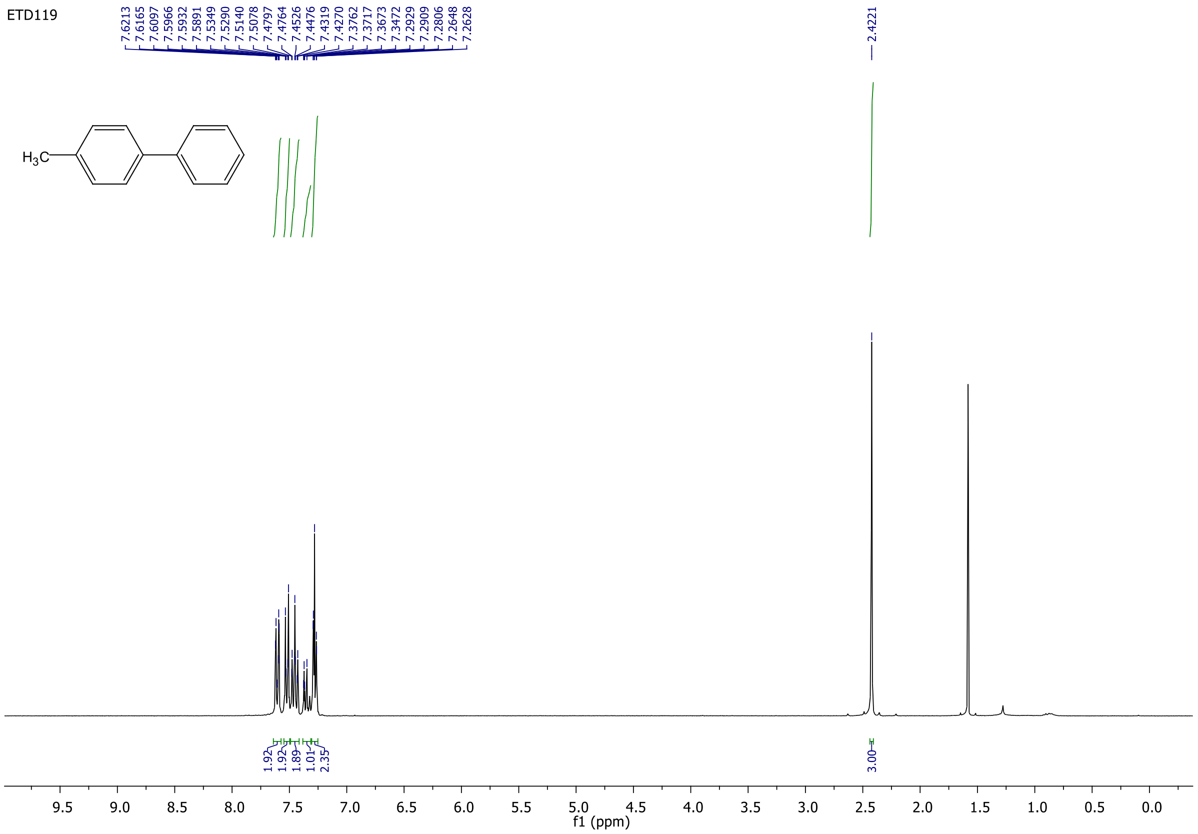
ETD119
7.6213
7.6165
7.6097
7.5966
7.5932
7.5891
7.5349
7.5290
7.5140
7.5078
7.4797
7.4764
7.4526
7.4476
7.4319
7.4270
7.3762
7.3717
7.3673
7.3472
7.2929
7.2909
7.2806
7.2648
7.2628
2.4221
H3C
1.92
1.92
1.89
1.01
2.35
3.00
f1 (ppm)

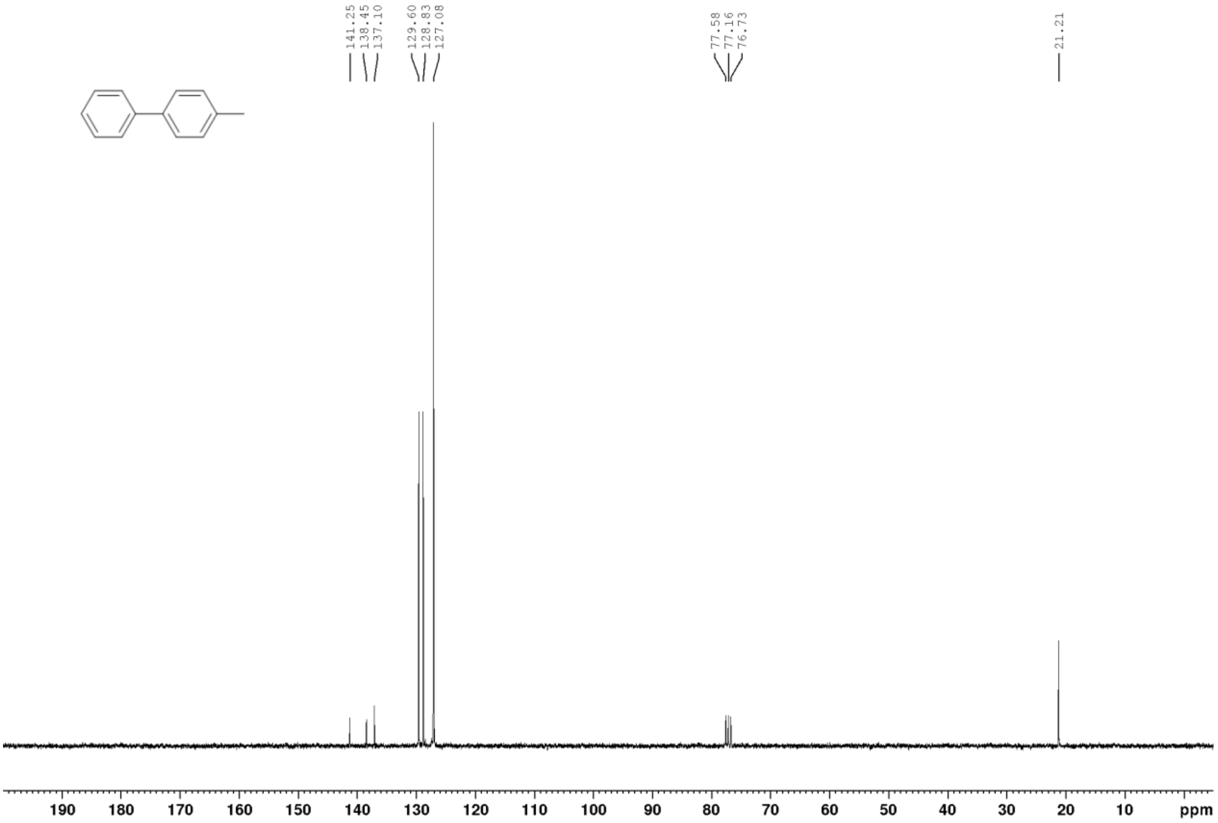
141.25
138.45
137.10
129.60
128.83
127.08
77.58
77.16
76.73
21.21
ppm

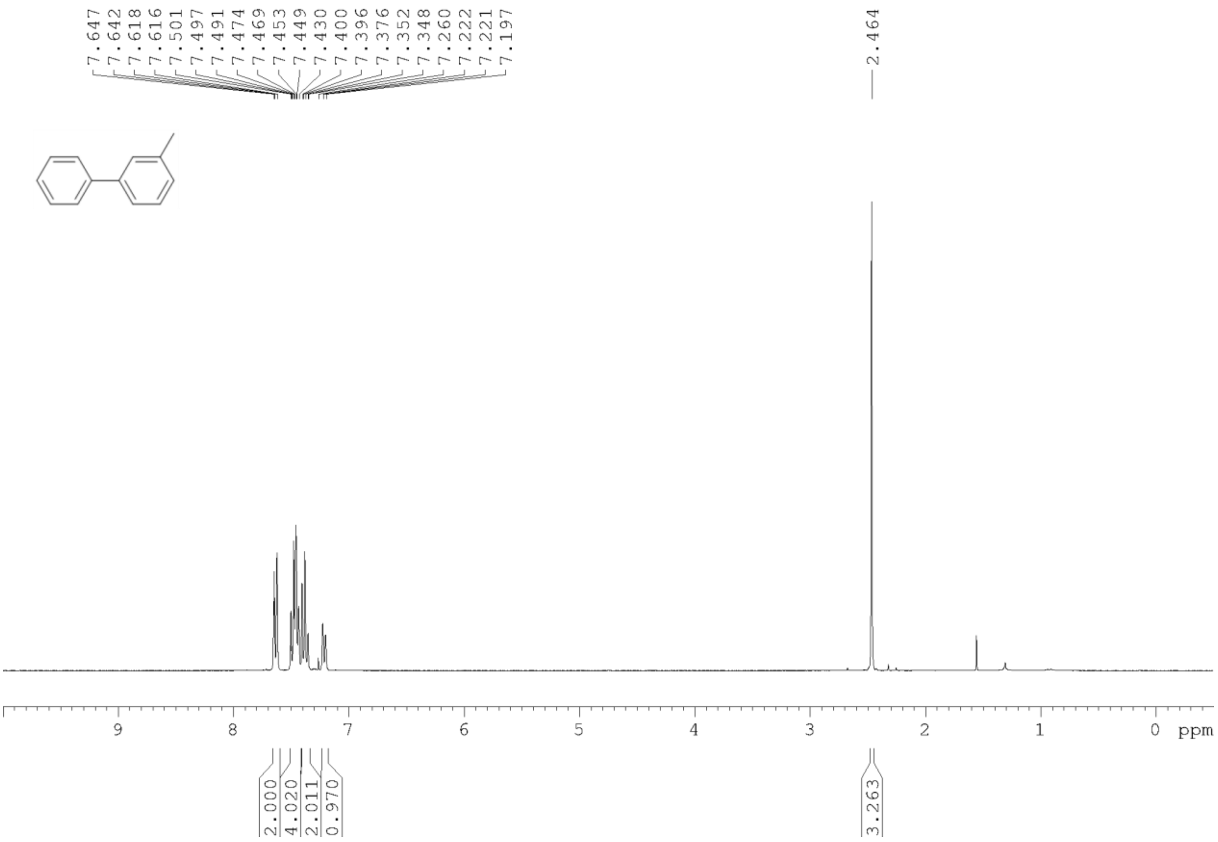
7.647
7.642
7.618
7.616
7.501
7.497
7.491
7.474
7.469
7.453
7.449
7.430
7.400
7.396
7.376
7.352
7.348
7.260
7.222
7.221
7.197
2.464
ppm
2.000
4.020
2.011
0.970
3.263

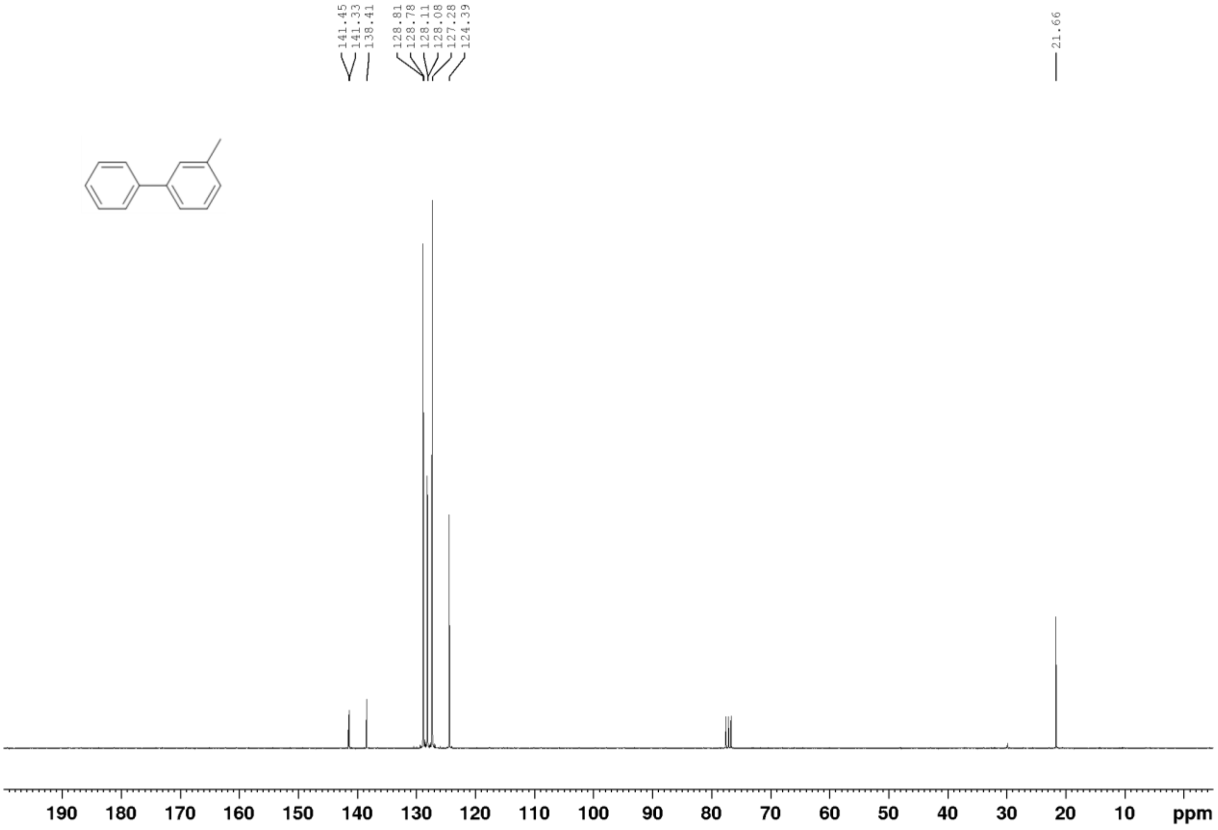
141.45
141.33
138.41
128.81
128.78
128.11
128.08
127.28
124.39
21.66
ppm

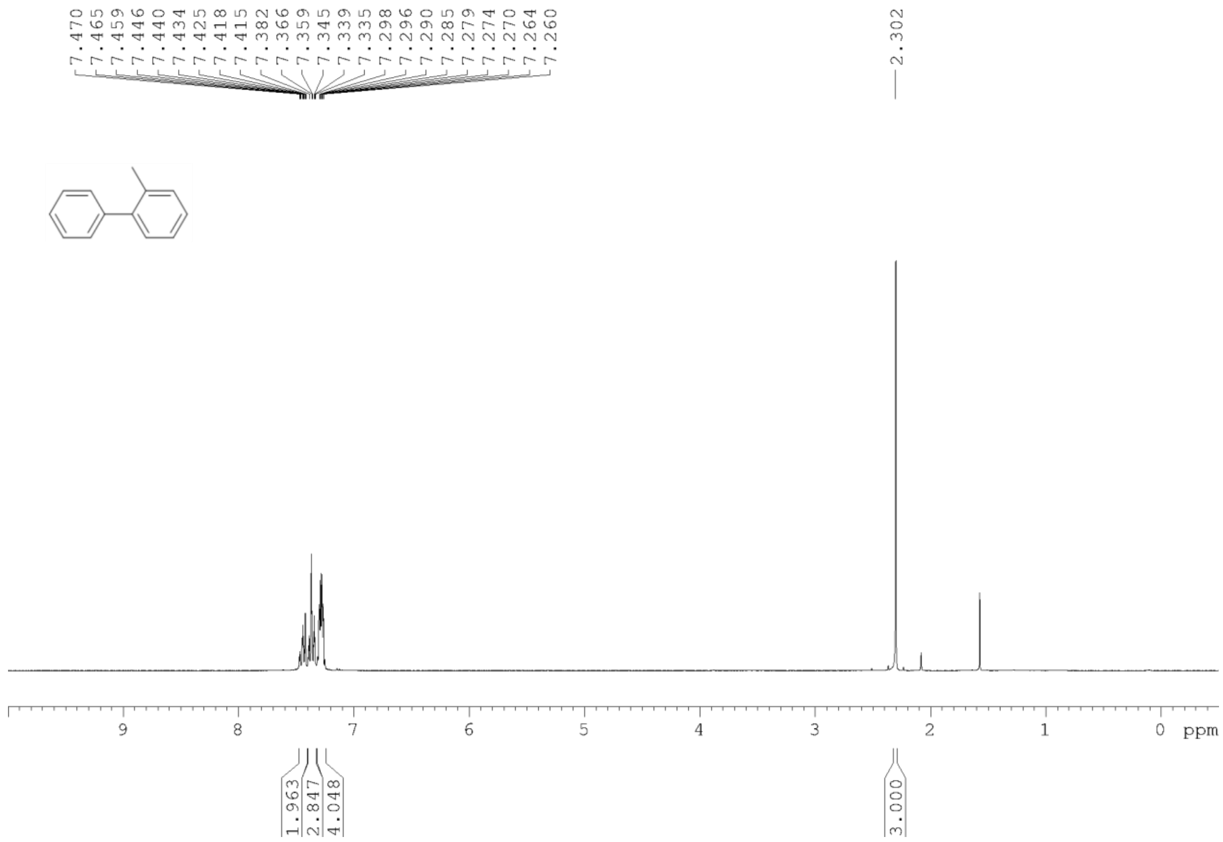
7.470
7.465
7.459
7.446
7.440
7.434
7.425
7.418
7.415
7.382
7.366
7.359
7.345
7.339
7.335
7.298
7.296
7.290
7.285
7.279
7.274
7.270
7.264
7.260
2.302
9 8 7 6 5 4 3 2 1 0 ppm
1.963
2.847
4.048
3.000

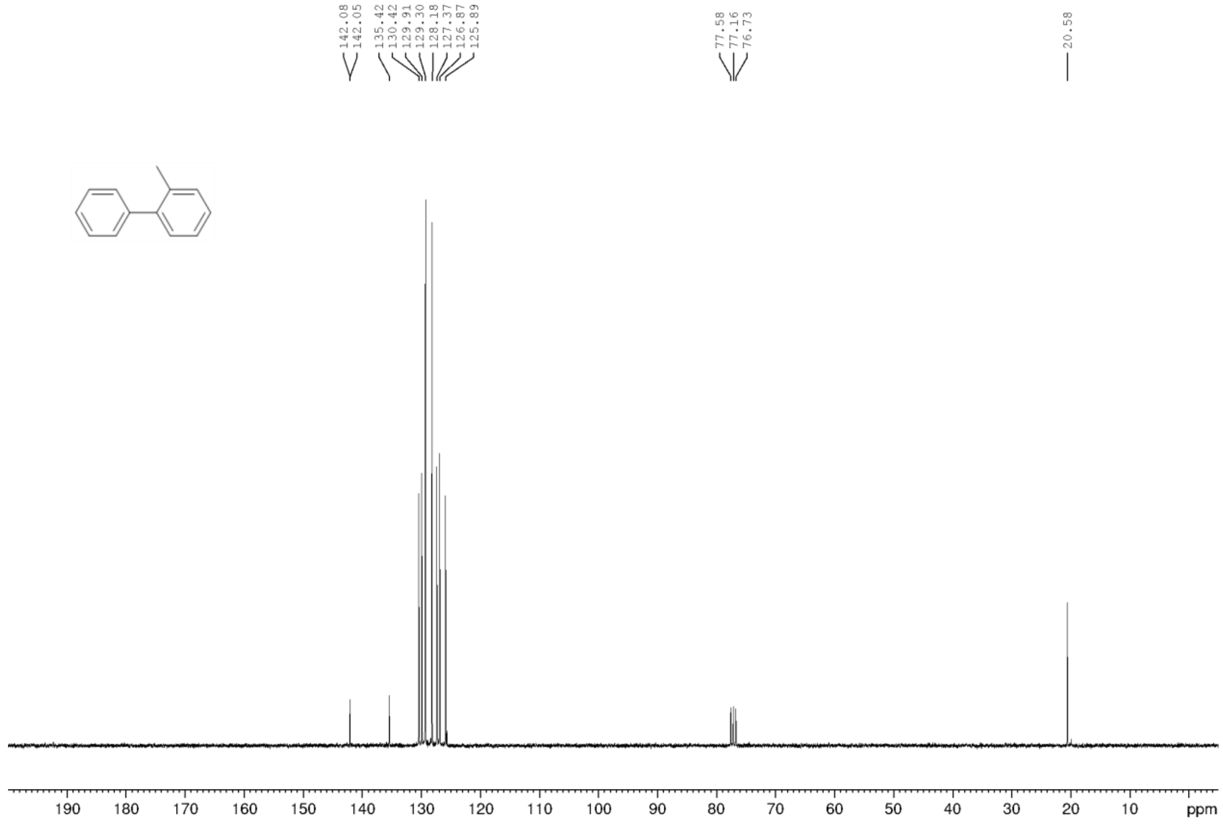
142.08
142.05
135.42
130.42
129.91
129.30
128.18
127.37
126.87
125.89
77.58
77.16
76.73
20.58
190 180 170 160 150 140 130 120 110 100 90 80 70 60 50 40 30 20 10 ppm

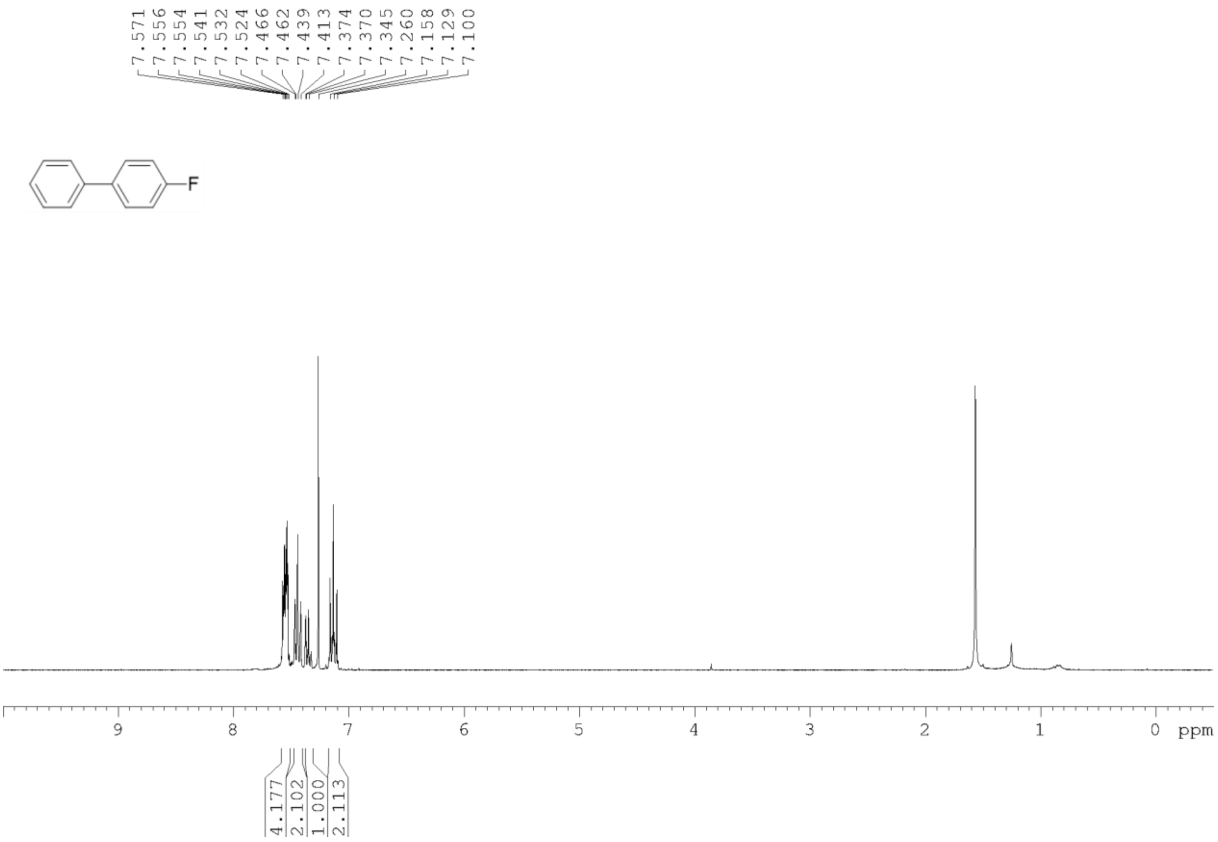

7.571
7.556
7.554
7.541
7.532
7.524
7.466
7.462
7.439
7.413
7.374
7.370
7.345
7.260
7.158
7.129
7.100
F
9 8 7 6 5 4 3 2 1 0 ppm
4.177
2.102
1.000
2.113

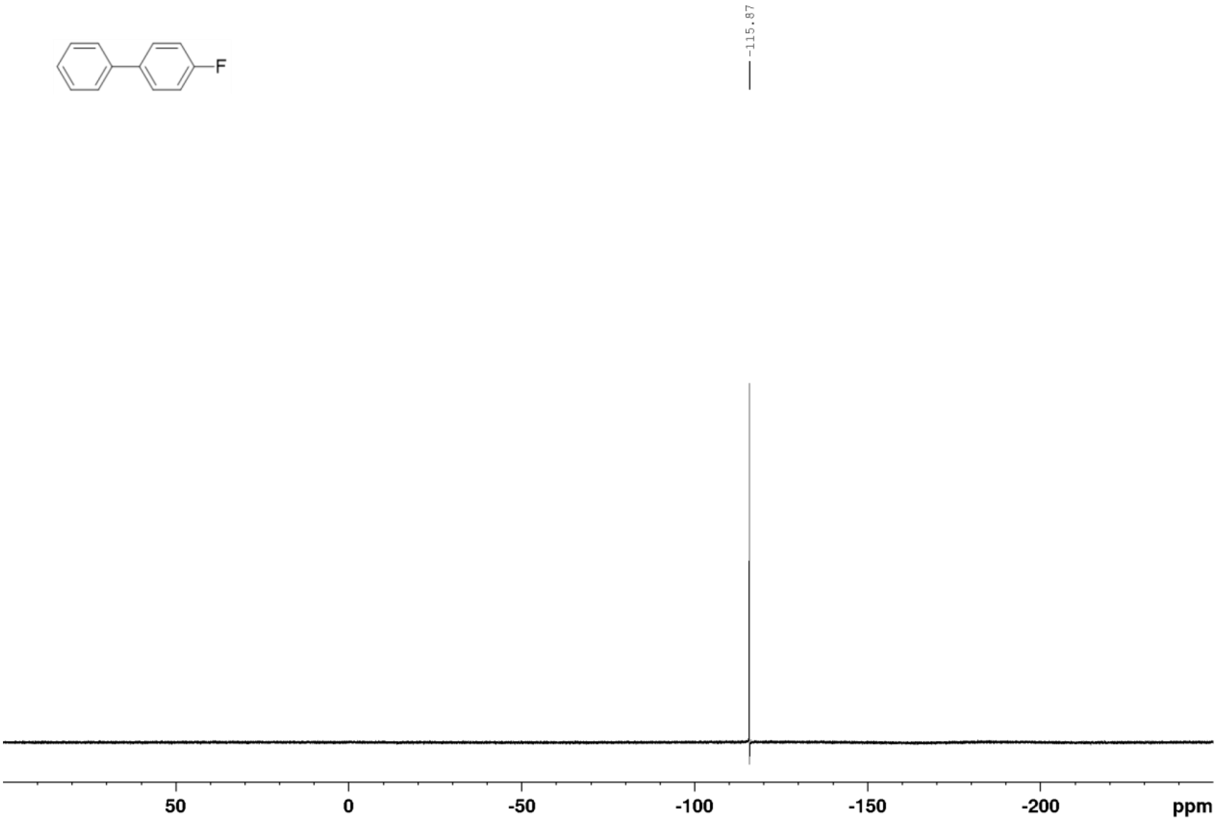

-115.87
F
50 0 -50 -100 -150 -200 ppm

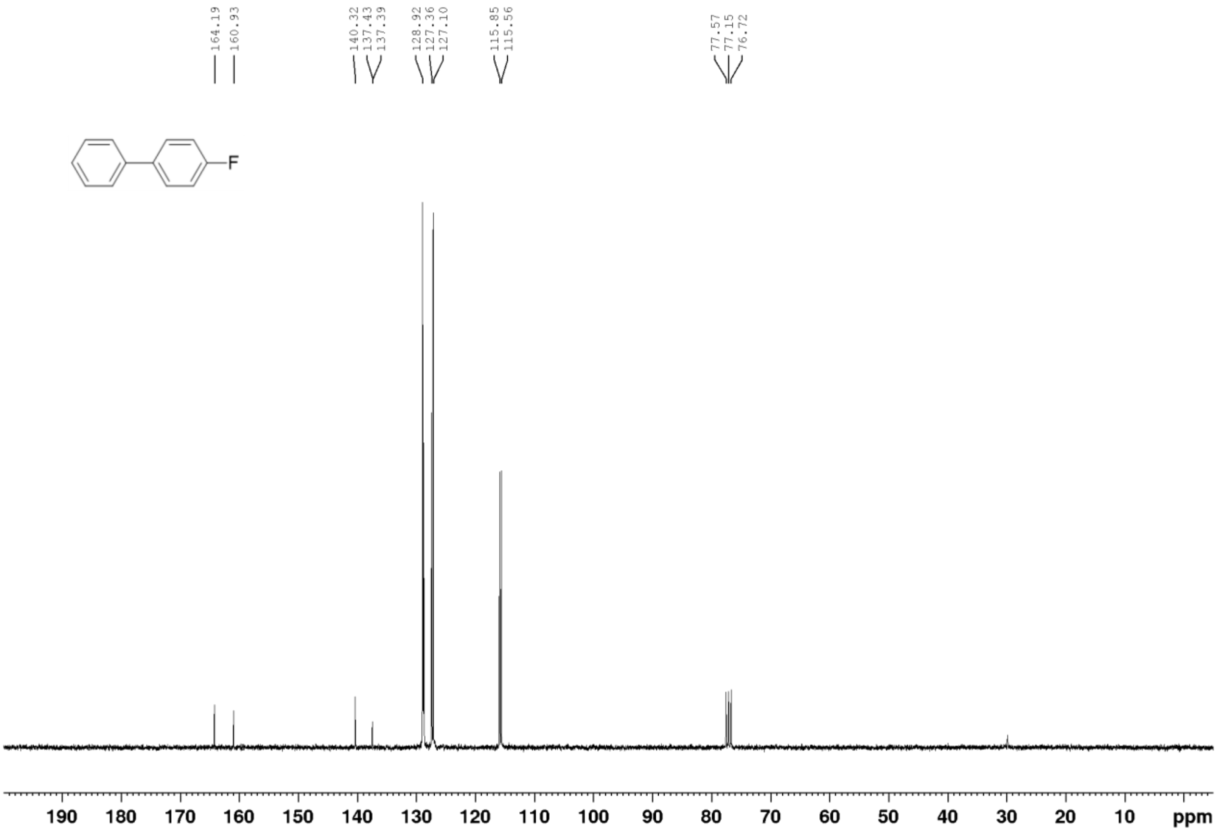
164.19
160.93
140.32
137.43
137.39
128.92
127.36
127.10
115.85
115.56
77.57
77.15
76.72
F
190 180 170 160 150 140 130 120 110 100 90 80 70 60 50 40 30 20 10 ppm

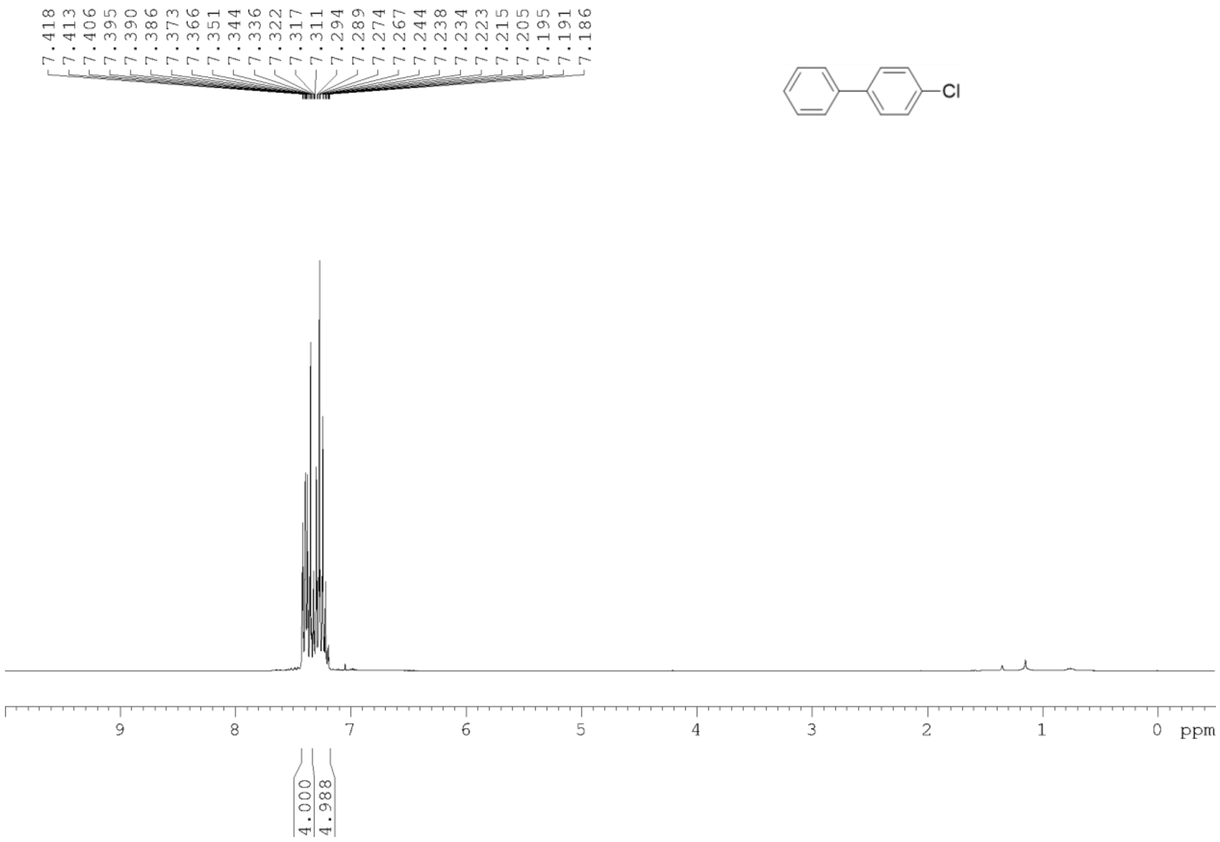
7.418
7.413
7.406
7.395
7.390
7.386
7.373
7.366
7.351
7.344
7.336
7.322
7.317
7.311
7.294
7.289
7.274
7.267
7.244
7.238
7.234
7.223
7.215
7.205
7.195
7.191
7.186
Cl
9 8 7 6 5 4 3 2 1 0 ppm
4.000
4.988

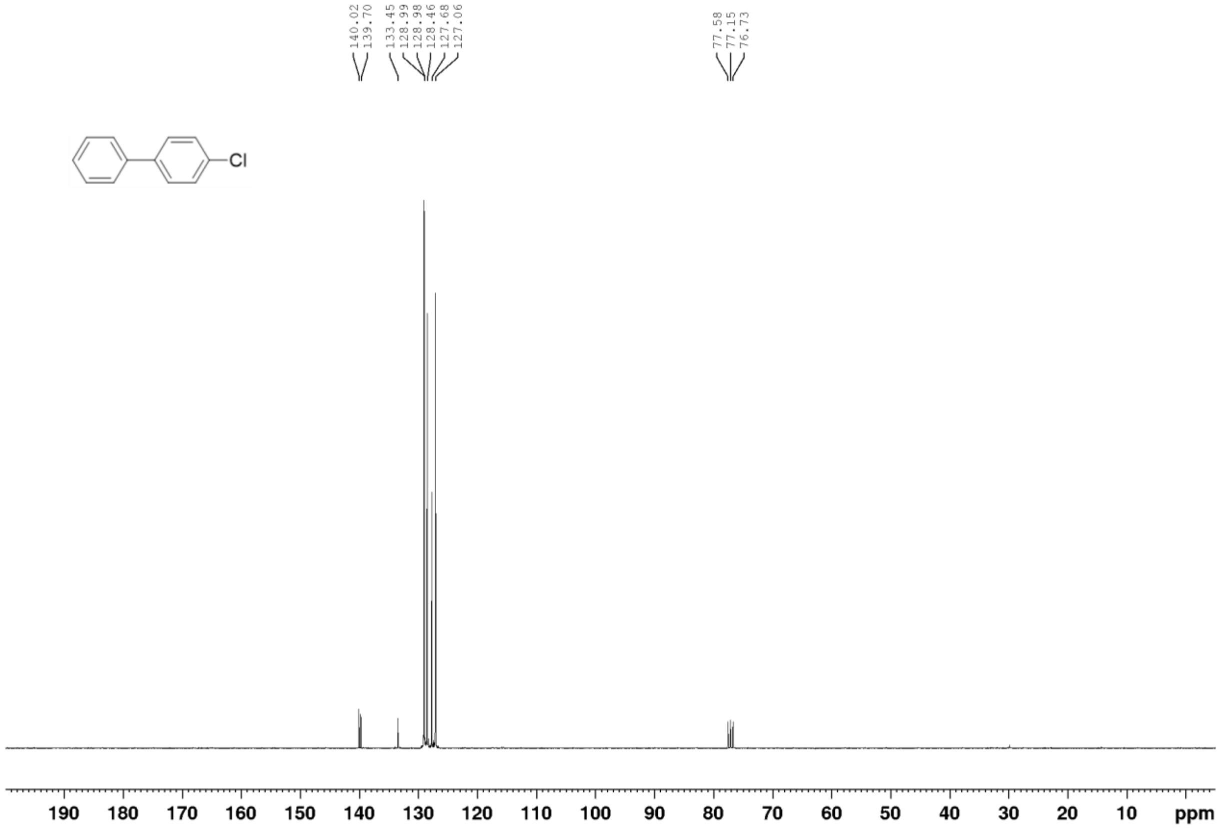
140.02
139.70
133.45
128.99
128.98
128.46
127.68
127.06
77.58
77.15
76.73
Cl
190 180 170 160 150 140 130 120 110 100 90 80 70 60 50 40 30 20 10 ppm

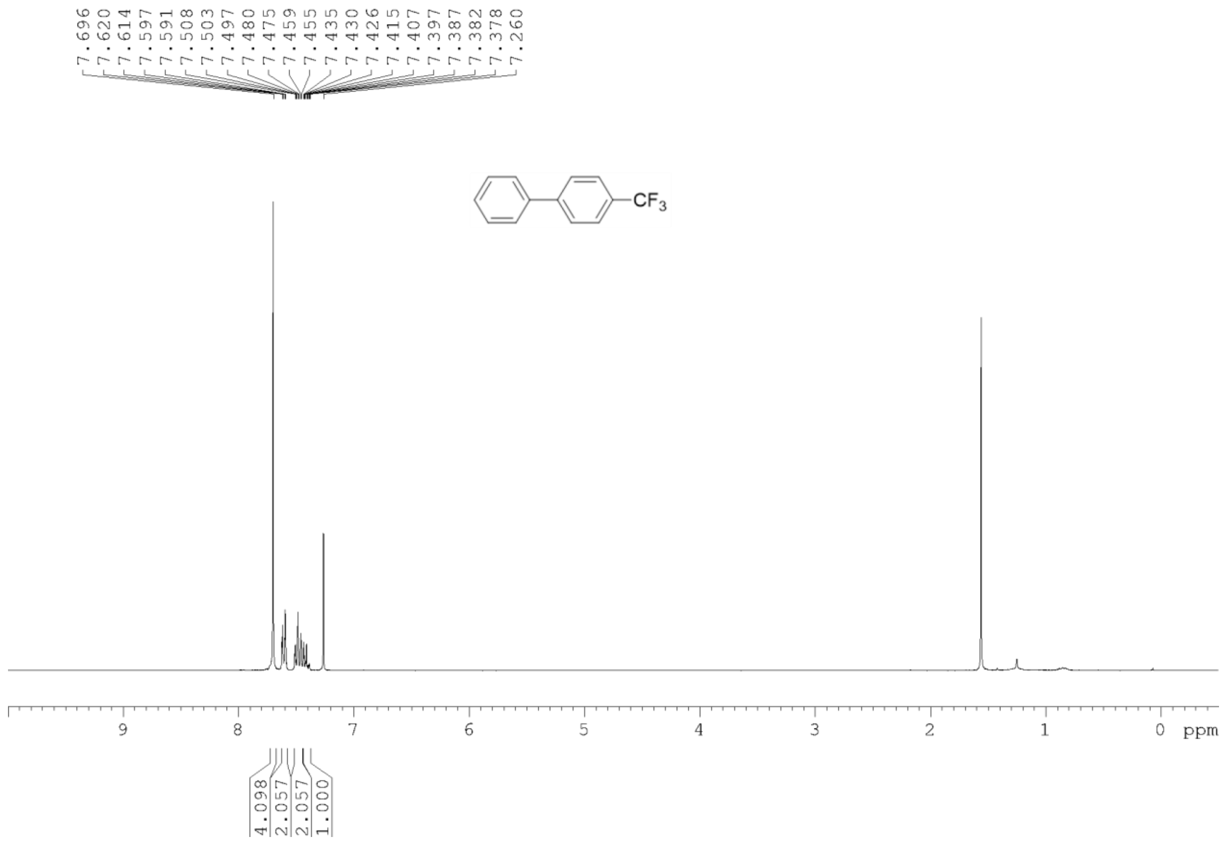
7.696
7.620
7.614
7.597
7.591
7.508
7.503
7.497
7.480
7.475
7.459
7.455
7.435
7.430
7.426
7.415
7.407
7.397
7.387
7.382
7.378
7.260
CF3
9
8
7
6
5
4
3
2
1
0 ppm
4.098
2.057
2.057
1.000

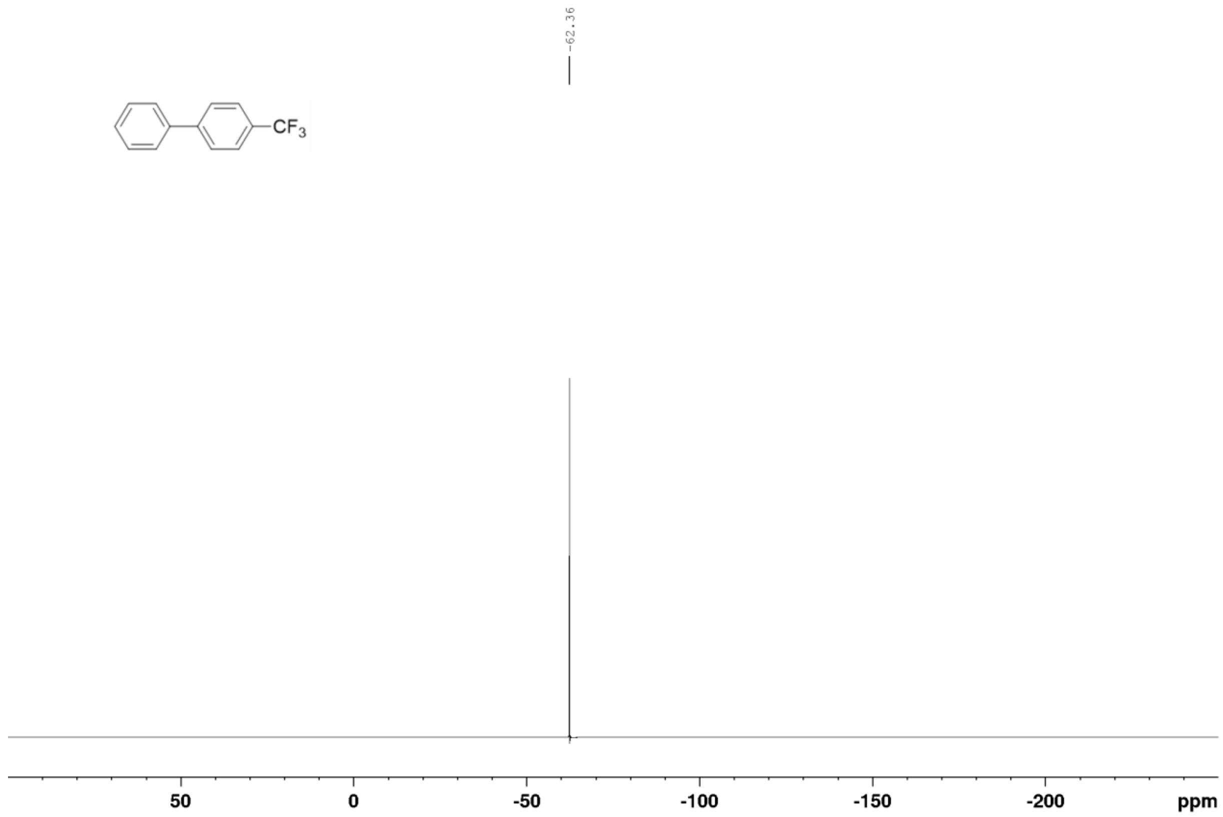
-62.36
CF3
50
0
-50
-100
-150
-200
ppm

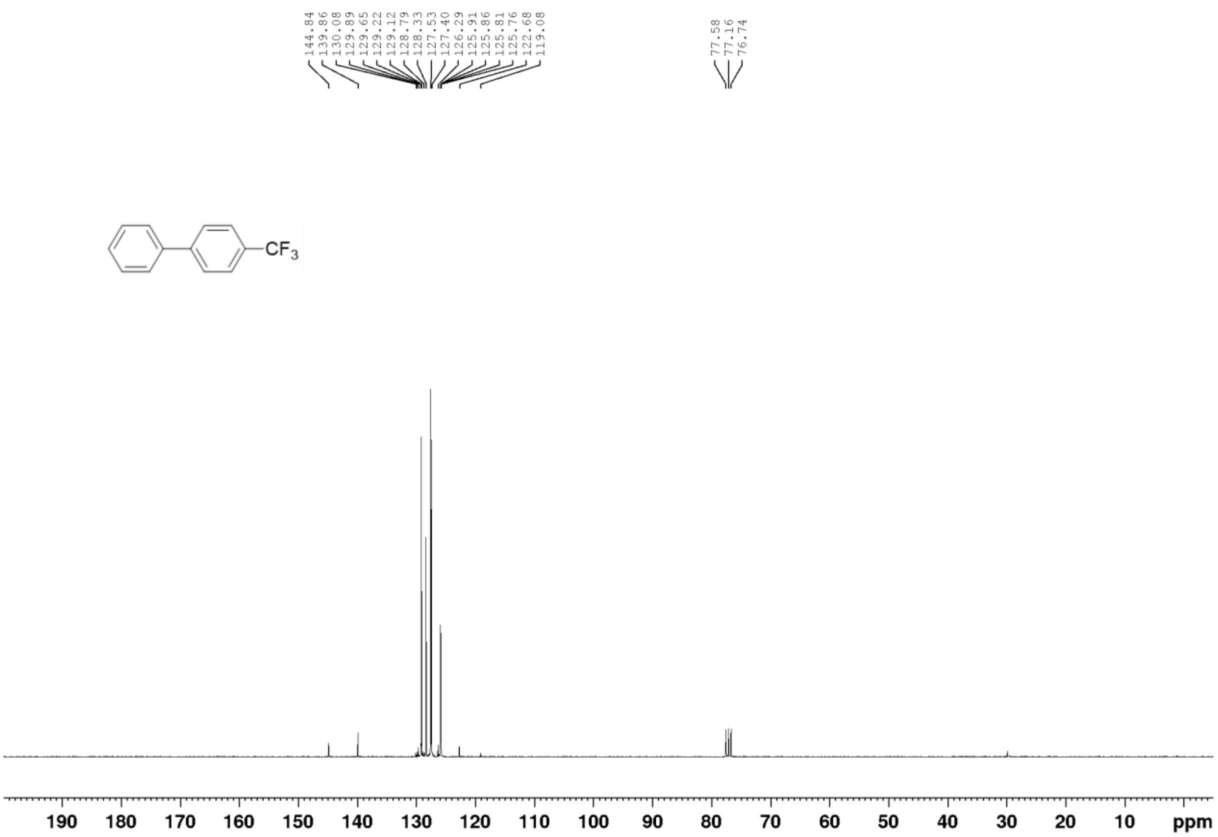

144.84
139.86
130.08
129.89
129.65
129.22
129.12
128.79
128.33
127.53
127.40
126.29
125.91
125.86
125.81
125.76
122.68
119.08
77.58
77.16
76.74
CF3
190 180 170 160 150 140 130 120 110 100 90 80 70 60 50 40 30 20 10 ppm

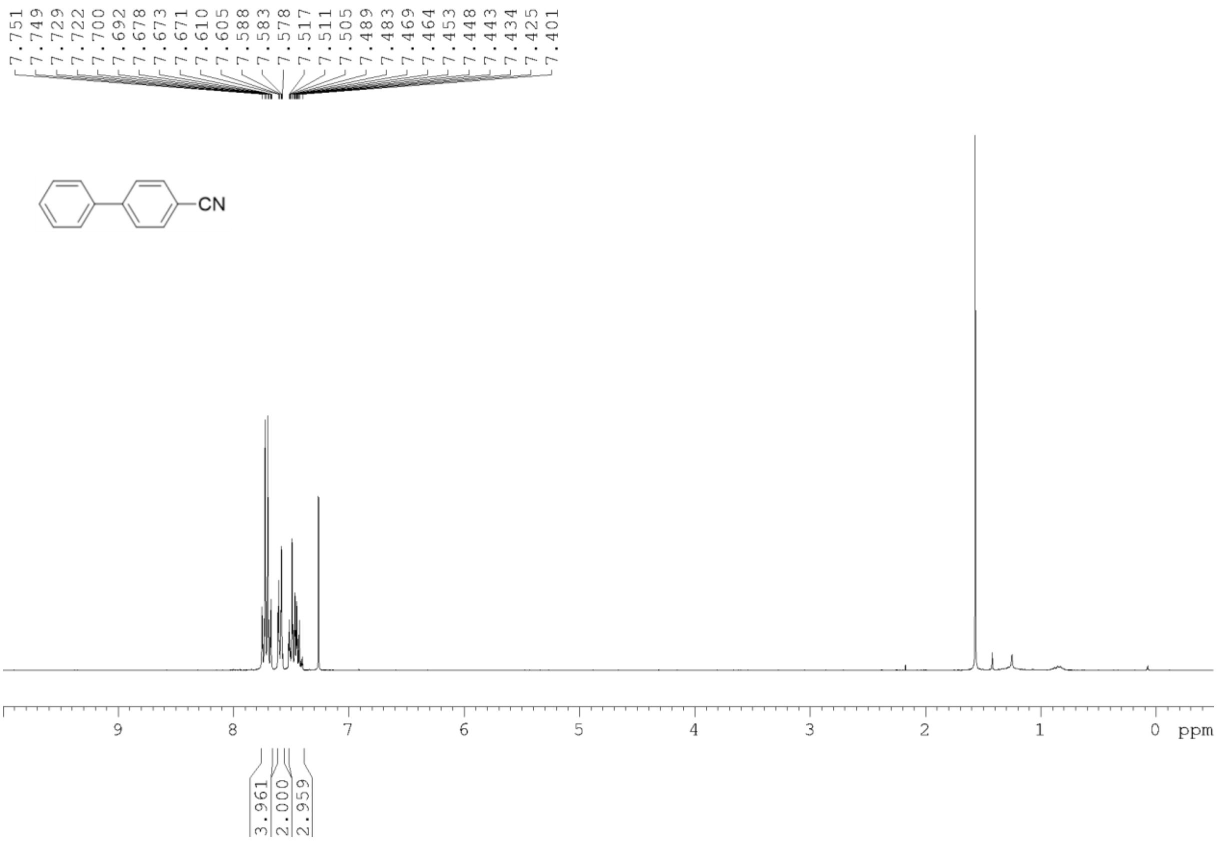
7.751
7.749
7.729
7.722
7.700
7.692
7.678
7.673
7.671
7.610
7.605
7.588
7.583
7.578
7.517
7.511
7.505
7.489
7.483
7.469
7.464
7.453
7.448
7.443
7.434
7.425
7.401
CN
9
8
7
6
5
4
3
2
1
0 ppm
3.961
2.000
2.959

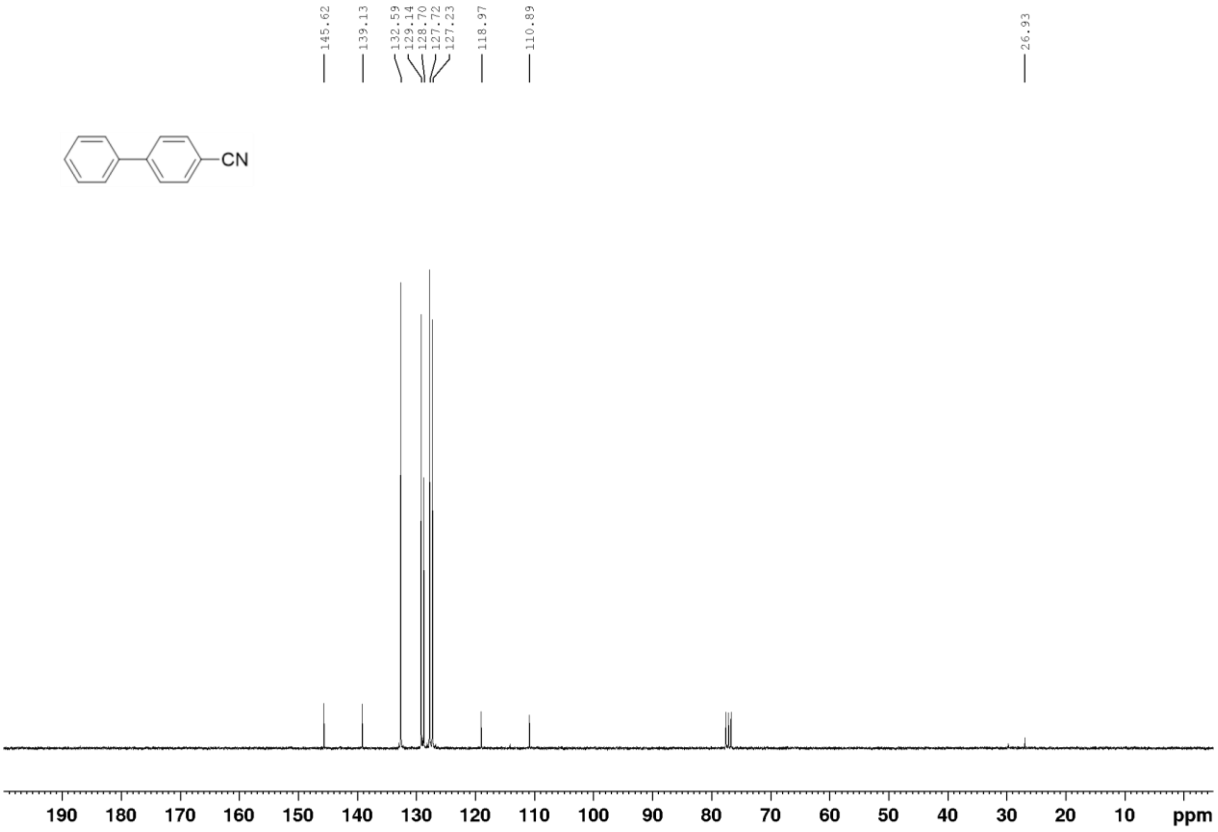
145.62
139.13
132.59
129.14
128.70
127.72
127.23
118.97
110.89
26.93
CN
190
180
170
160
150
140
130
120
110
100
90
80
70
60
50
40
30
20
10
ppm

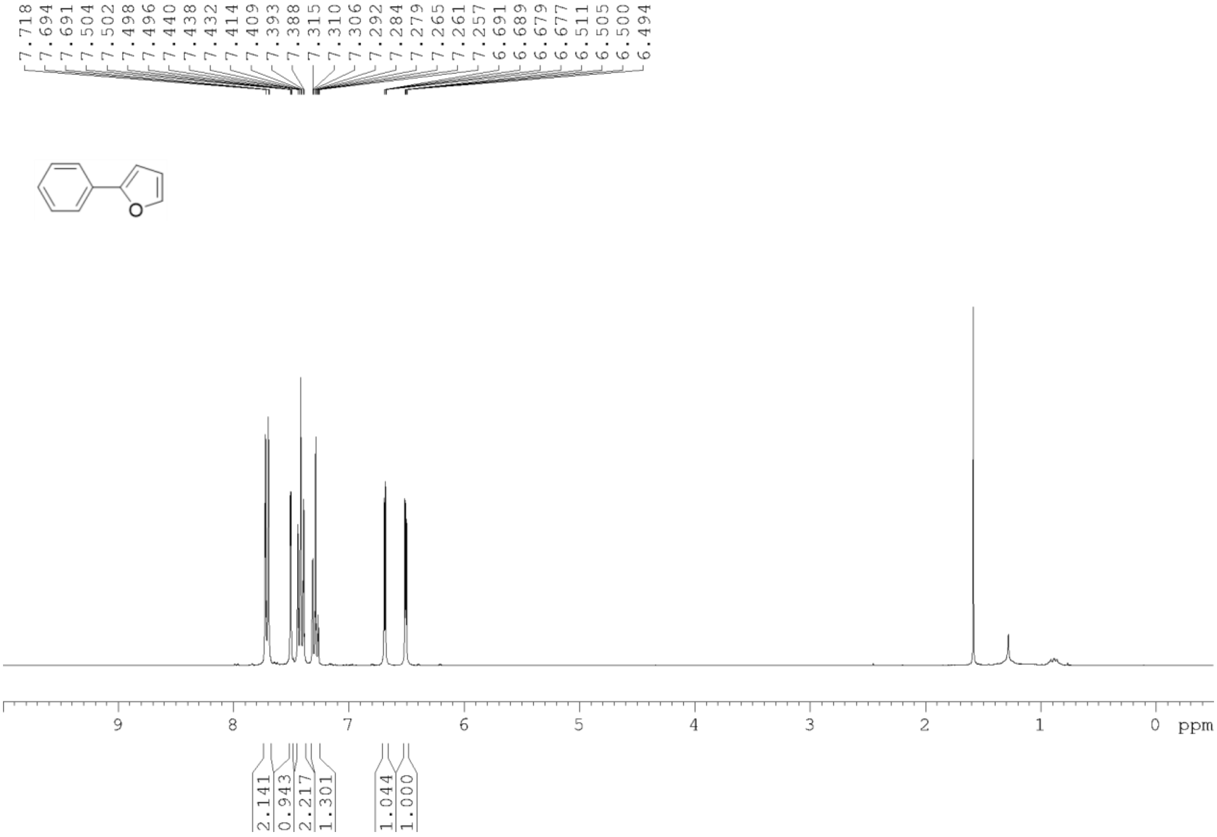
7.718
7.694
7.691
7.504
7.502
7.498
7.496
7.440
7.438
7.432
7.414
7.409
7.393
7.388
7.315
7.310
7.306
7.292
7.284
7.279
7.265
7.261
7.257
6.691
6.689
6.679
6.677
6.511
6.505
6.500
6.494
O
9 8 7 6 5 4 3 2 1 0 ppm
2.141
0.943
2.217
1.301
1.044
1.000

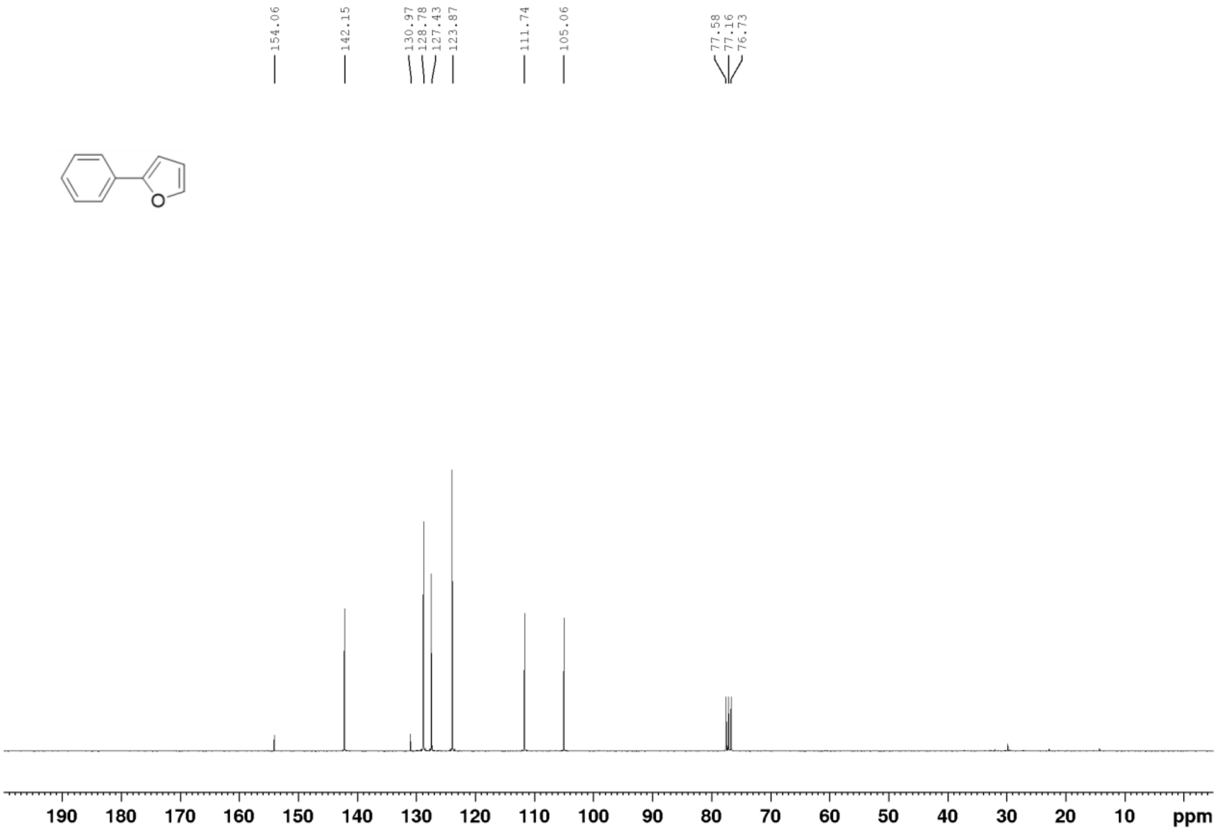
154.06
142.15
130.97
128.78
127.43
123.87
111.74
105.06
77.58
77.16
76.73
O
190 180 170 160 150 140 130 120 110 100 90 80 70 60 50 40 30 20 10 ppm

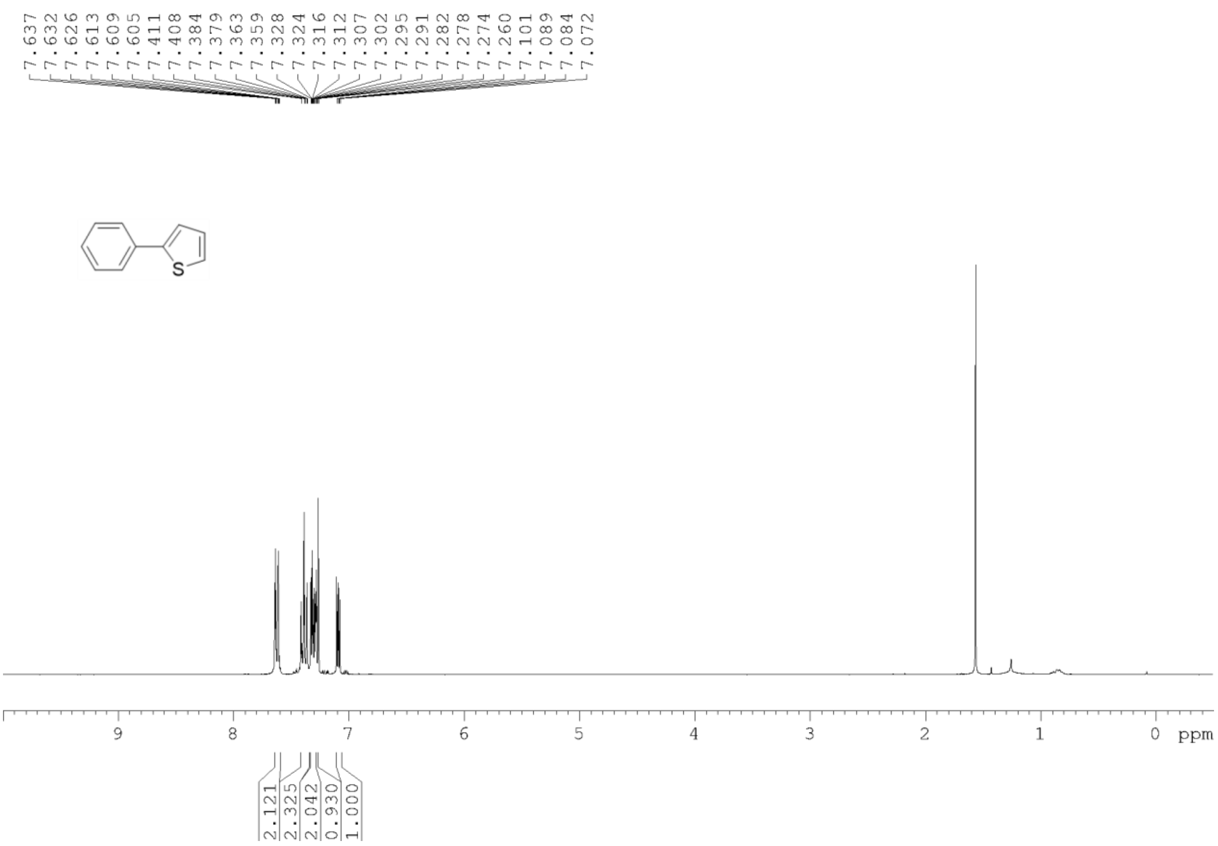
7.637
7.632
7.626
7.613
7.609
7.605
7.411
7.408
7.384
7.379
7.363
7.359
7.328
7.324
7.316
7.312
7.307
7.302
7.295
7.291
7.282
7.278
7.274
7.260
7.101
7.089
7.084
7.072
S
9 8 7 6 5 4 3 2 1 0 ppm
2.121
2.325
2.042
0.930
1.000

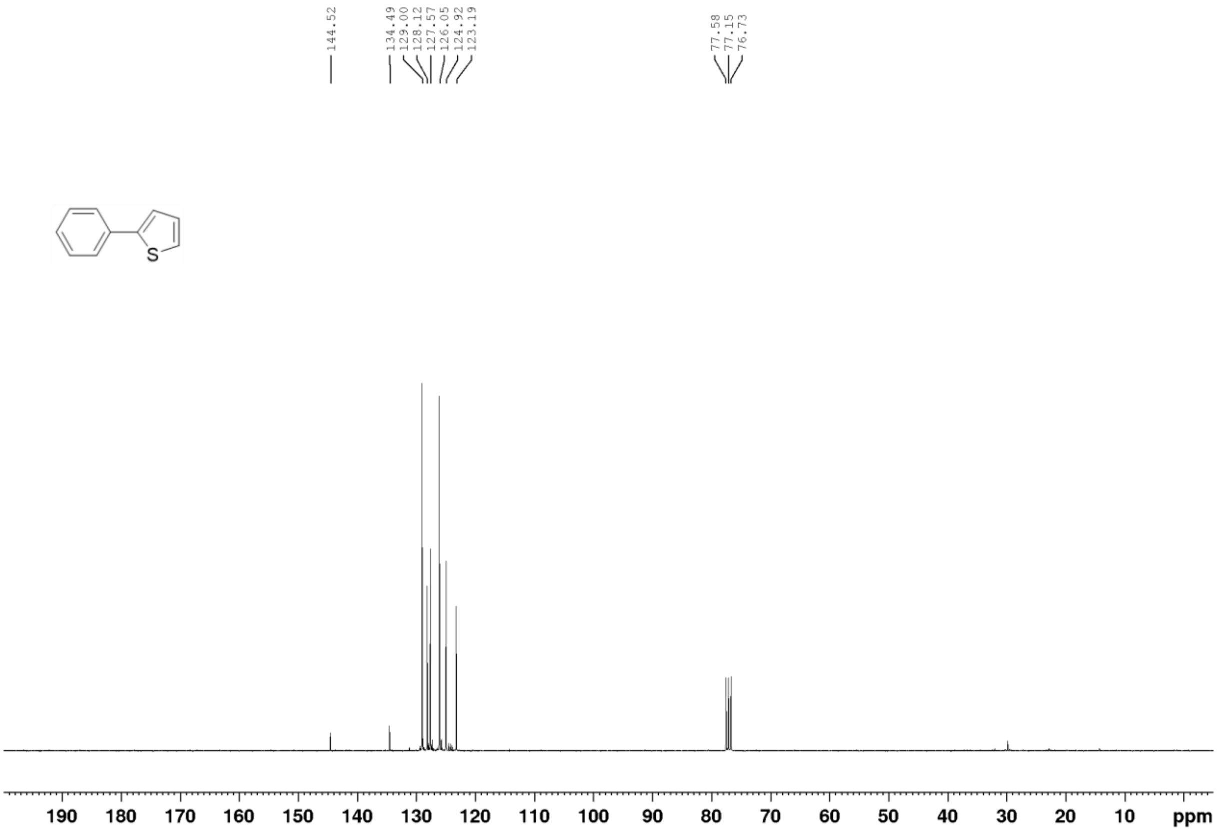
144.52
134.49
129.00
128.12
127.57
126.05
124.92
123.19
77.58
77.15
76.73
S
190 180 170 160 150 140 130 120 110 100 90 80 70 60 50 40 30 20 10 ppm

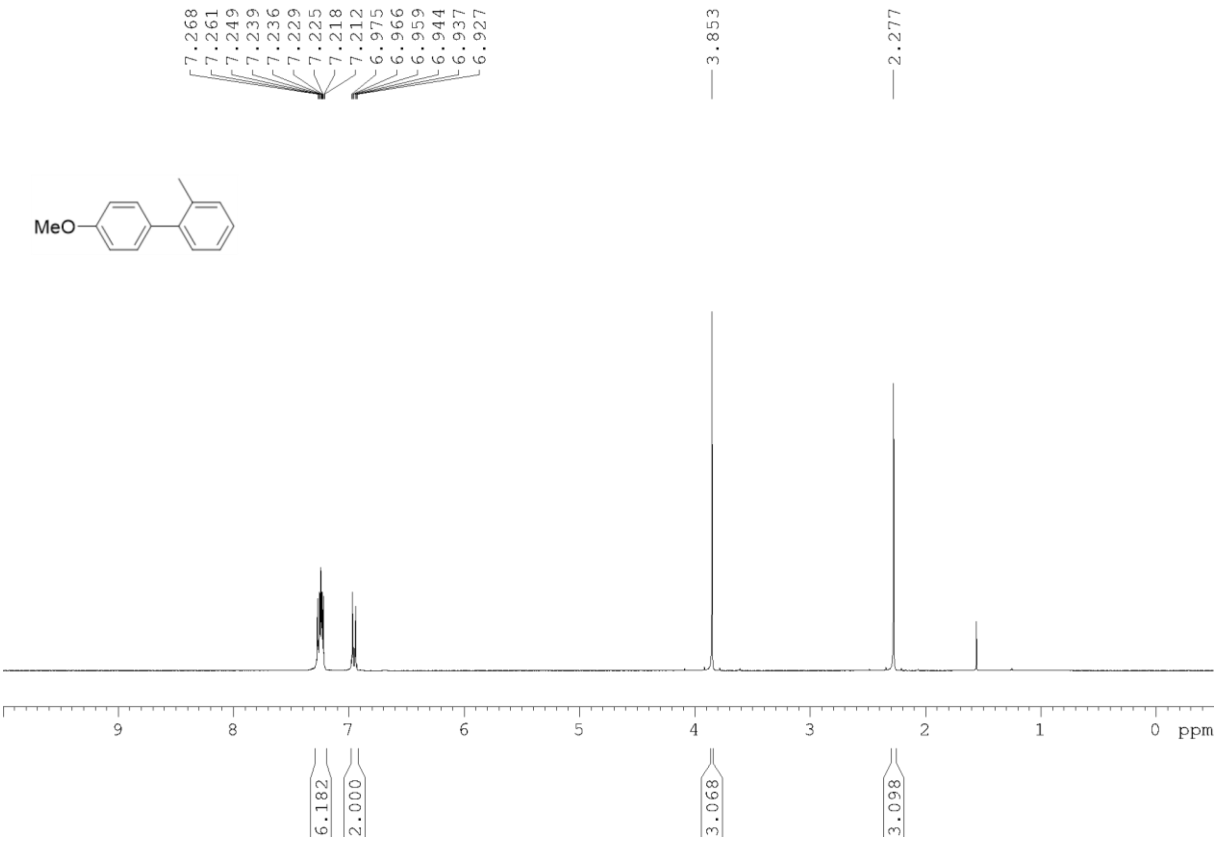
7.268
7.261
7.249
7.239
7.236
7.229
7.225
7.218
7.212
6.975
6.966
6.959
6.944
6.937
6.927
3.853
2.277
MeO
ppm
6.182
2.000
3.068
3.098

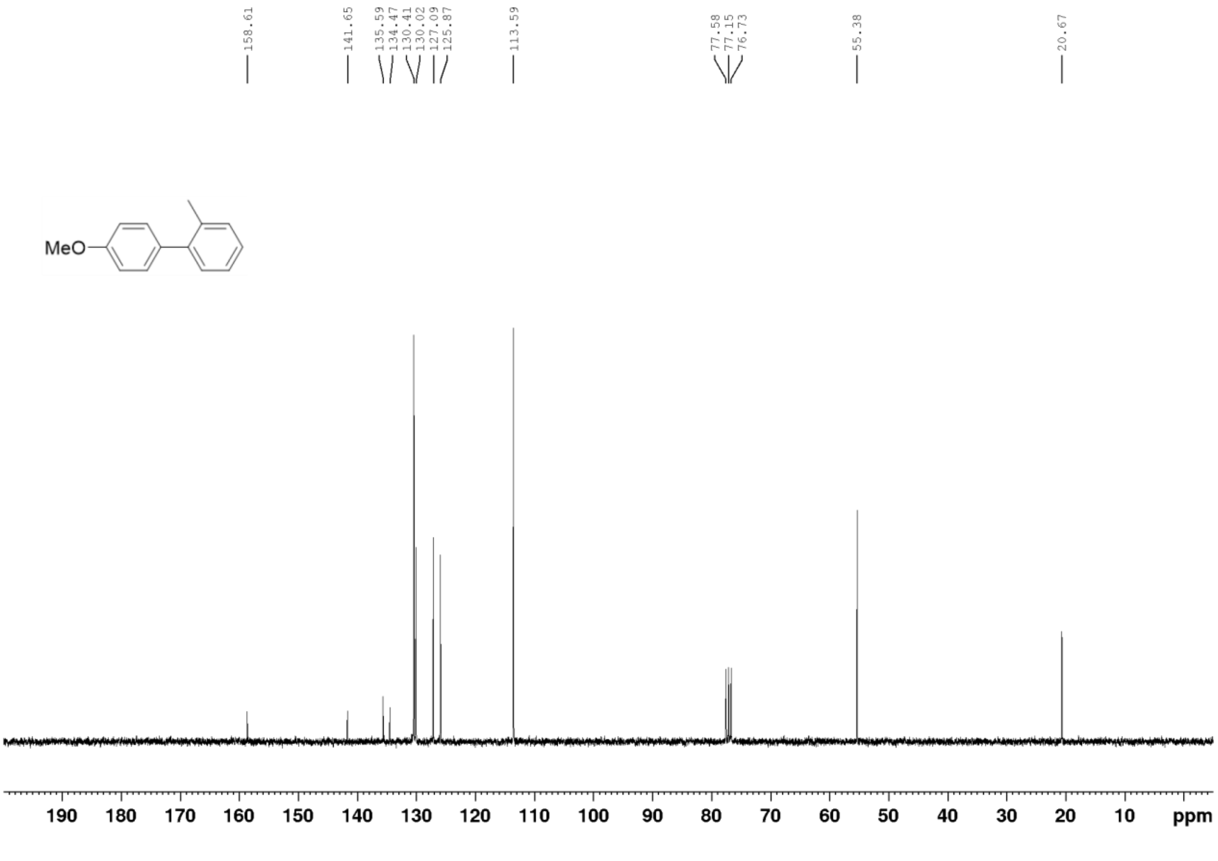
158.61
141.65
135.59
134.47
130.41
130.02
127.09
125.87
113.59
77.58
77.15
76.73
55.38
20.67
MeO
ppm

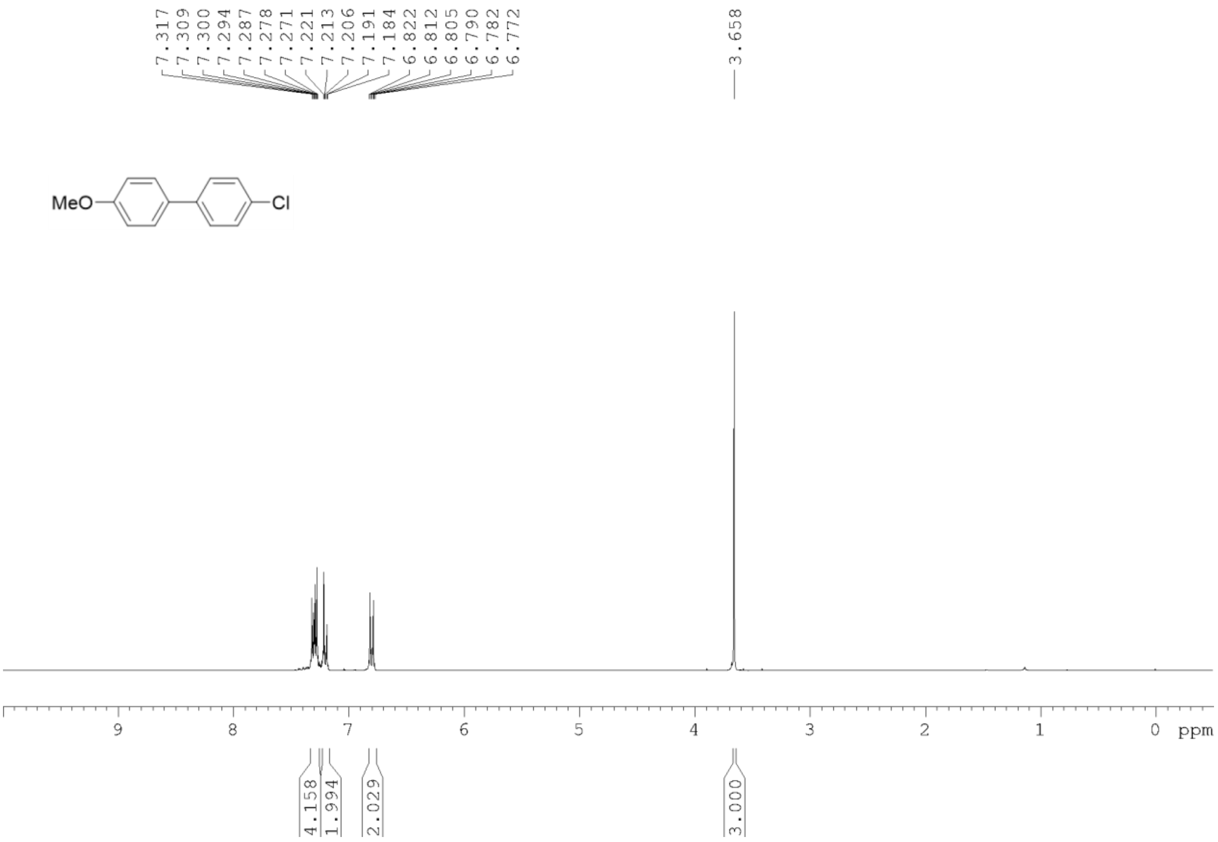
7.317
7.309
7.300
7.294
7.287
7.278
7.271
7.221
7.213
7.206
7.191
7.184
6.822
6.812
6.805
6.790
6.782
6.772
3.658
MeO
Cl
9
8
7
6
5
4
3
2
1
0 ppm
4.158
1.994
2.029
3.000

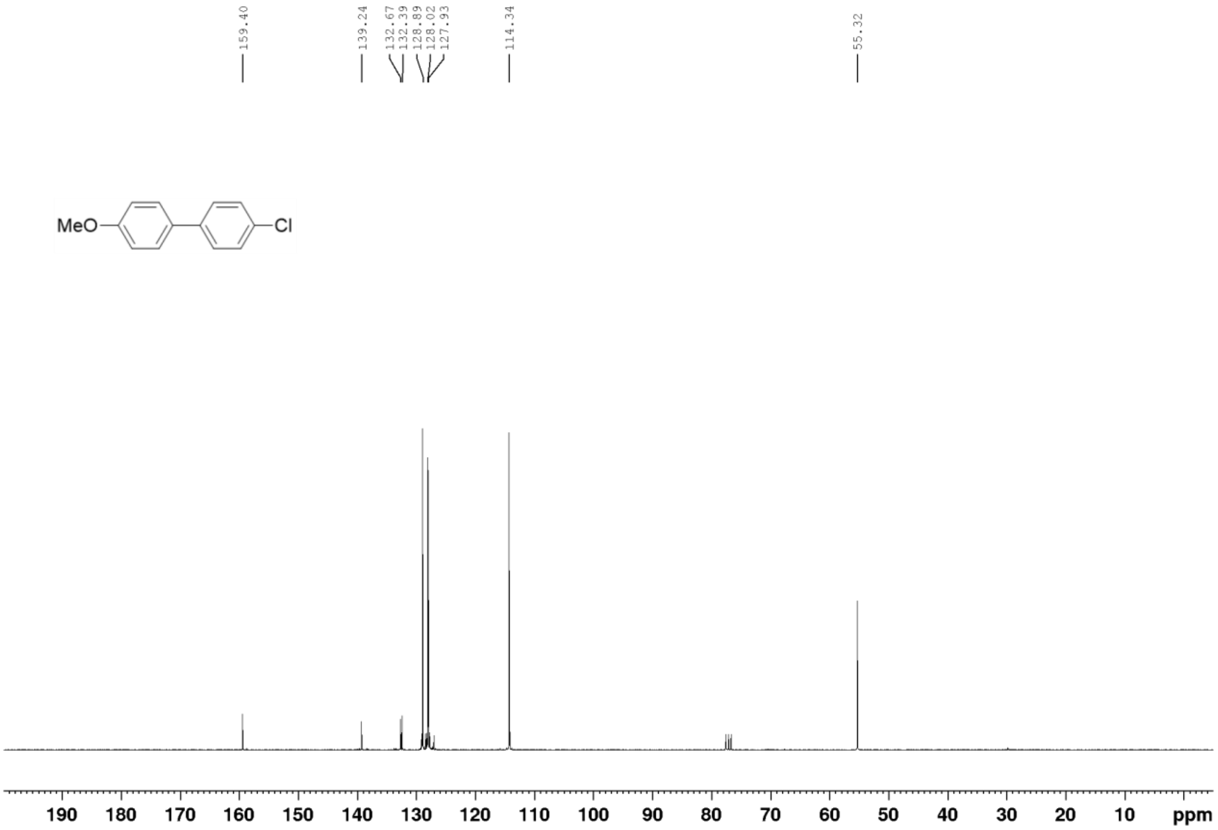
159.40
139.24
132.67
132.39
128.89
128.02
127.93
114.34
55.32
MeO
Cl
190
180
170
160
150
140
130
120
110
100
90
80
70
60
50
40
30
20
10
ppm

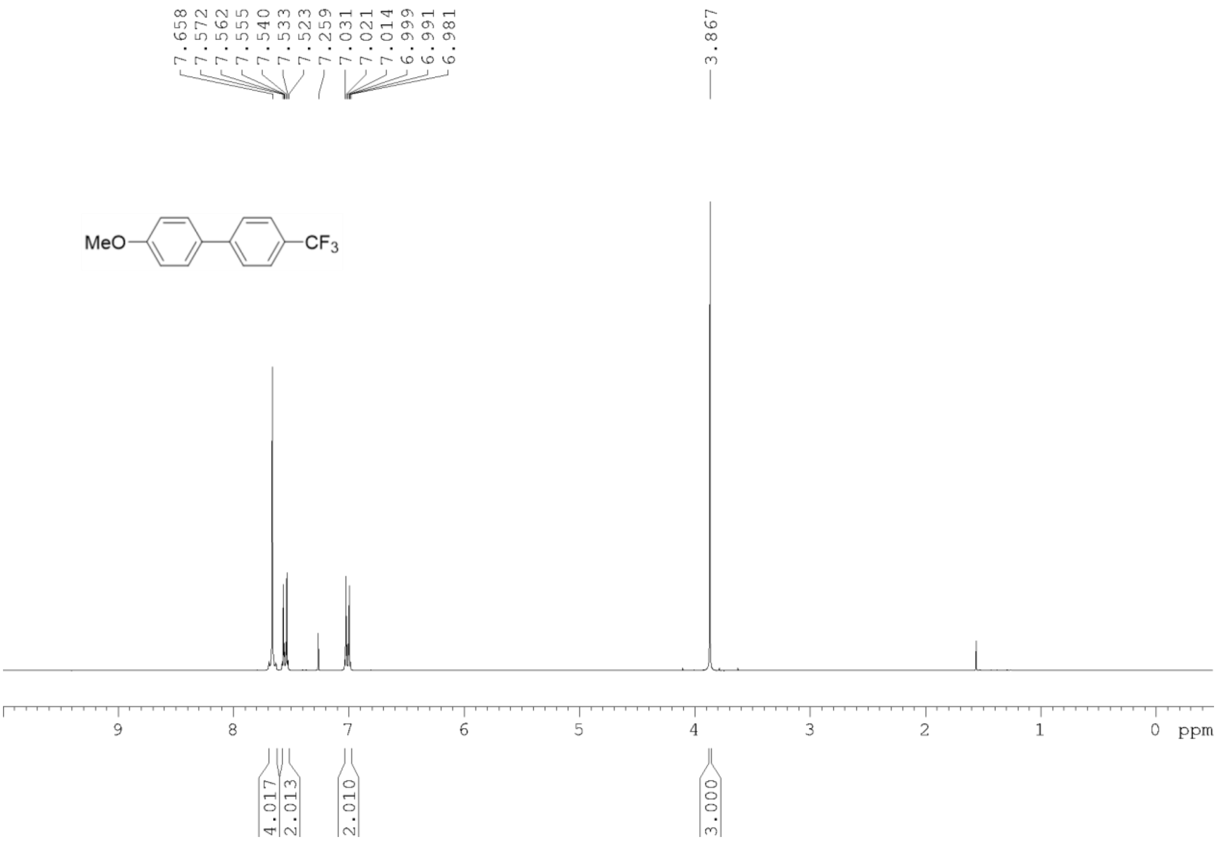
7.658
7.572
7.562
7.555
7.540
7.533
7.523
7.259
7.031
7.021
7.014
6.999
6.991
6.981
3.867
MeO
CF3
9
8
7
6
5
4
3
2
1
0 ppm
4.017
2.013
2.010
3.000

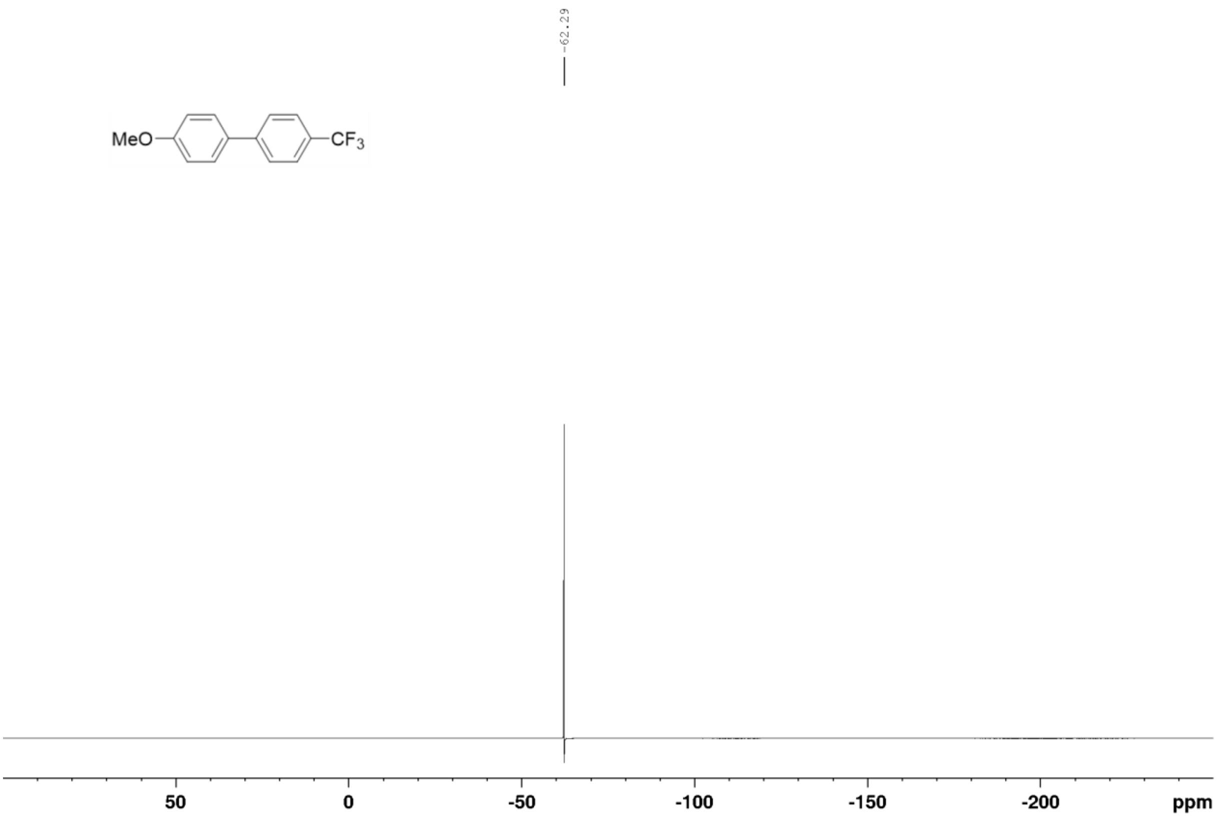
-62.29
MeO
CF3
50
0
-50
-100
-150
-200
ppm

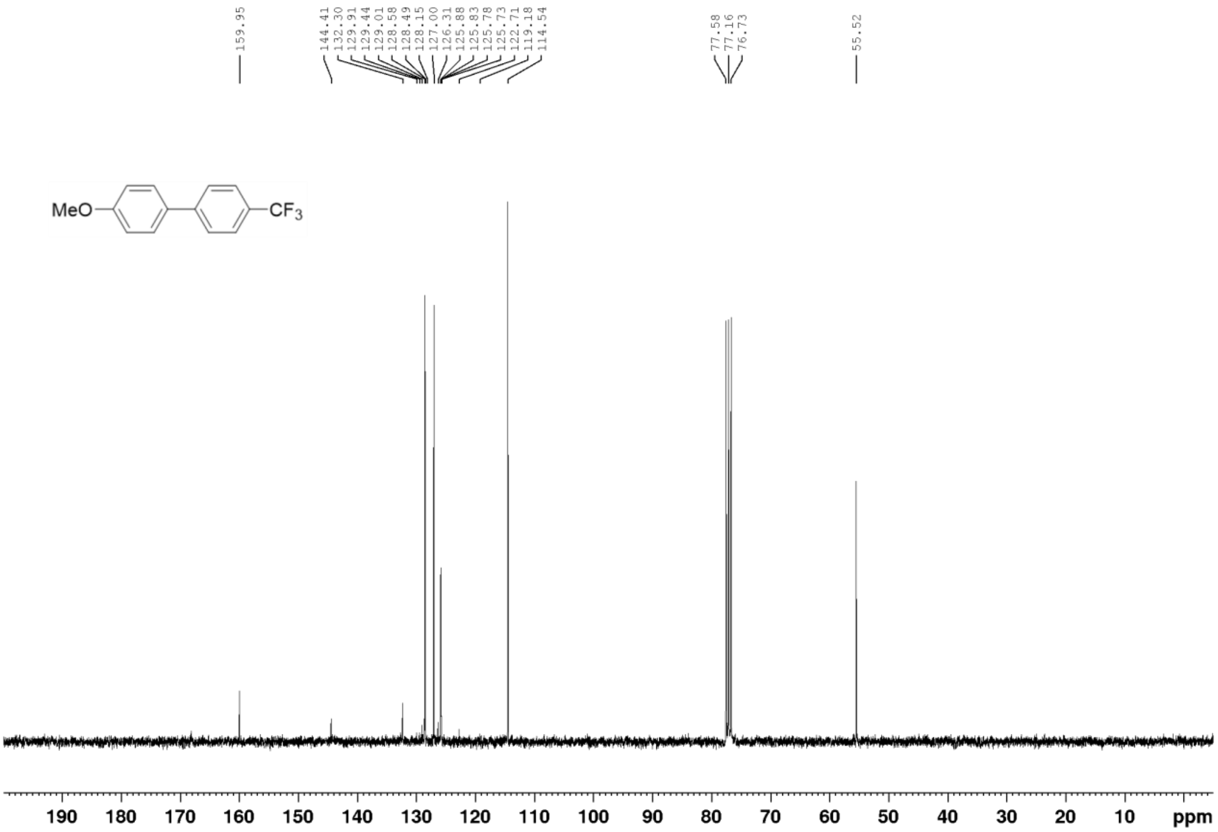
159.95
144.41
132.30
129.91
129.44
129.01
128.58
128.49
128.15
127.00
126.31
125.88
125.83
125.78
125.73
122.71
119.18
114.54
77.58
77.16
76.73
55.52
MeO
CF3
190
180
170
160
150
140
130
120
110
100
90
80
70
60
50
40
30
20
10
ppm

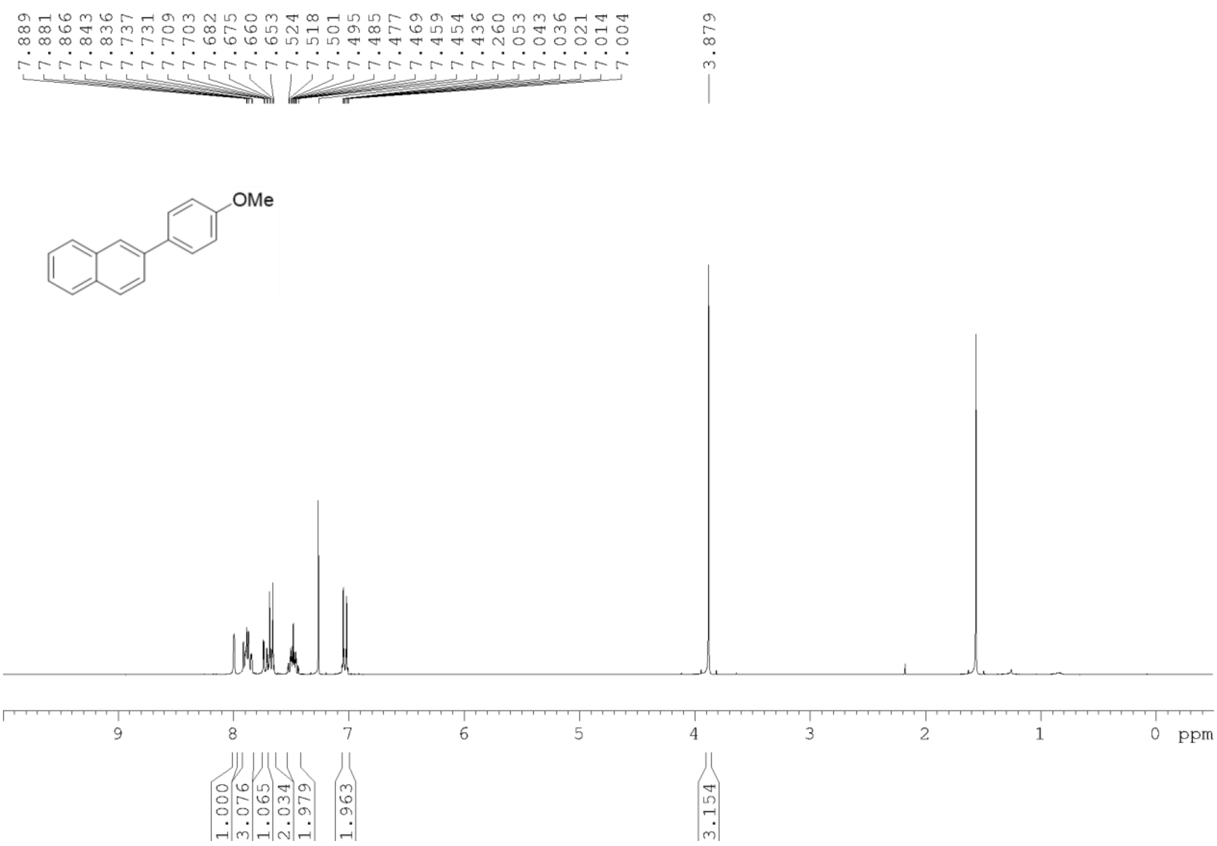
7.889
7.881
7.866
7.843
7.836
7.737
7.731
7.709
7.703
7.682
7.675
7.660
7.653
7.524
7.518
7.501
7.495
7.485
7.477
7.469
7.459
7.454
7.436
7.260
7.053
7.043
7.036
7.021
7.014
7.004
3.879
OMe
9
8
7
6
5
4
3
2
1
0 ppm
1.000
3.076
1.065
2.034
1.979
1.963
3.154

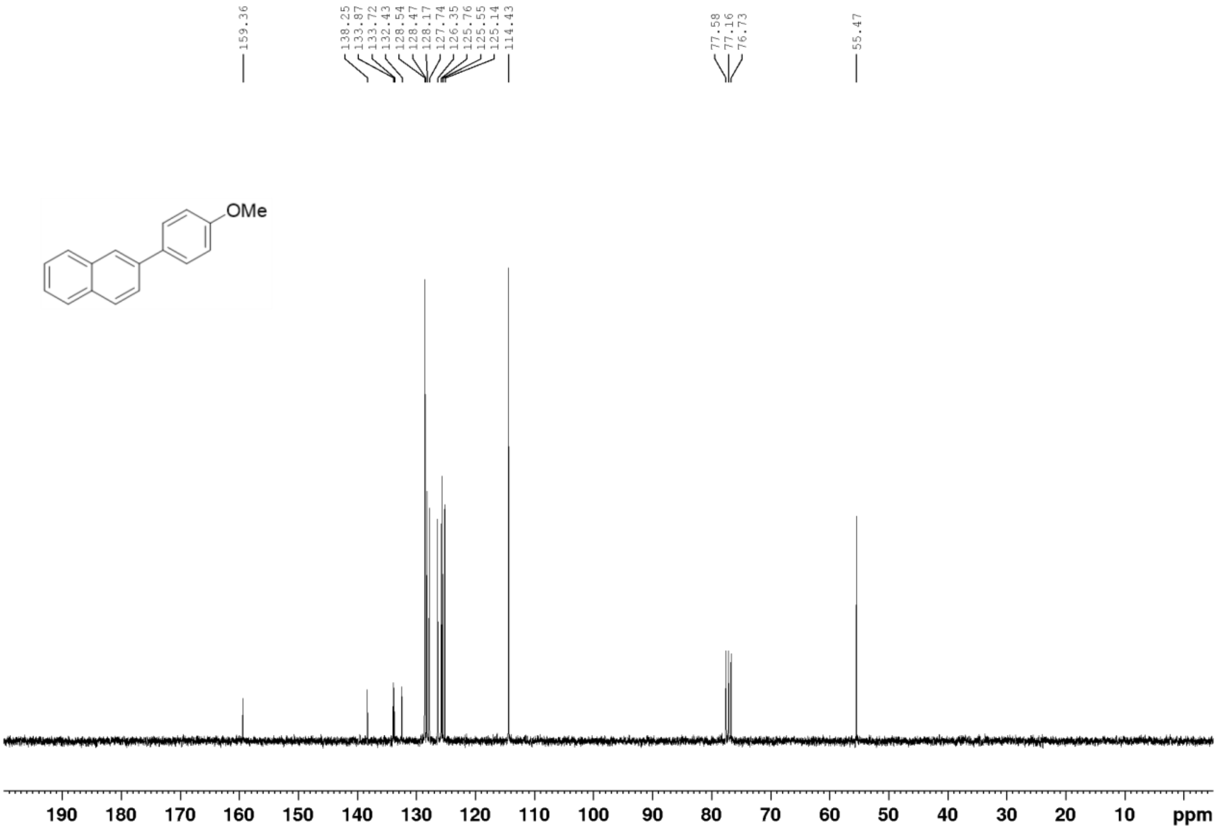
159.36
138.25
133.87
133.72
132.43
128.54
128.47
128.17
127.74
126.35
125.76
125.55
125.14
114.43
77.58
77.16
76.73
55.47
OMe
190
180
170
160
150
140
130
120
110
100
90
80
70
60
50
40
30
20
10
ppm

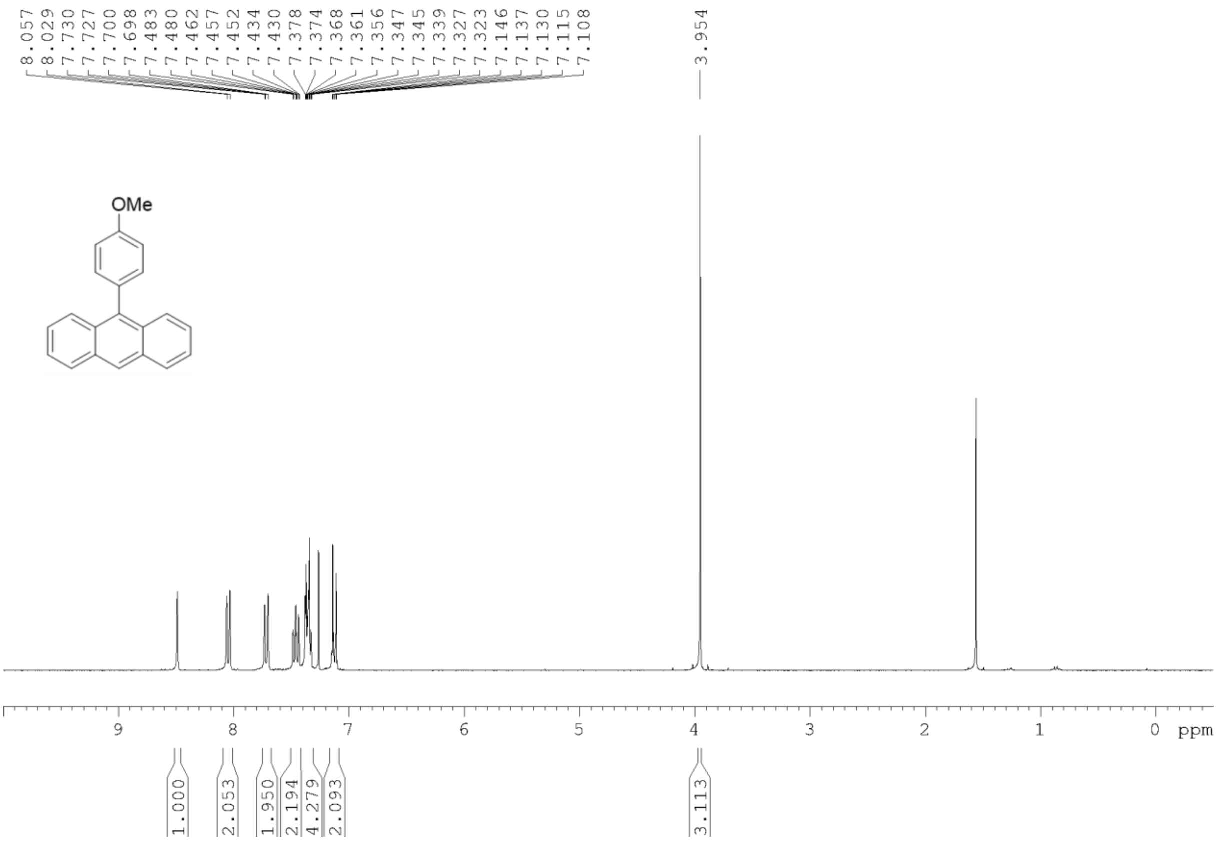
8.057
8.029
7.730
7.727
7.700
7.698
7.483
7.480
7.462
7.457
7.452
7.434
7.430
7.378
7.374
7.368
7.361
7.356
7.347
7.345
7.339
7.327
7.323
7.146
7.137
7.130
7.115
7.108
3.954
OMe
9 8 7 6 5 4 3 2 1 0 ppm
1.000
2.053
1.950
2.194
4.279
2.093
3.113

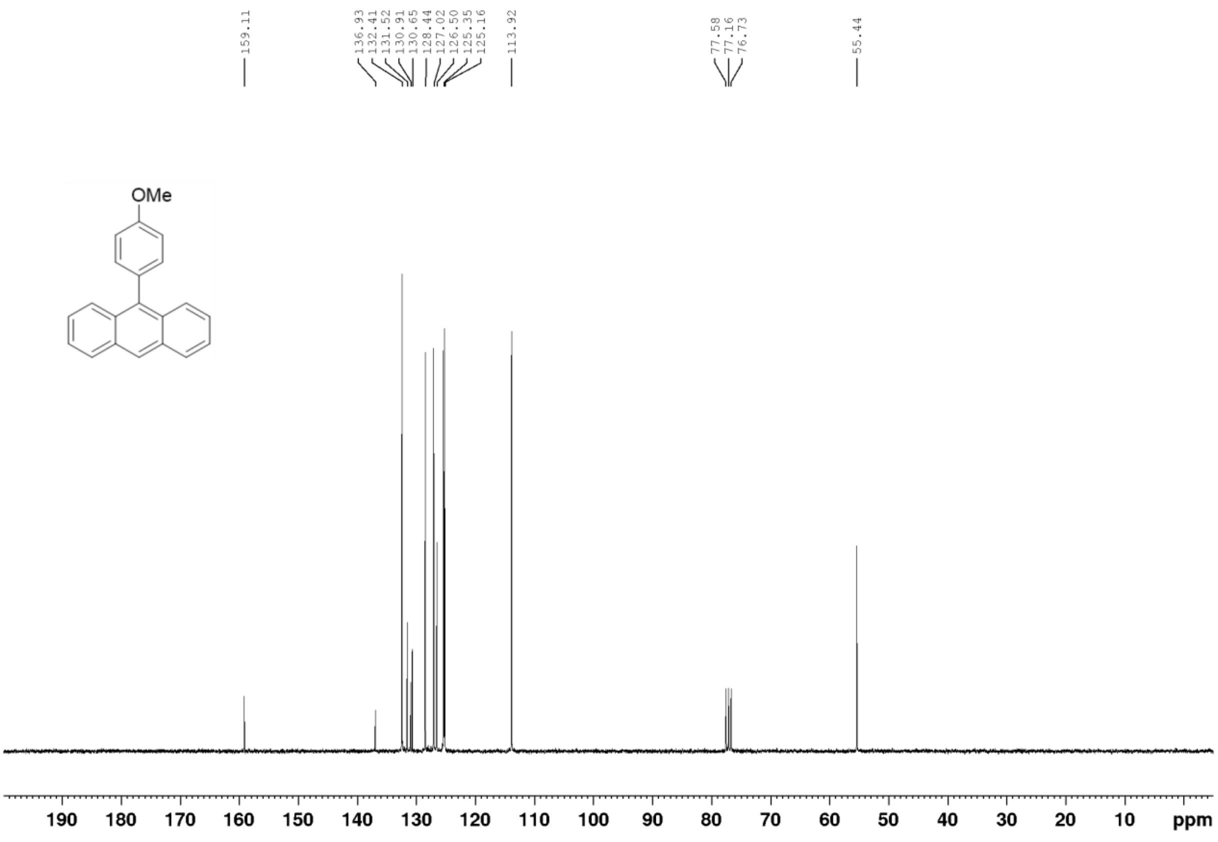
159.11
136.93
132.41
131.52
130.91
130.65
128.44
127.02
126.50
125.35
125.16
113.92
77.58
77.16
76.73
55.44
OMe
190 180 170 160 150 140 130 120 110 100 90 80 70 60 50 40 30 20 10 ppm

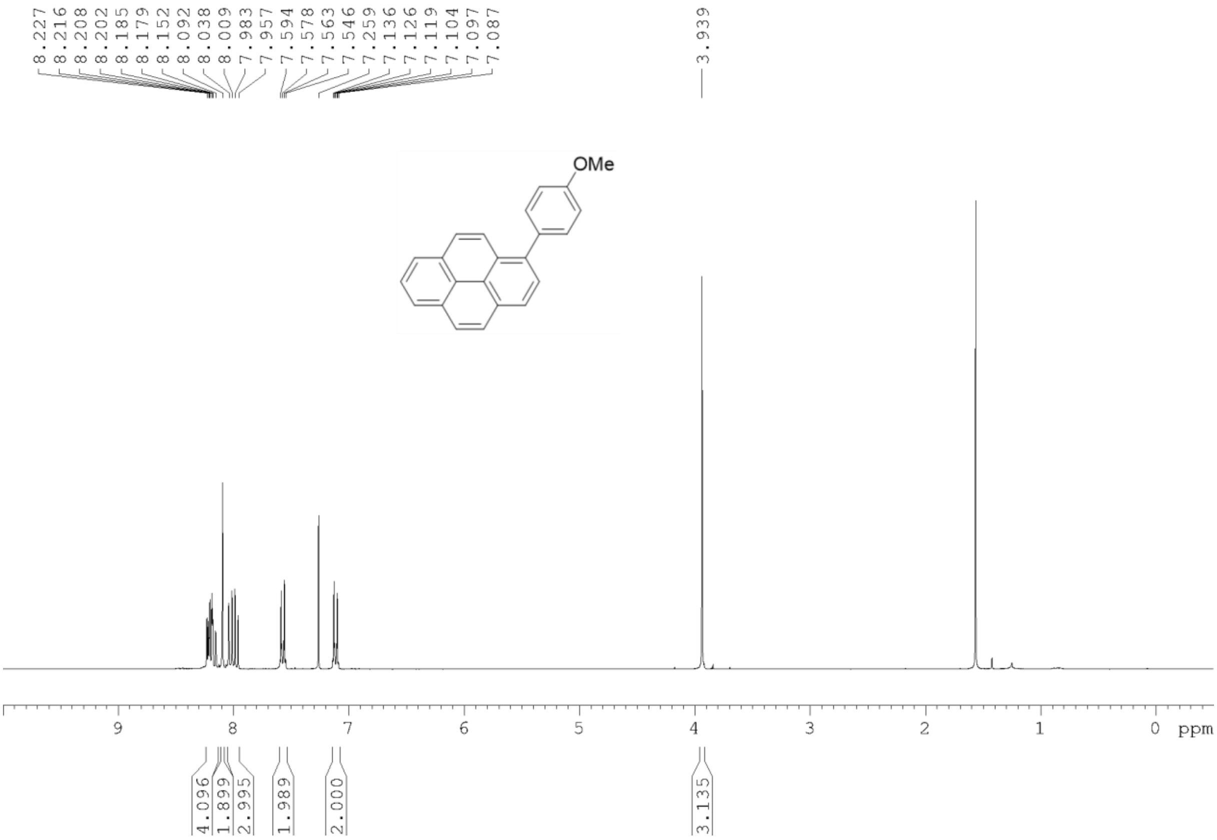
8.227
8.216
8.208
8.202
8.185
8.179
8.152
8.092
8.038
8.009
7.983
7.957
7.594
7.578
7.563
7.546
7.259
7.136
7.126
7.119
7.104
7.097
7.087
3.939
OMe
9
8
7
6
5
4
3
2
1
0 ppm
4.096
1.899
2.995
1.989
2.000
3.135

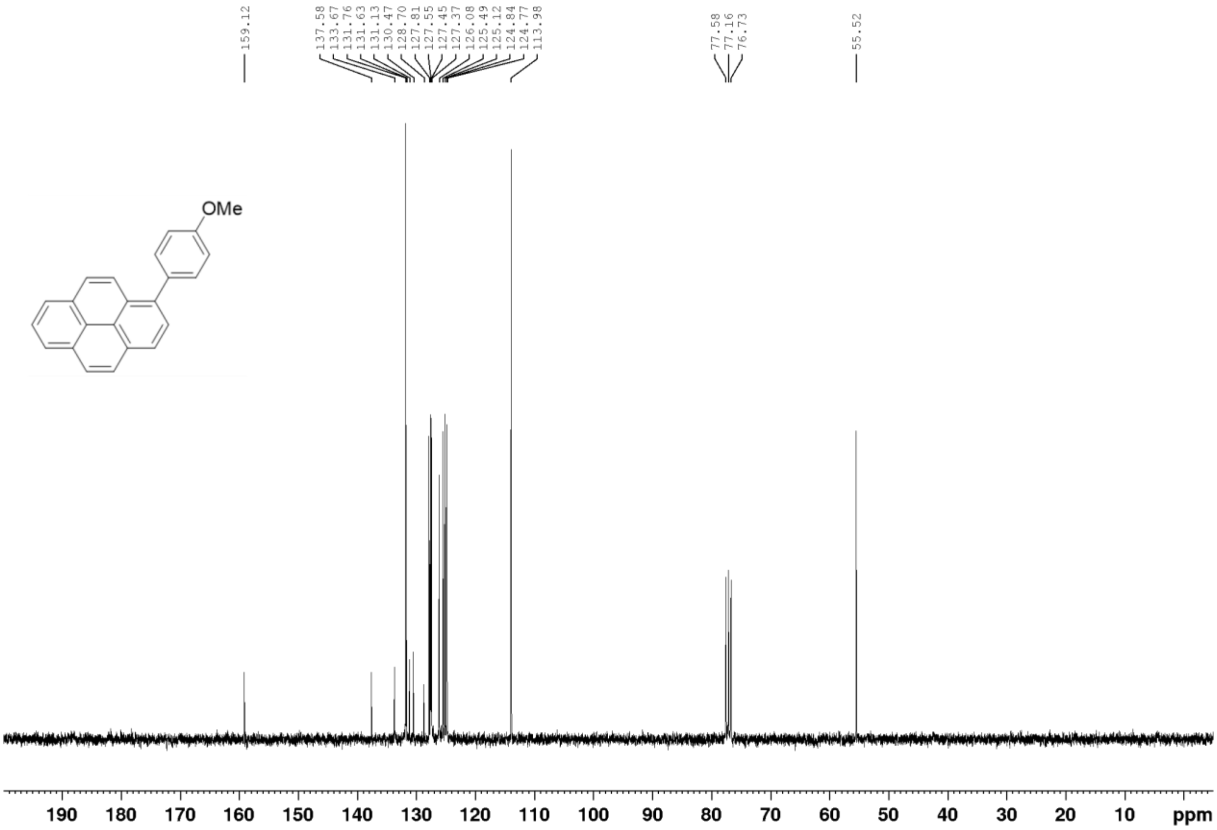
159.12
137.58
133.67
131.76
131.63
131.13
130.47
128.70
127.81
127.55
127.45
127.37
126.08
125.49
125.12
124.84
124.77
113.98
77.58
77.16
76.73
55.52
OMe
190
180
170
160
150
140
130
120
110
100
90
80
70
60
50
40
30
20
10
ppm

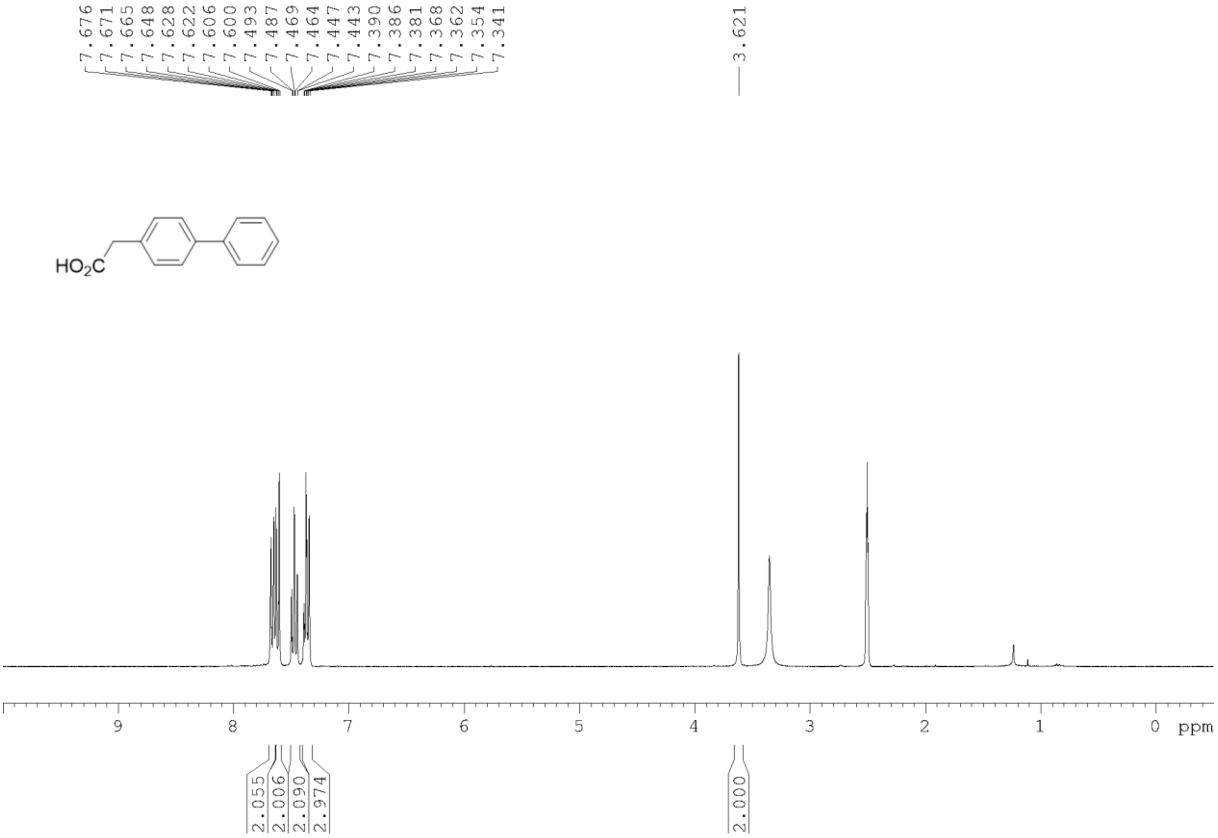
7.676 7.671 7.665 7.648 7.628 7.622 7.606 7.600 7.493 7.487 7.469 7.464 7.447 7.443 7.390 7.386 7.381 7.368 7.362 7.354 7.341
3.621
HO_2C
2.055 2.006 2.090 2.974
2.000
9 8 7 6 5 4 3 2 1 0 ppm

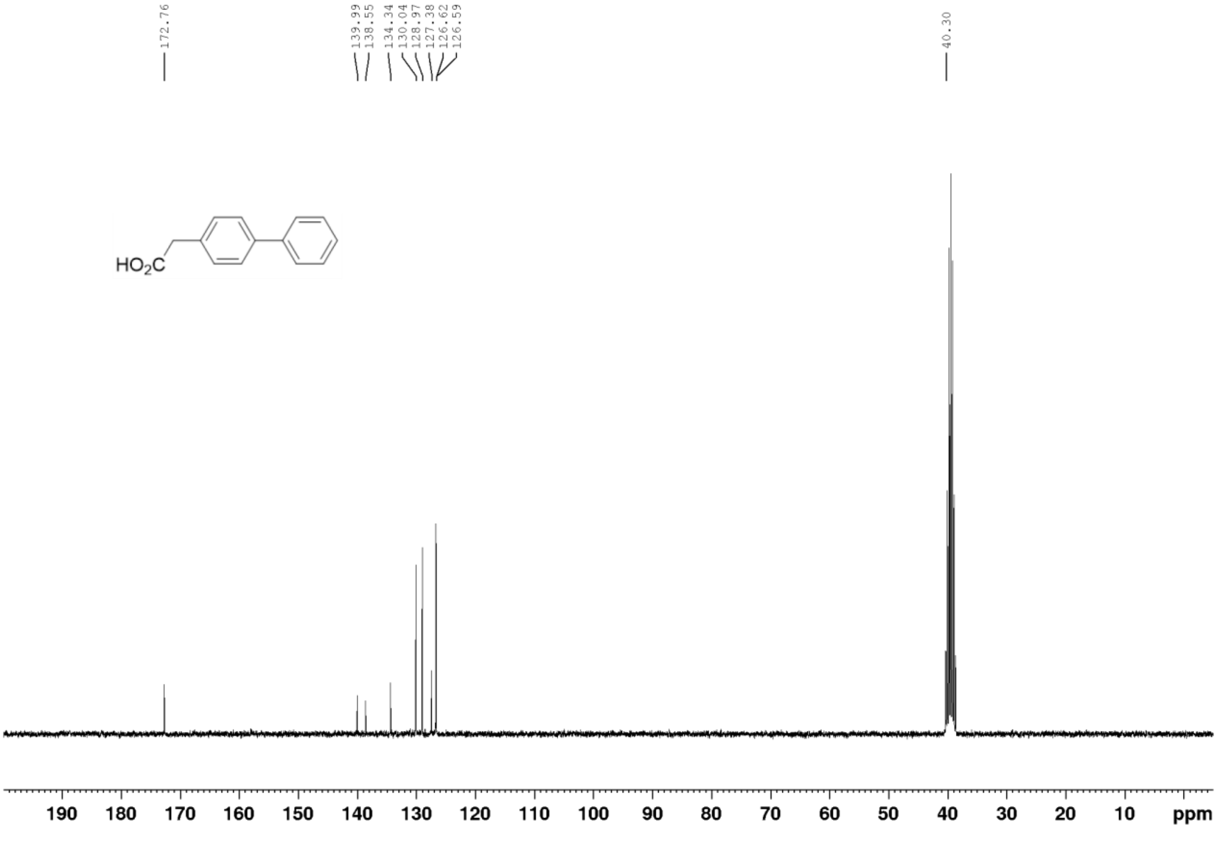
172.76
139.99 138.55 134.34 130.04 128.97 127.38 126.62 126.59
40.30
HO_2C
190 180 170 160 150 140 130 120 110 100 90 80 70 60 50 40 30 20 10 ppm

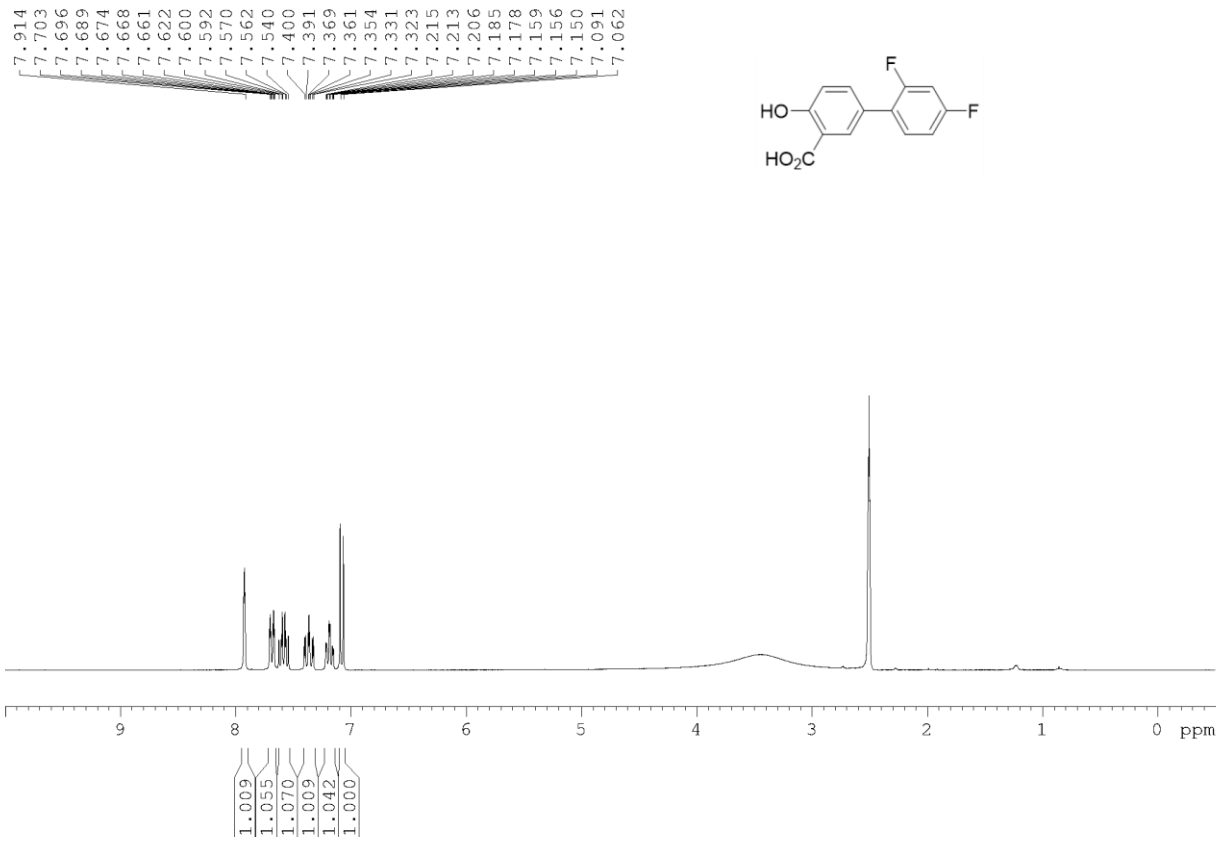
7.914
7.703
7.696
7.689
7.674
7.668
7.661
7.622
7.600
7.592
7.570
7.562
7.540
7.400
7.391
7.369
7.361
7.354
7.331
7.323
7.215
7.213
7.206
7.185
7.178
7.159
7.156
7.150
7.091
7.062
HO
HO2C
F
F
9
8
7
6
5
4
3
2
1
0 ppm
1.009
1.055
1.070
1.009
1.042
1.000

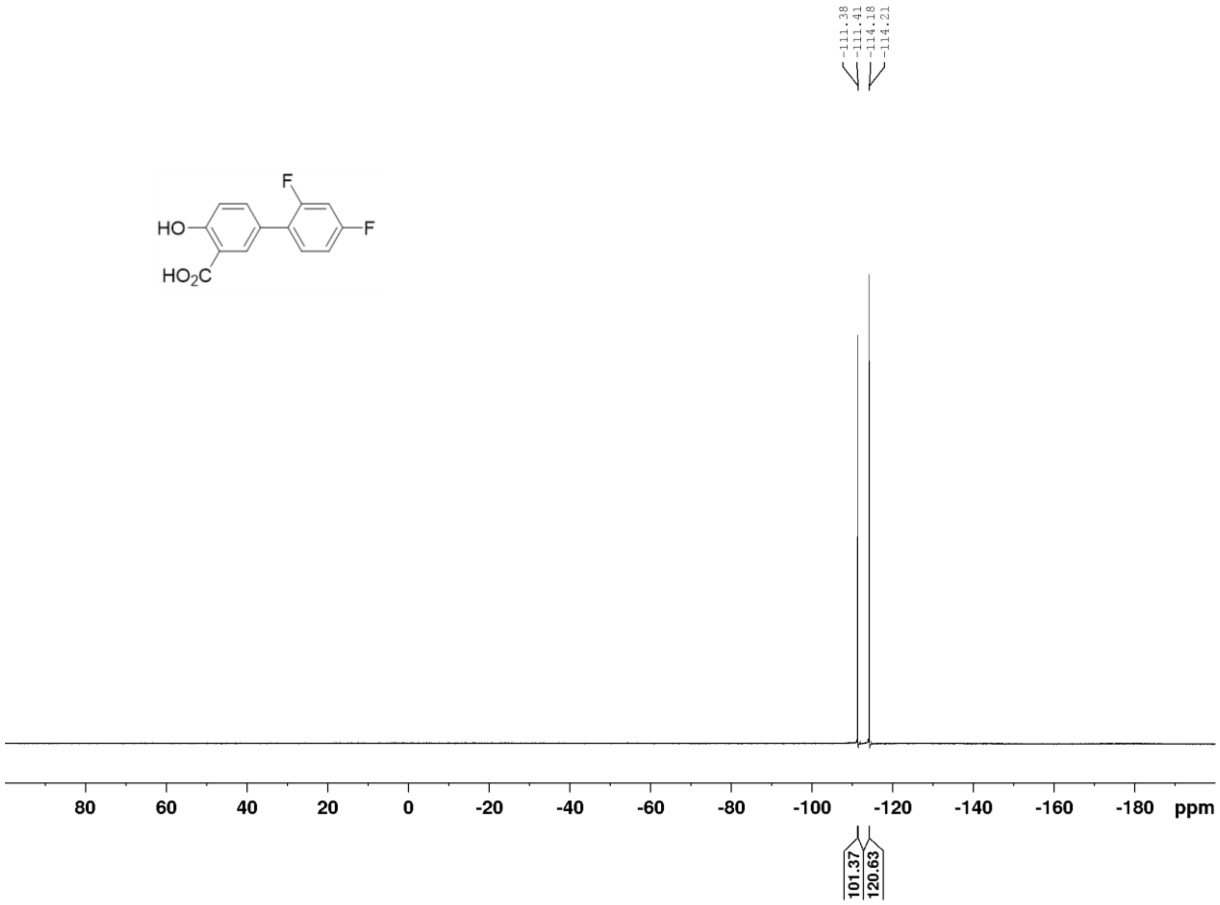
-111.38
-111.41
-114.18
-114.21
HO
HO2C
F
F
80
60
40
20
0
-20
-40
-60
-80
-100
-120
-140
-160
-180
ppm
101.37
120.63

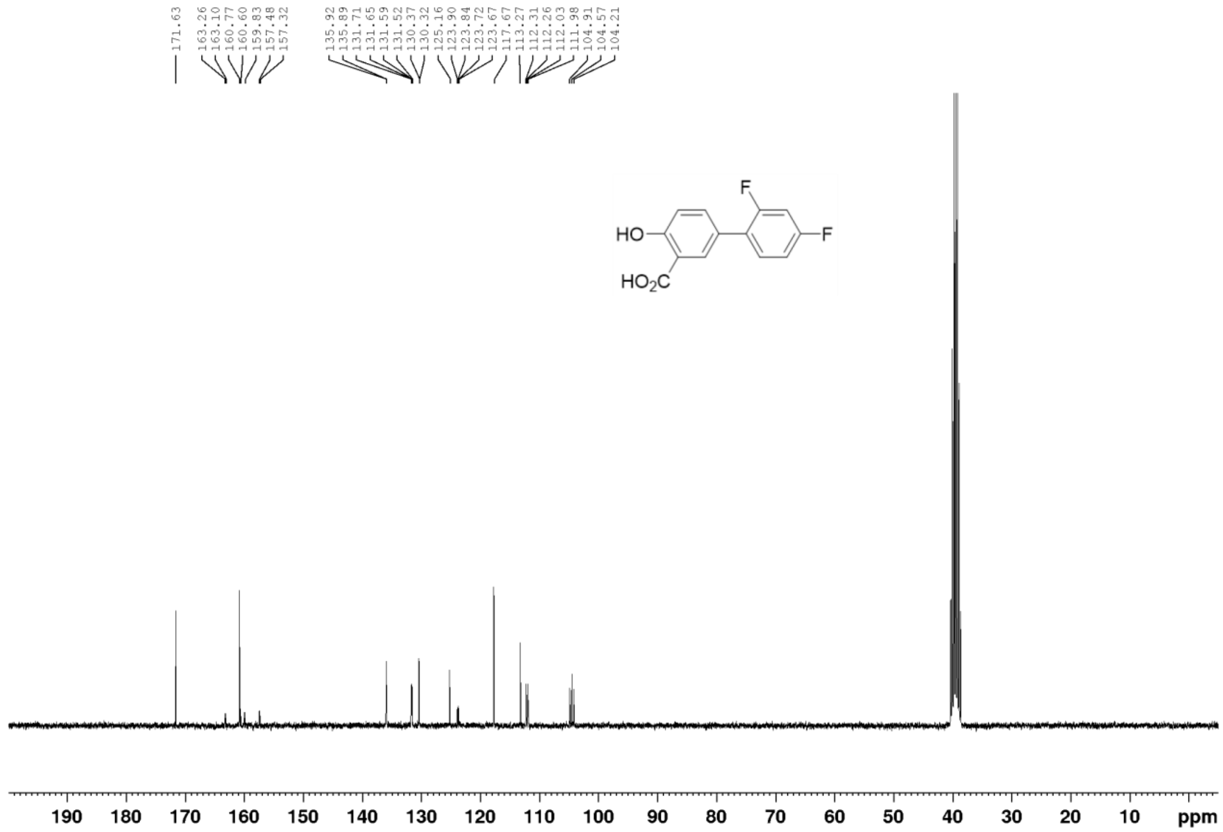
171.63
163.26
163.10
160.77
160.60
159.83
157.48
157.32
135.92
135.89
131.71
131.65
131.59
131.52
130.37
130.32
125.16
123.90
123.84
123.72
123.67
117.67
113.27
112.31
112.26
112.03
111.98
104.91
104.57
104.21
HO
HO2C
F
F
190 180 170 160 150 140 130 120 110 100 90 80 70 60 50 40 30 20 10 ppm

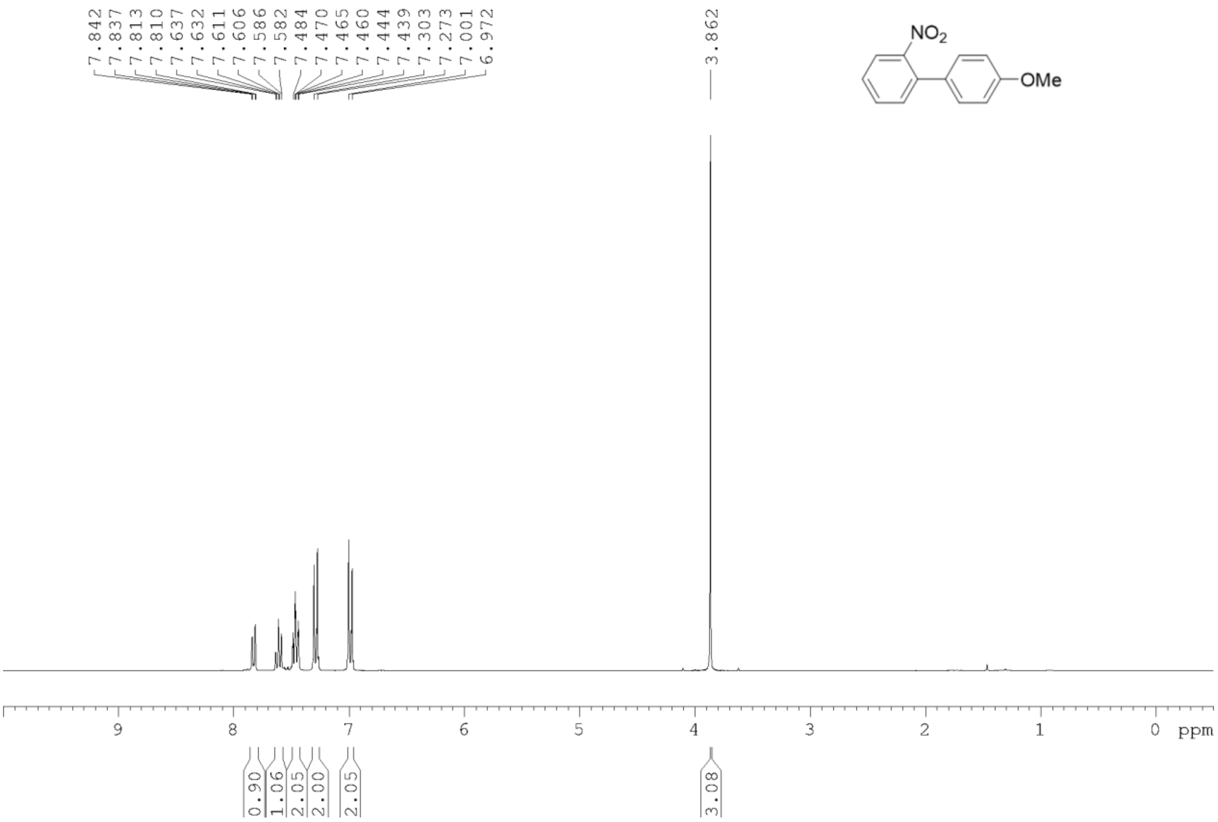
7.842
7.837
7.813
7.810
7.637
7.632
7.611
7.606
7.586
7.582
7.484
7.470
7.465
7.460
7.444
7.439
7.303
7.273
7.001
6.972
3.862
NO2
OMe
9
8
7
6
5
4
3
2
1
0 ppm
0.90
1.06
2.05
2.00
2.05
3.08

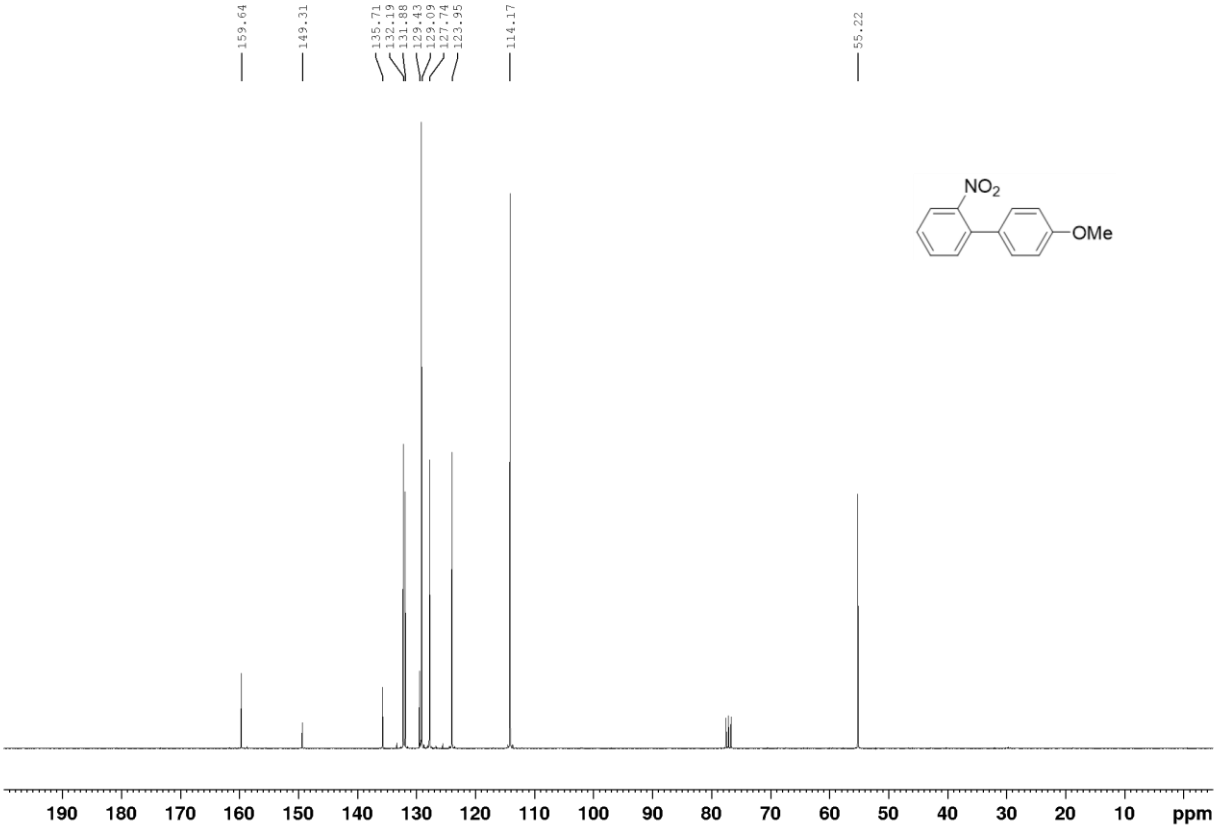
159.64
149.31
135.71
132.19
131.88
129.43
129.09
127.74
123.95
114.17
55.22
NO2
OMe
190
180
170
160
150
140
130
120
110
100
90
80
70
60
50
40
30
20
10
ppm

9. References

[43] C. R. McElroy, A. Constantinou, L. C. Jones, L. Summerton, J. H. Clark, *Green Chem* **2015**, *17*, 3111–3121.

[50] J.-H. Na, X. Liu, J.-W. Jing, J. Wang, X.-Q. Chu, M. Ma, H. Xu, X. Zhou, Z.-L. Shen, *Org Lett* **2023**, *25*, 2318–2322.

[51] F. Mäsing, H. Nüsse, J. Klingauf, A. Studer, *Org Lett* **2018**, *20*, 752–755.

[52] M. Ravbar, A. Koler, M. Paljevac, P. Krajnc, M. Kolar, J. Iskra, *ACS Omega* **2022**, *7*, 12610–12616.

[53] Y. Cheng, X. Gu, P. Li, *Org Lett* **2013**, *15*, 2664–2667.

[54] C. Premi, N. Jain, *Eur J Org Chem* **2013**, *2013*, 5493–5499.

[55] G. Zhang, X. Luo, C. Guan, Y. Cui, C. Ding, *Eur J Org Chem* **2023**, *26*, e202300114.

[56] C. Li, Y. Shi, Q. Chen, K. Zhang, G. Yang, *J Org Chem* **2023**, *88*, 2306–2313.

[57] T. Seo, T. Ishiyama, K. Kubota, H. Ito, *Chem Sci* **2019**, *10*, 8202–8210.

[58] G. A. Edwards, M. A. Trafford, A. E. Hamilton, A. M. Buxton, M. C. Bardeaux, J. M. Chalker, *J Org Chem* **2014**, *79*, 2094–2104.

[59] R. B. DeVasher, L. R. Moore, K. H. Shaughnessy, *J Org Chem* **2004**, *69*, 7919–7927.

[60] V. Arun, P. O. V. Reddy, M. Pilania, D. Kumar, *Eur J Org Chem* **2016**, *2016*, 2096–2100.
